# Supplementary material for: A Human Papillomavirus-Independent Cervical Cancer Animal Model Reveals Unconventional Mechanisms of Cervical Carcinogenesis
Source: Cell Rep. Author manuscript; Available in PMC 2019 Oct 24. (PMC6812687; doi:10.1016/j.celrep.2019.02.004)
Supplement: 1 [file NIHMS1523267-supplement-1.pdf]

Cell Reports, Volume *26*

Supplemental Information

A Human Papillomavirus-Independent Cervical Cancer Animal Model Reveals Unconventional Mechanisms of Cervical Carcinogenesis

Chunbo He, Xiangmin Lv, Cong Huang, Peter C. Angeletti, Guohua Hua, Jixin Dong, Jin Zhou, Zhengfeng Wang, Bowen Ma, Xingcheng Chen, Paul F. Lambert, Bo R. Rueda, John S. Davis, and Cheng Wang

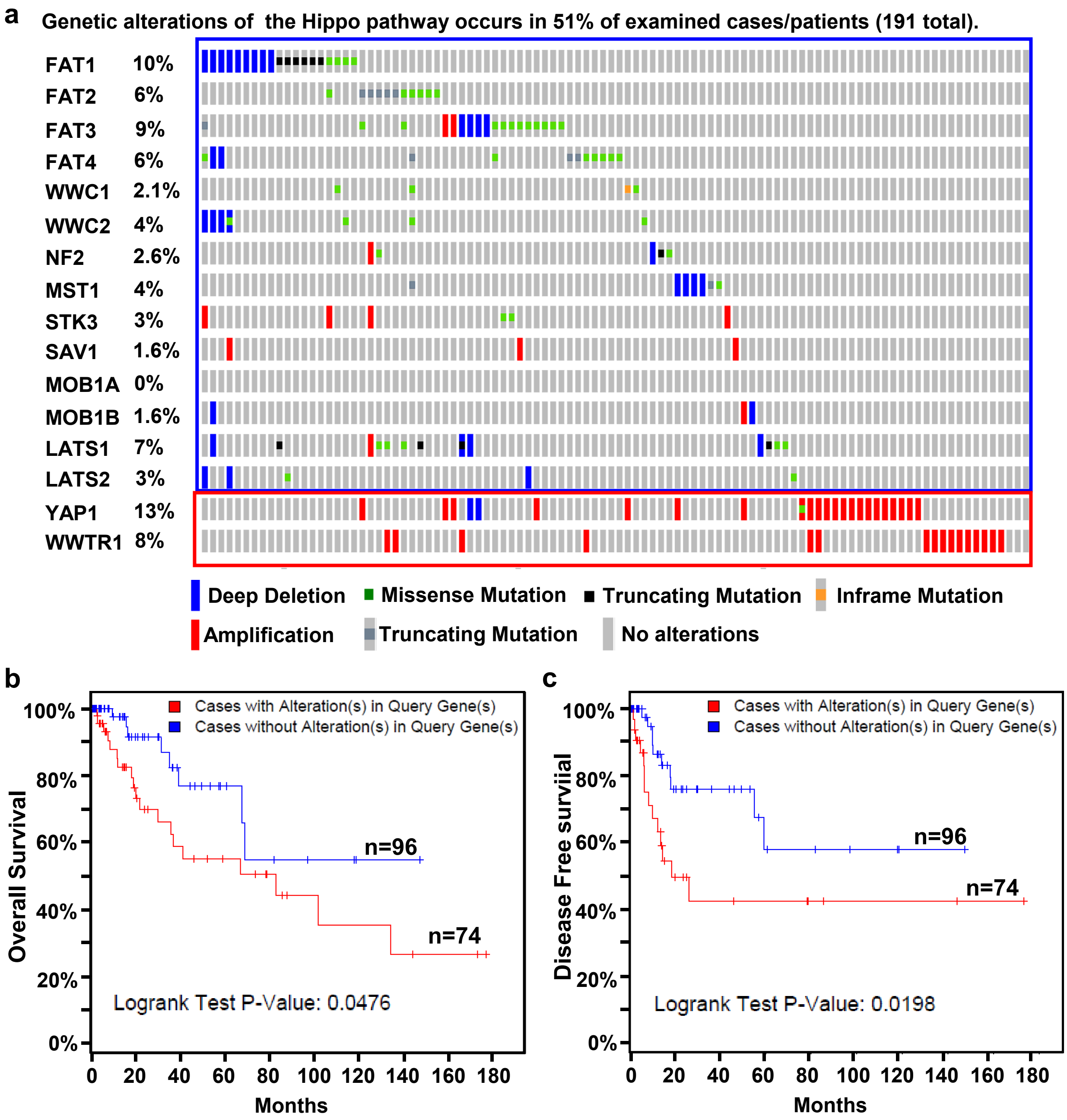

Figure S1. Alteration of genes encoding the key components of the Hippo pathway in cervical cancer patients. Related to Figure 1. a) Deletion and mutation of the upstream tumor suppressive genes (*MST1, LATS1/2*, and *FAT1/2/3/4*, *etc.*) and amplification of the downstream tumorigenic effectors (*YAP1* & *WWTR1*) of the Hippo pathway are frequently (51%) observed in 191 sequenced cervical cancer patient samples. Data were extracted from TCGA datasets with permission and analyzed using cBioPortal online tools (http://www.cbioportal.org/). **b & c)** Kaplan-Meier curves showing correlations between overall survival (**b**) / disease free survial (**C**) and genetic alteration of the Hippo/YAP pathway. The TCGA patient samples with survival information were stratified into two groups based on the genetic alteration of the Hippo signaling pathway: cases/patients with amplification of *YAP1* or deletion / mutation of tumor suppressors (*FAT1/2/3/4*, *MST1*, *LATS1/2*) in query genes (blue line, n=96) and cases/patients without genetic alterations in query genes (red line, n=74). Please note that patients with genetic alterations of the Hippo/ YAP pathway have poorer outcomes.

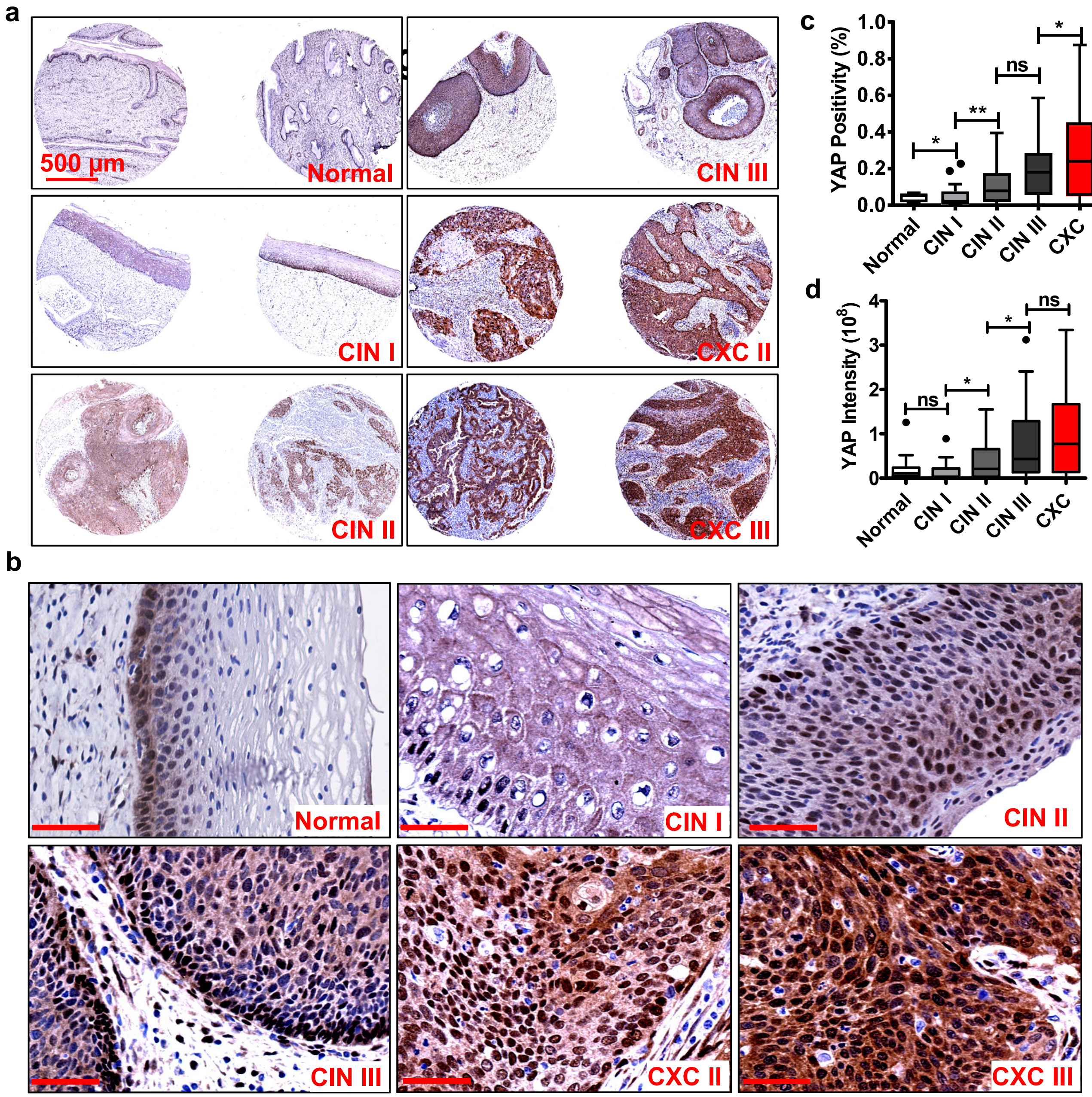

Figure S2. YAP1 expression is associated with cervical cancer initiation and progression. Related to Figure 1. **a)** Representative images showing the expression of YAP1 in normal cervical tissues, low and high grade cervical intraepithelial neoplasia (CIN), early-stage cervical cancer (II), and advanced-stage cervical cancer (stage III) tissues. Note the significant increase of YAP1 immunosignals (brown staining) with cervical cancer progression. Scale bar: 0.5 mm. **b**) High resolution images showing the relative level and cellular location of YAP1 protein in tissues derived from age-matched normal control, different stages of CIN and invasive cancer patients. Scale bar: 50 μm. **c & d)** The positivity (**C,** percentage of positively stained cells relative to the total number of cells in the tissue section) and intensity (**d**) of YAP1 immunosignals in the normal, CIN, and cancerous tissues. Data were analyzed using One-way ANOVA with Tukey's multiple comparisons post-test. Bars represent means ± SEM (n = 7 for normal tissues; n=28 for CIN I; n=23 for CIN II; n=29 for CIN III; n=68 for cervical cancer; *: $p < 0.05$; **: $p < 0.01$; ns: no significant difference).

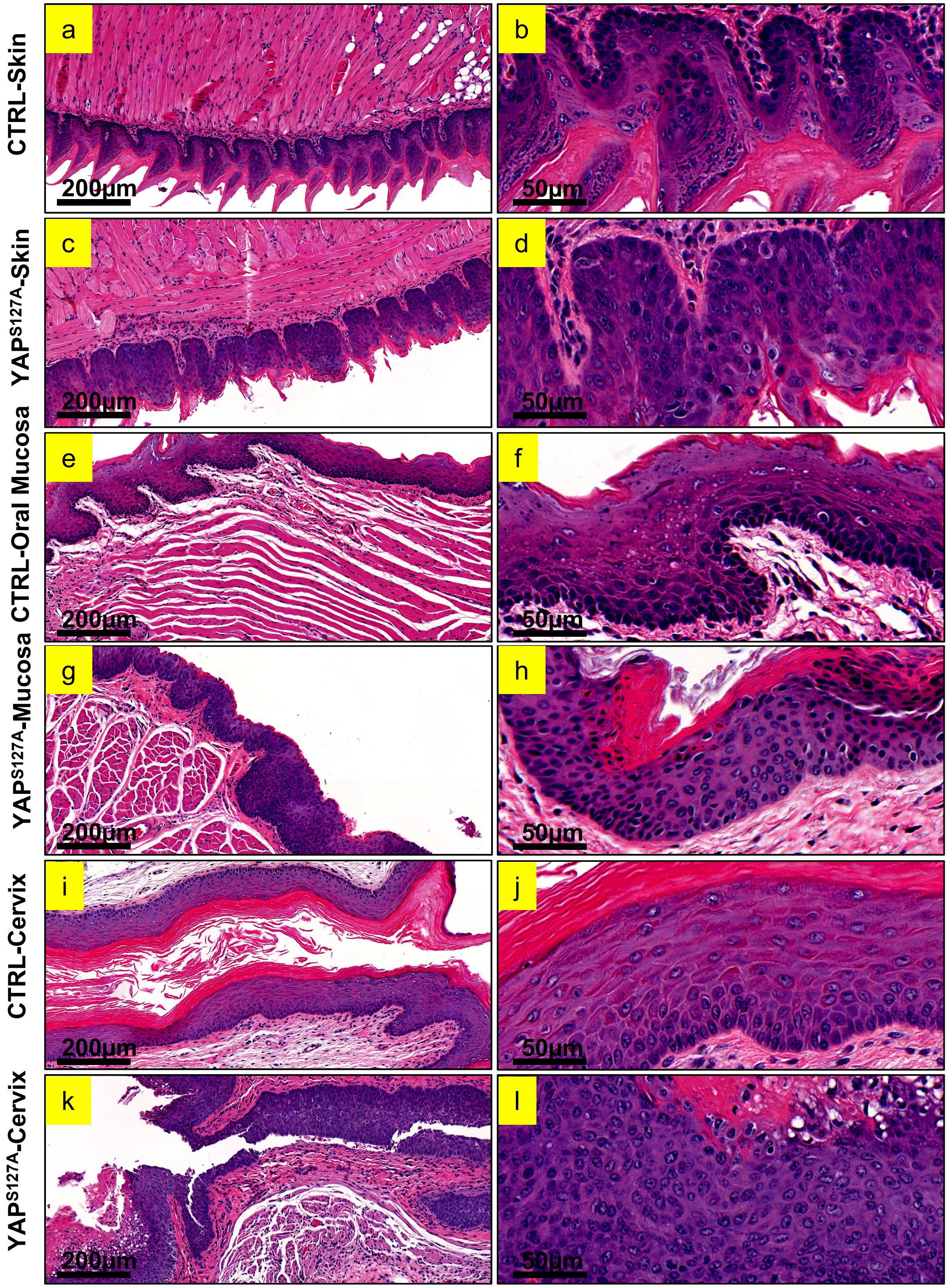

Figure S3. High concentration of Doxycycline induced epithelial neoplasia in KRT14-rtTA-TRE-YAP^{S127A} transgenic mice. Related to Figure 1. Representative images showing histology of epithelium in control and KRT14-^{rtTA}-$^{TRE-}$YAP^{S127A} transgenic mice treated with high concentration of doxycycline (2mg/ml in drink water). H&E staining showed that high concentration of Dox induced neoplasia not only in cervical epithelium, but also in epithelium of skin and oral mucosa of the KRT14-YAP^{S127A} transgenic mice.

He *et al.* Supplementary Figure S3

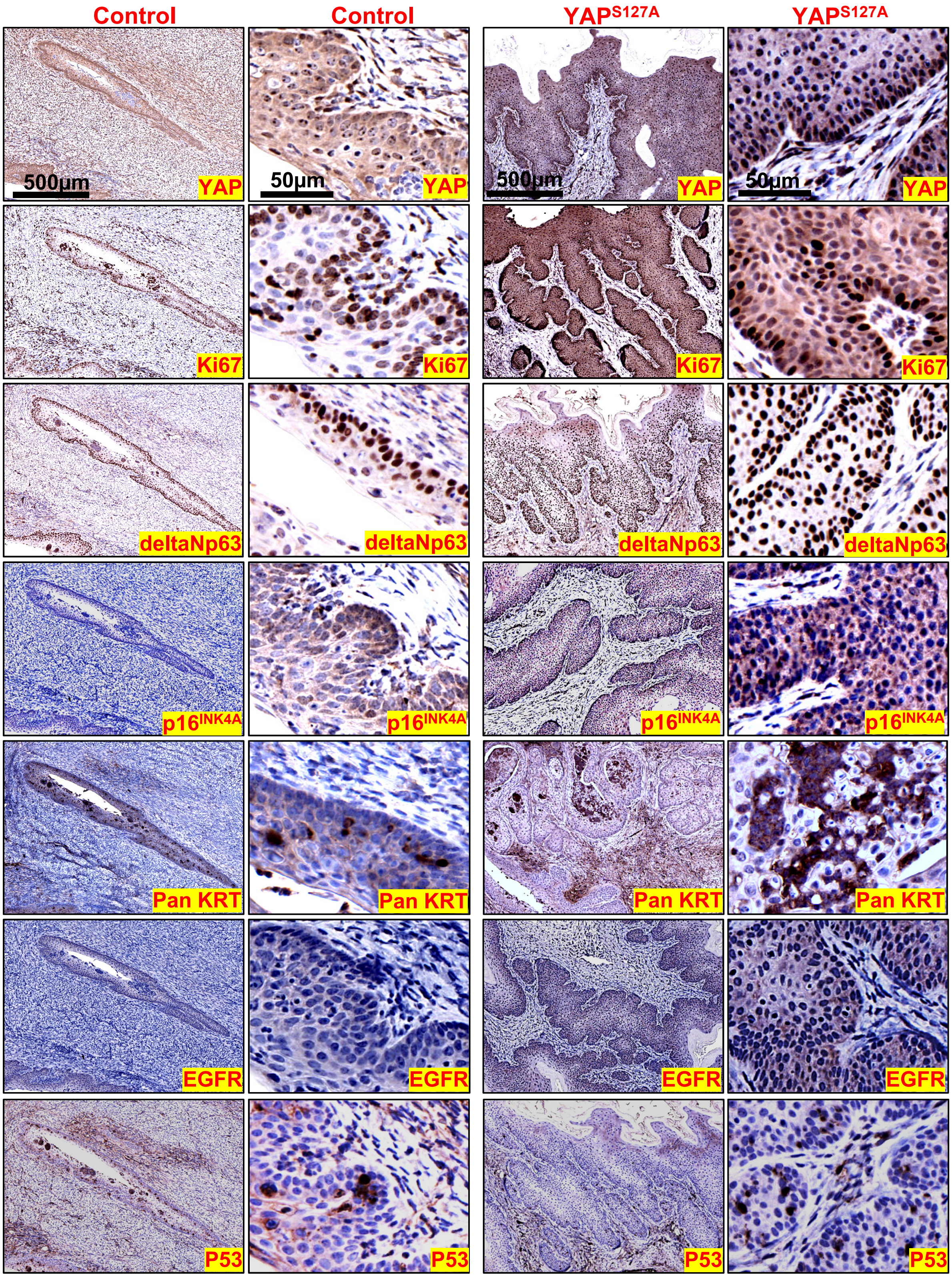

Figure S4. Molecular signature of Cervical Cancer derived from KRT14-YAP^{S127A} transgenic mice. Related to Figure 1. Representative images showing expressions of YAP1 and several known biomarkers (Ki67, deltaNp63, p16^{INK4A}, EGFR, and Pan KRT) for cervical squamous cell carcinoma (CVSCC) in tumor tissues derived from KRT14-YAP^{S127A} transgenic mice. Please note that all known markers are significantly accumulated in cervical tumor tissues derived from the KRT14-YAP^{S127A} transgenic mice. Please also note that p53 expression have no significant change. Scale bar: 500 µm in low magnification images; 50 µm in high resolution images.

He *et al.* Supplementary Figure S4

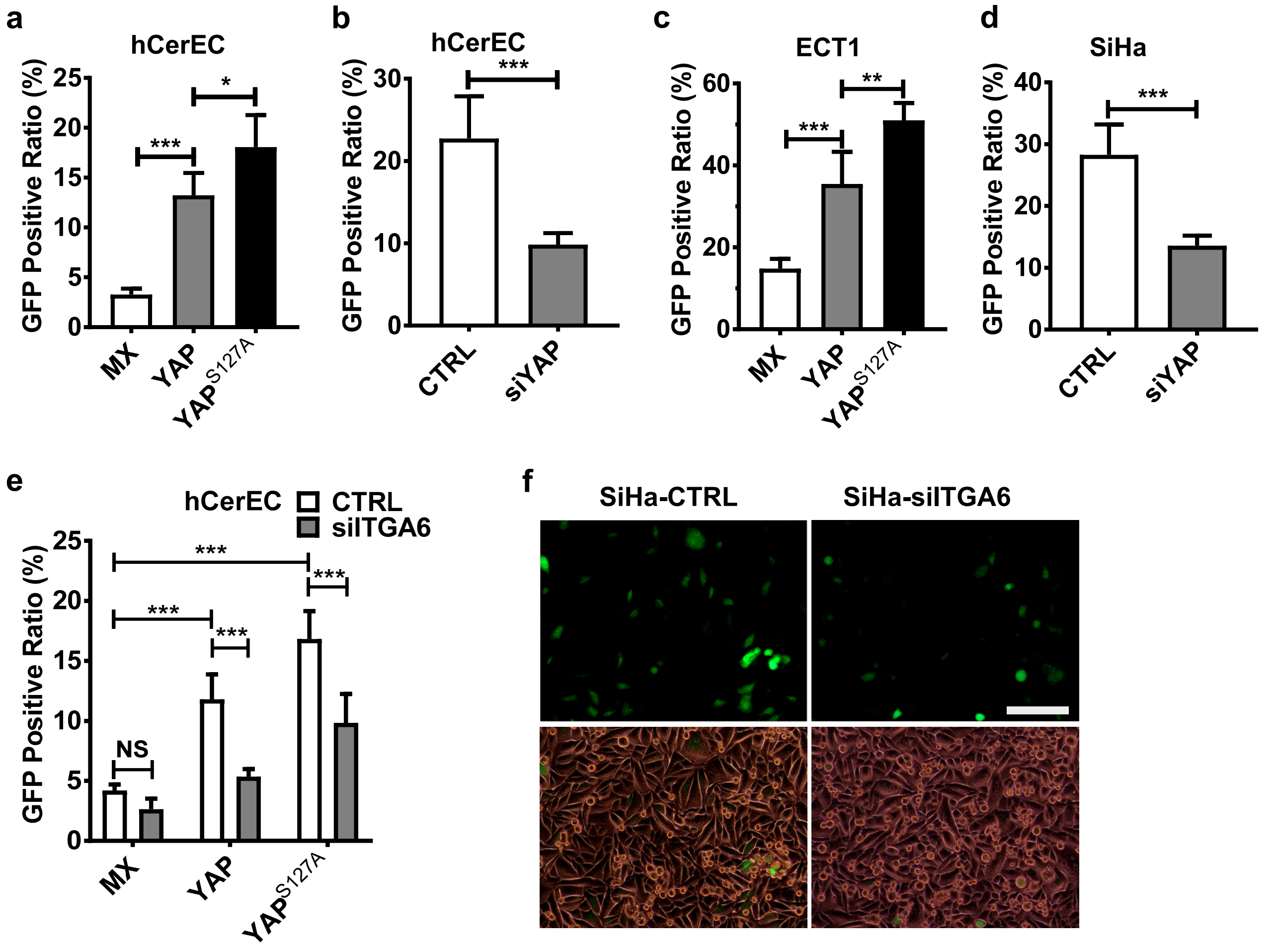

Figure S5. YAP1 regulates HPV infectivity via controlling the expression of the putative HPV receptors. Related to Figures 2 & 3. a) Quantitative data of Figure 2F showing the percentage of the GFP positive cells in hCerEc-MX (control), hCerEC-YAP, and hCerEC-YAP^{S127A} cells. **b)** Quantitative data of Figure 2G showing the percentage of the GFP positive cells in *YAP1*-knockdown hCerEC cells (siRNA, cells transfected with *YAP1* specific siRNA) and control hCerEC cells (CTRL, cells transfected with scramble siRNA). **c)** Quantitative data of Figure 2H showing the percentage of the GFP positive cells in ECT1-MX (control), ECT1-YAP, and ECT1-YAP^{S127A} cells. **d)** Quantitative data of Figure 2I showing the percentage of the GFP positive cells in YAP1-knockdown SiHa cells (siRNA, cells transfected with *YAP1* specific siRNA) and control SiHa cells (CTRL, cells transfected with scramble siRNA). Each bar represents the mean ± SEM (n = 5). *: $p < 0.05$; **: $p < 0.01$; ***: $p < 0.001$. Please note that HPV preferentially infects YAP1 highly-expressed cells. **e**) Quantitative results of figure 3C showing the ratio of GFP positive cells in hCerEC-MX, hCerEC-YAP, and hCerEC-YAP^{S127A} cells with (siITGA6) or without (CTRL) knockdown of ITGA6. Each bar represents mean ± SEM (n=5). NS, no significance; ***, $p < 0.001$. **f**) Representative images showing HPV16 PsV-derived GFP signal in SiHa-CTRL and SiHa-siITGA6 cells. Note that Knockdown of ITGA6 reduced HPV16 PsV-derived GFP signal in SiHa cells. Scale bar: 100 µm.

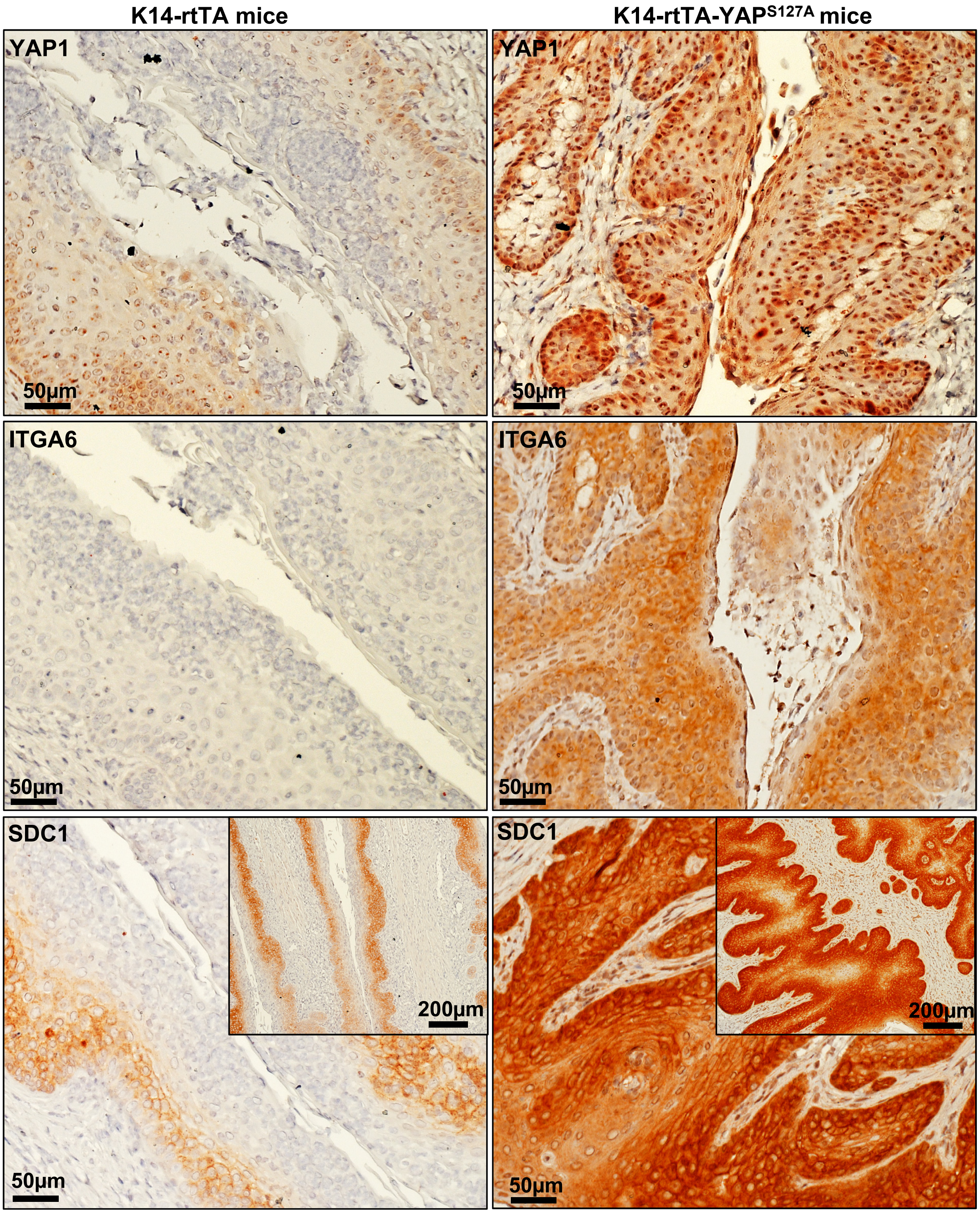

Figure S6. Upregulation of the putative HPV receptor molecules in cervical tissues of KRT14-YAP^{S127A} transgenic mice. Related to Figure 3. Representative images showing expressions of YAP1 and two well-studied putative HPV receptor molecules, SDC1 and ITGA6, in cervical tissues derived from 10-month old control (KRT-rtTA mice) and KRT14-YAP^{S127A} transgenic mice. Please note upregulation of SDC1 and ITGA6 proteins in the epithelium KRT14-YAP^{S127A} transgenic mice. Scale bar: 50µm.

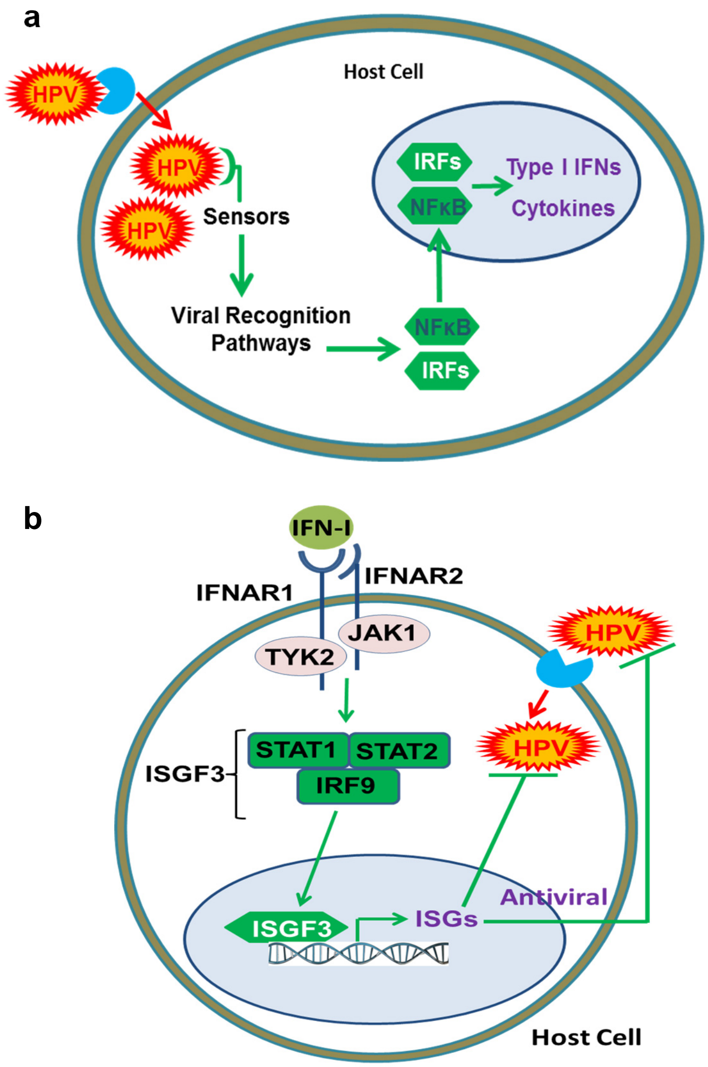

Figure 7. Schematic illustrations showing type I IFN-mediated antiviral signaling pathway. Related to Figures 4 and 5. a) Pathogen associated molecular patterns (PAMPs) of HPV are recognized by the pattern recognition receptors (PRRs) of HPV infected cells. The recognition of PAMPs initiates a series of signaling cascades that leads to the activation of interferon regulatory factor 3 (IRF3), which triggers expression of antiviral type I interferons (such as IFN-α and IFN-β). **b)** upon binding to their receptors, type I IFNs Activates JAK kinases and phosphorylate STATs. Phosphorylated STAT1 and STAT2 can bind to IRF9 to form a complex named 'IFN-Stimulated Gene Factor 3' (ISGF3), which translocates into the nucleus and initiates the transcription of a large spectrum of interferon-stimulated genes (ISGs) in virus infected cells as well as neighbor health cells. ISGs are involved in almost all key steps of antiviral effects in host cells, including inhibition of virus entry, blockade of virus replication, and obstruction of viral egress.

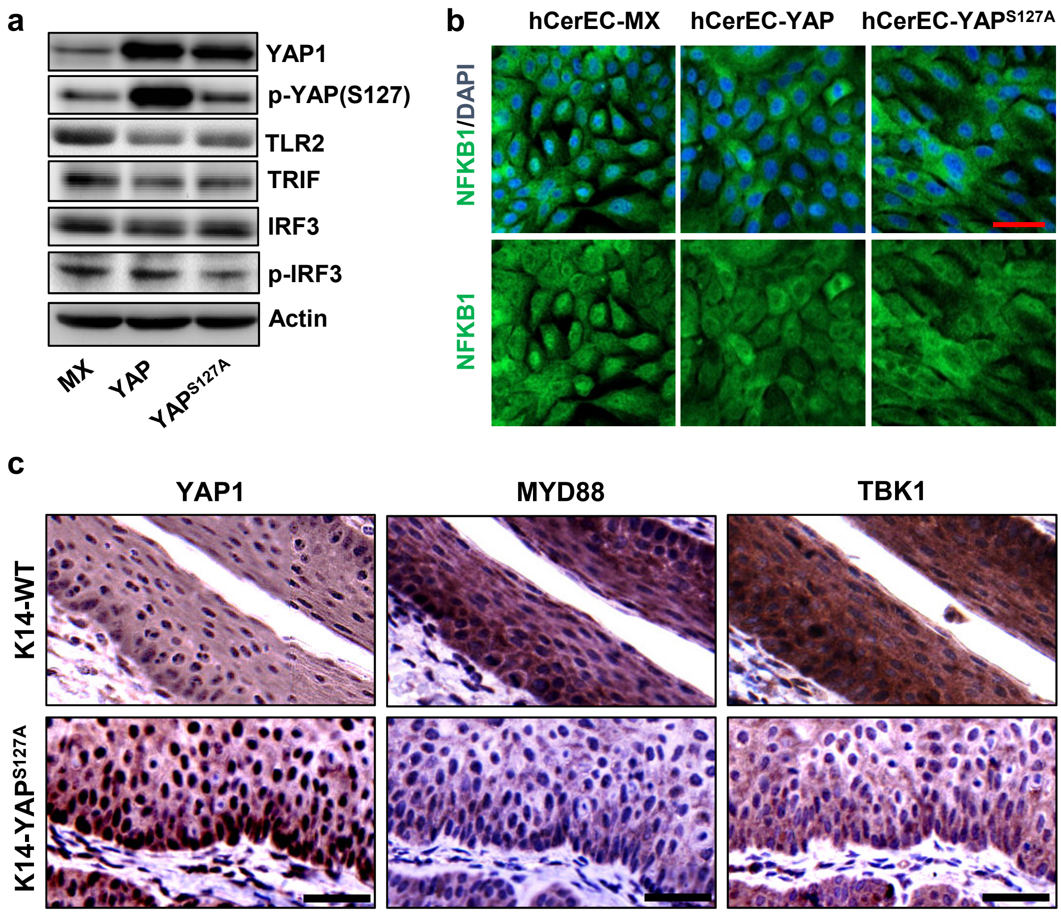

Figure S8. Hyperactivation of YAP1 in cervical epithelial cells inactivates signaling pathways involved in type I interferon production *in vitro* and *in vivo*. Related to Figures 4. a) Representative western blots from three independent experiments showing protein levels of YAP, TLR2, TRIF, and IRF3 in hCerEC-MX (control), hCerEC-YAP and hCerEC-YAP^{S127A} cells. **b**) Representative images showing the expressions and locations of NFκB1 in hCerEC-MX (control), hCerEC-YAP and hCerEC-YAP^{S127A} cells. NFκB1 proteins were visualized using an Alexa-488 (green) conjugated secondary antibody. Nuclei were stained with DAPI (blue). Scale bar: 50 µm. **c**) Representative high-resolution images showing expressions of MYD88 and TBK1, which are key components of the type I interferon production pathway, in cervical tissues of control and KRT14-YAP^{S127A} transgenic mice. Expression of proteins were detected by alkaline phosphatase-based detection systems using a VECTASTAIN® ABC-AP Staining Kit and antigens were visualized with a VECTASTAIN ELITE ABC kit (see details in Materials and Methods). The nuclei were counterstained with hematoxylin. Scale bar: **30** µm.

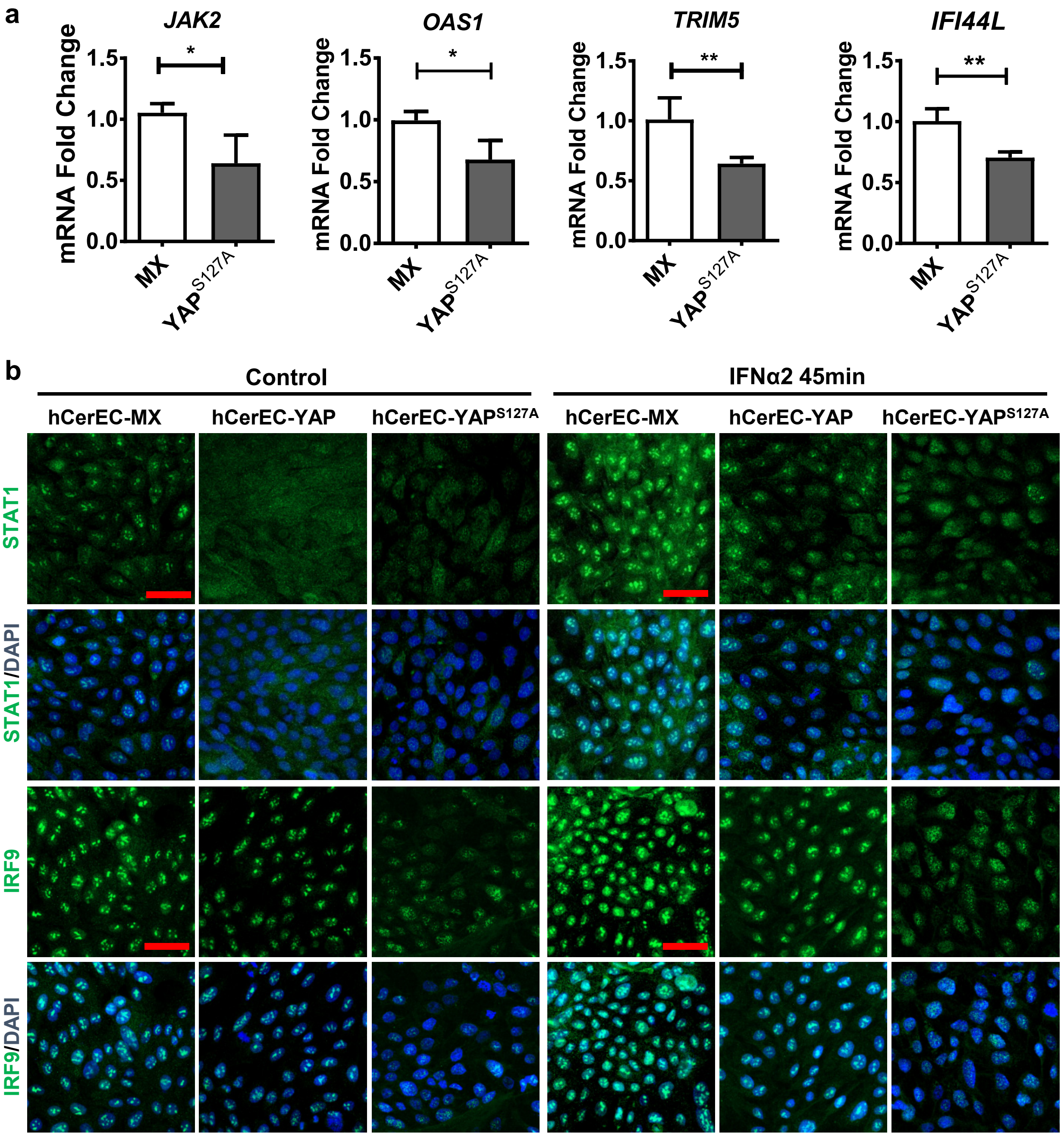

Figure S9. Hyper-activation of YAP1 suppresses the IFNs/JAKs/STATs signaling pathway in cervical epithelial cells. Related to Figure 5. a) Quantitative data showing mRNA levels of JAK2 and several interferon-stimulated genes (ISGs) in hCerEC-MX (control) and hCerEC-YAP^{S127A} cells. Each bar represents the mean ± SEM (n = 4). *: $p < 0.05$; **: $p < 0.01$; ***: $p < 0.001$. **b**) Representative images showing the expressions and locations of STAT1 and IRF9 in hCerEC-MX (control), hCerEC-YAP and hCerEC-YAP^{S127A} cells in the presence or absence of IFNα2b. STAT1 and IRF9 proteins were visualized using an Alexa-488 (green) conjugated secondary antibody. Nuclei were stained with DAPI. Scale bar: 20 μm.

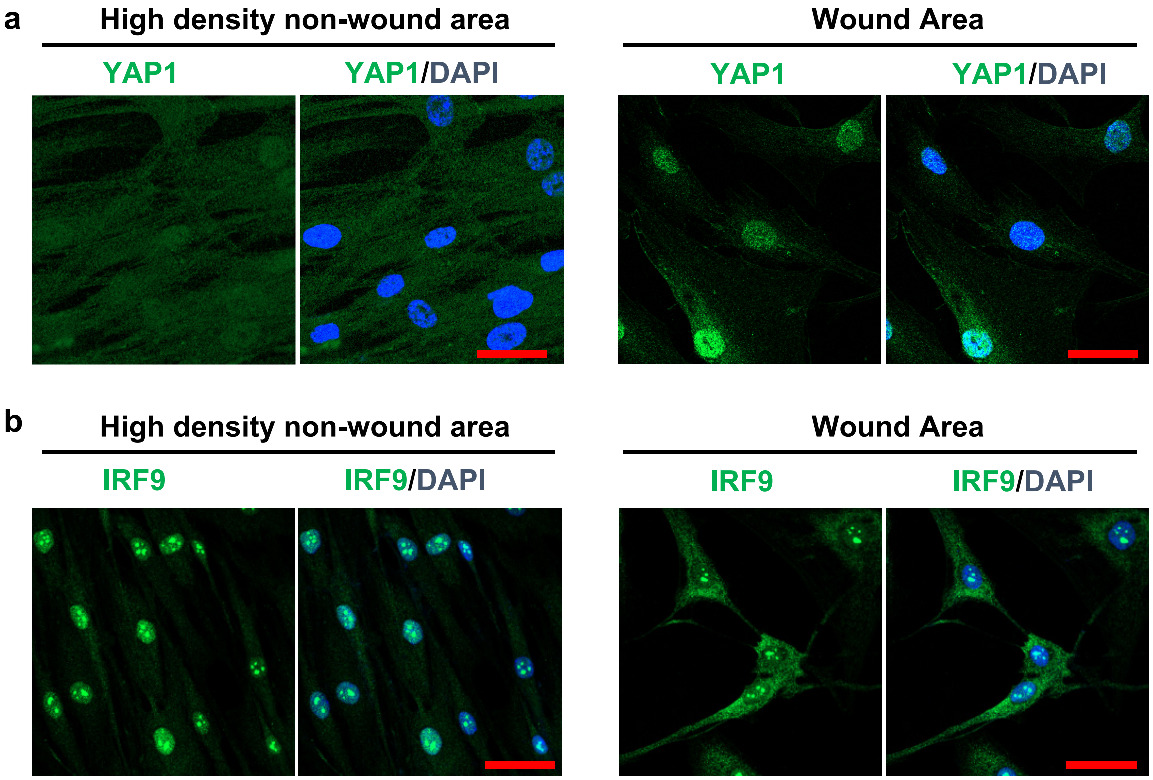

Figure S10. Hyperactivation of YAP1 in cervical epithelial cells inactivated signaling pathways involved in type I interferon production. Related to Figure 5. a) Representative images from three independent *in vitro* experiments showing expressions and locations of YAP1 in normal and "wound" area in cultured hCerEC cells. YAP1 protein were visualized using an Alexa-488 (green) conjugated secondary antibody. Nuclei were stained with DAPI (blue). Scale bar: 20µm. **b**) Representative images from three independent *in vitro* experiments showing expressions and locations of IRF9 in normal and "wound" area in cultured hCerEC cells. IRF9 protein were visualized using an Alexa-488 (green) conjugated secondary antibody. Nuclei were stained with DAPI (blue). Scale bar: 30µm.

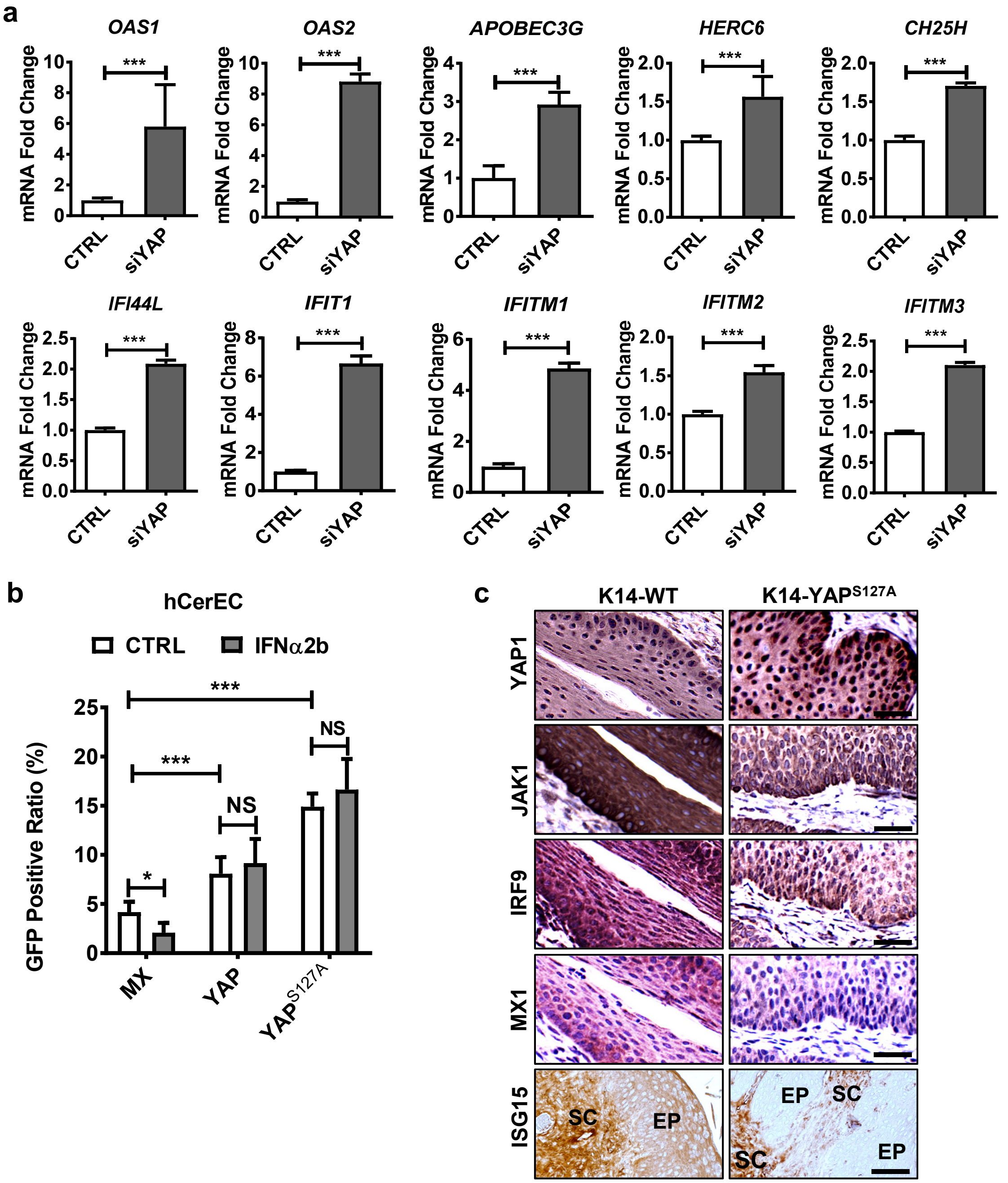

Figure S11. YAP1 suppresses the expression of key components of the JAKs-STATs pathway and antiviral ISGs in cervical epithelial cells *in vitro* and *in vivo*. Related to Figure 5. a) Quantitative data showing mRNA levels of several well-known antiviral ISGs. Each bar represents the mean ± SEM (n = 4). ***: $p < 0.001$. **b)** Quantitative data of figure **5D** showing the percentage of the GFP positive cells (HPV PsV infected cells) in hCerEC-MX (control), hCerEC-YAP, and hCerEC-YAP^{S127A} cells with or without pretreatment with IFNα2b. Each bar represents the mean ± SEM (n = 5). NS: no significant difference; *: $p < 0.05$; ***: $p < 0.001$. **c**) Representative high-resolution images showing YAP1-suppressed expressions JAK1 and IRF9, which are key components of the JAK1 - STATs signaling pathway, and MX1 and ISG15, which are well-studied downstream antiviral proteins of the JAK1-STATs signaling pathway, in cervical tissues of control and KRT14-YAP^{S127A} transgenic mice. Expression of proteins were detected by alkaline phosphatase-based detection systems using VECTASTAIN® ABC-AP Staining Kit and antigens were visualized with VECTASTAIN ELITE ABC kit (see details in Materials and Methods). The nuclei were counterstained with hematoxylin. SC: stroma cells; EP: Epithelial cells. Scale bar: 30 µm.

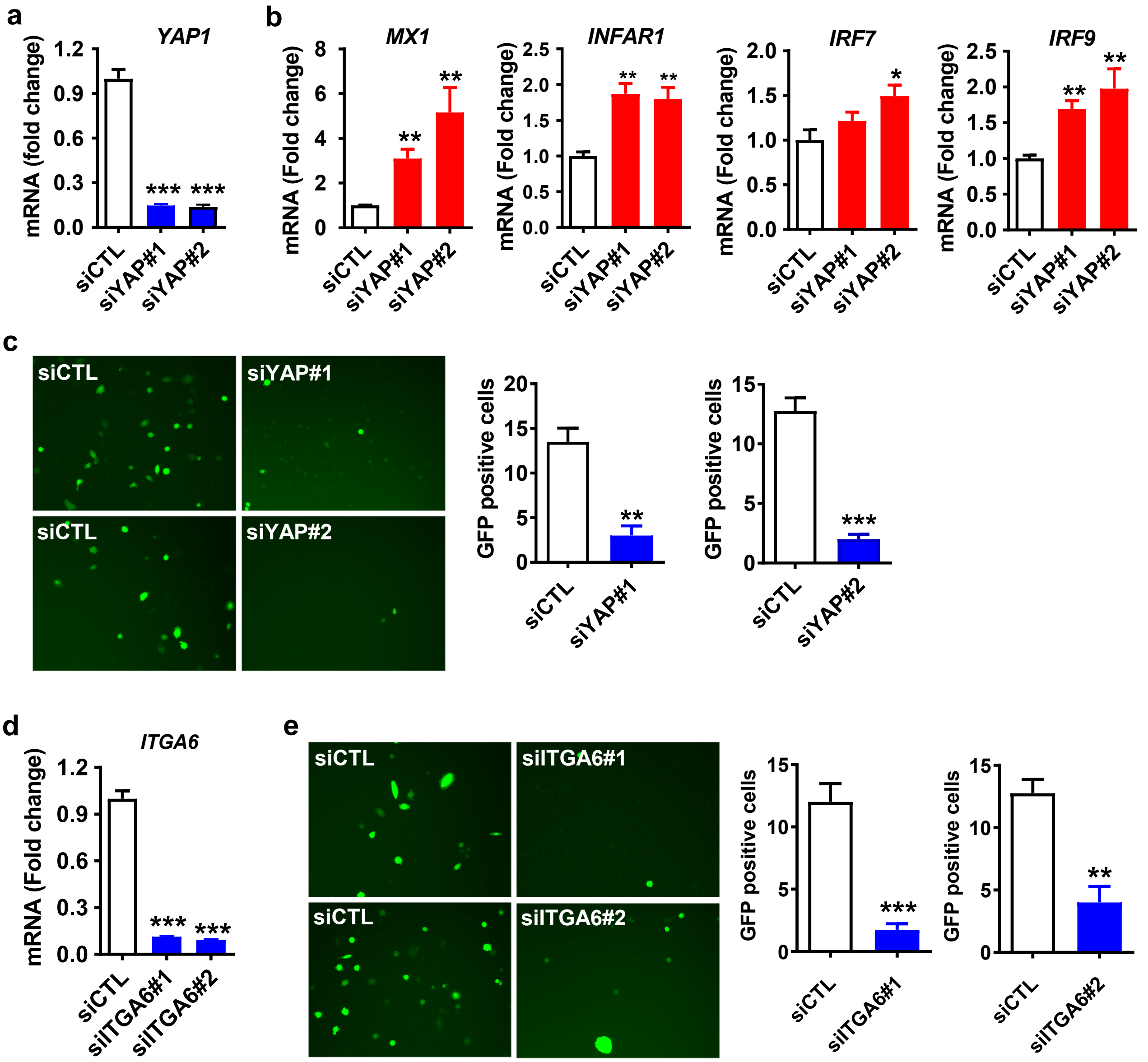

Figure S12. Validation of *YAP1* and *ITGA6* siRNAs. Related to STAR methods. a) Quantitative PCR results showing *YAP1* mRNA levels in SiHa cells transfected with control siRNA (siCTL) or *YAP1*-specific siRNAs (siYAP#1 and siYAP#2). Noting *YAP1* expression was significantly reduced by both siYAP#1 and siYAP#2. **b)** Quantitative PCR results showing relative mRNA levels of *MX1, INFAR1, IRF7,* and *IRF9* in SiHa cells transfected with control siRNA (siCTL) or *YAP1* targeting siRNAs (siYAP#1 and siYAP#2). *: $P < 0.05$; **: $P < 0.01$; ***: $P < 0.001$. **c**) Representative images showing HPV16 Pseudovirion (PsV)-derived GFP signal in SiHa cells transfected with siCTL or siYAP#1/siYAP#2. Bar graphs on the left showing GFP-positive cells ($n \geq 7$). Noting knockdown of *YAP1* significantly decreased HPV PsV infectivity. **: $P < 0.01$; ***: $P < 0.001$. **d**) Quantitative PCR results showing relative levels of *ITGA6* mRNA in SiHa cells transfected with control siRNA (siCTL) or *ITGA6* targeting siRNAs (siITGA6#1 and siITGA6#2). *ITGA6* mRNA level was significantly reduced by both siITGA6#1 and siITGA6#2. ***: $P < 0.001$. **e**) Representative images showing HPV16 PsV-derived GFP signal in SiHa cells transfected with siCTL or siITGA6#1/siITGA6#2. Bar graphs on the left showing quantitative results. Knockdown of *ITGA6* significantly decreased HPV PsV infection. **: $P < 0.01$; ***: $P < 0.001$.

Table S1. Primers used in this study*, Related to STAR methods (Mouse model studies & Quantitative Real Time-PCR*)*.

**Genotyping primers for KRT14-E6 / KRT14-E7 Mice.** To differentiate the E6 genotype from E7 genotype, the PCR products are digested with *HpaI*. Mice with E6 genotype will result in two PCR products that are of similar sizes, while mice with E7 genotype will result in a PCR product that is 50 nucleotides shorter.		
Primers	Sequences (5' to 3')	Product size
Forward	GGCGGATCCTTTTATGCACCAAAAGAGAACTG	800 bps
Reverse	CCCGGATCCTACCTGCAGGATCAGCCATG	
Internal control A	TATACTCAGAGCCGGCCT	250bps
Internal control B	ACAGCGTGGTGGTACCTTAT	
Internal control C	TCC TCG TGC TTT ACG GTA TC	
**Genotyping primers for KRT14-rtTA mice**		
Primers	Sequences (5' to 3')	Product size
Forward	CACGATACACCTGACTAGCTGGGTG	400 bps
Reverse	CATCACCCACAGGCTAGCGCCAACT	
Internal control A	CCATGTCTAGACTGGACAAGA	150bps
Internal control B	GTCAGTCGAGTGCACAGTTT	
**Genotyping primers for Tet-YAP^{S127A} mice**		
Primers	Sequences (5' to 3')	Product size
Forward Primer:	GACTCGGAGACCGACCTGGA	550 bps
Reverse Primer:	CATCATATTCTGCTGCACTGGTGGAC	
Internal control A	CCATGTCTAGACTGGACAAGA	150bps
Internal control B	GTCAGTCGAGTGCACAGTTT	
**Primers for qRT-PCR**		
Genes	Forward primer sequences	Reverse primer sequences
TLR2	GGCCAGCAAATTACCTGTGTG	CTGAGCCTCGTCCATGGGCCACTCC
TLR4	TGCAATGGATCAAGGACCAGAGGC	GTGCTGGGACACCACAACAATCACC
MyD88	CGGCAACTGGAGACACAAG	TCTGGAAGTCACATTCCTTGC
TRIF	GCAGCCCCGGATCCCT	TGTCCCCTACCCATTCACTGTT
IRF1	GCCAGTCGACGAGGATGAGGAAGGGAA	CCAGCGGCCGCCTGCTACGGTGCACAGG
IRF2	CCAGTCGACTACCTCAGCAACATGGGG	CCAGCGGCCGCGGCTTAACAGCTTGAC
IRF3	CGGAAGCTTCTGAAGCGGCTGTTGGTG	GTGCTCGAGACCATGAGGAGCGAGGGC
IRF4	CCAGTCGACGCAAGCTCTTTGACACAC	CCAGCGGCCGCCTTTTCATTCTTGAATAG
IRF5	GCCTTGTTATTGCATGCCAGC	AGACCAAGCTTTTCAGCCTGG
IRF7	TGCAAGGTGTACTGGGAG	TCAAGCTTCTGCTCCAGCTCCATAAG
IRF9	TTCTGTCCCTGGTGTAGAGCCT	TTTCAGGACACGATTATCACGG
NFkB1	CACTGCTCAGGTCCACTGTC	CTGTCACTATCCCGGAGTTCA
NFKB2	GGGGCATCAAACCTGAAGATTTCT	TCCGGAACACAATGGCATACTGT
IFNAR1	CAGCACCTGATGGCCTATCAC	TGGAGCATGAAGAACTGGATG
IFNAR2	CATCCACCTGAATGCCTACTTCT	TCTGCAAGGAGTCACCATTCTCT
STAT1	GGCACCAGAACGAATGAGGG	CCATCGTGCACATGGTGGAG
STAT2	GCAGCACAATTTGGGAA	ACAGGTGTTTCGAGAACTGGC
CH25H	ATCACCACATACGTGGGCTTT	GTCAGGGTGGATCTTGTAGCG
TRIM5	AGGAGTTAAATGTAGTGCT	ACCATGGATTTCTCATCTAT

IFI44L	GCTGCGGGCTGCAGAT	CTCTCTCAATTGCACCAGTTTCC
HERC6	CACTACCACTCCCTGGCATT	TGTTACTTCCCCAGCCAAAV
MX1	GTGCATTGCAGAAGGTCAGA	TCAGGAGCCAGCTTAGGTGT
ISG15	TCCTGCTGGTGGTGGACAA	TTGTTATTCCTCACCAGGATGCT
ISG20	ATCTCTGAGGGTCCCCAAGGA	TTCAGTCTGACACAGCCAGGCG
OAS1	TGATGCCCTGGGTCAGTTG	TCGGTGCACTCCTCGATGA
IFIT1	TCTCAGAGGAGCCTGGCTAA	CCAGACTATCCTTGACCTGATGA
IFITM1	GGATTTCGGCTTGTCCCGAG	CCATGTGGAAGGGAGGGCTC
IFITM2	ATTGTGCAAACCTTCTCTCCTG	ACCCCCAGCATAGCCACTTCCT
IFITM3	ACTGTCCAAACCTTCTTCTCTC	AGCACAGCCACCTCGTGCTC
IFITM5	TTGATCTGGTCGGTGTTCAG	GTCAGTCATAGTCCGCGTCA
IFNA	GTACTGCAGAATCTCTCCTTTCTCCTG	GTGTCTAGATCTGACAACCTCCCAGGCACA
IFNB	TTGTGCTTCTCCACTACAGC	CTGTAAGTCTGTTAATGAAG
YAP1	GCAACTCCAACCAGCAGCAACA	CGCAGCCTCTCCTTCTCCATCTG
ITGA6	ATTCAGGAGTAGCTTGGTGGAT	TTCTCTTGAAGAAGCCACACTTC
SDC1	GCTGACCTTCACACTCCCCA	CAAAGGTGAAGTCCTGCTCCC
EGFR	GAGGGTGCTCTTAGCCACAG	CATGGTGAGGGCTGAGGTGA
TBK1	CAACCTGGAAGCGGCAGAGTTA	ACCTGGAGATAATCTGCTGTCGA
JAK1	CTGTACGATGAGAGCACCAAGC	CGTGCCAGTTGGTAAAGTAGAACC
JAK2	TAAAGCACACAGAAACTATTCAGAGTC	AGAATATTCTCGTCTCCACAAAC
APOBEC3G	TCAGAGGACGGCATGAGACTTAC	AGCAGGACCCAGGTGTCATTG
RNA18S	CGCCGCTAGAGGTGAATTC	TTGGCAAATGCTTTCGCTC
GAPDH	GAGTCAACGGATTTGGTCGT	GACAAGCTTCCCGTTCTCAG

* Human JAK/STAT Signaling QT-PCR Primer Library (Item# HJAK-I) and Human Toll-like Receptor Signaling QT-PCR Primer Library (Item# HTLR-I) were purchased from Real Time Primers, LLC (Elkins Park, PA). Gene sets of these libraries could be obtained at: http://realtimeprimers.com/hujasiprli.html, and http://www.realtimeprimers.com/hutoresiprli.html.
