# Supplementary material for: A Human Papillomavirus-Independent Cervical Cancer Animal Model Reveals Unconventional Mechanisms of Cervical Carcinogenesis
Source: Cell Rep. Author manuscript; Available in PMC 2019 Oct 24. (PMC6812687; doi:10.1016/j.celrep.2019.02.004)
Supplement: 2 [file NIHMS1523267-supplement-2.pdf]

Cell Reports

Article

A Human Papillomavirus-Independent Cervical Cancer Animal Model Reveals Unconventional Mechanisms of Cervical Carcinogenesis

Graphical Abstract

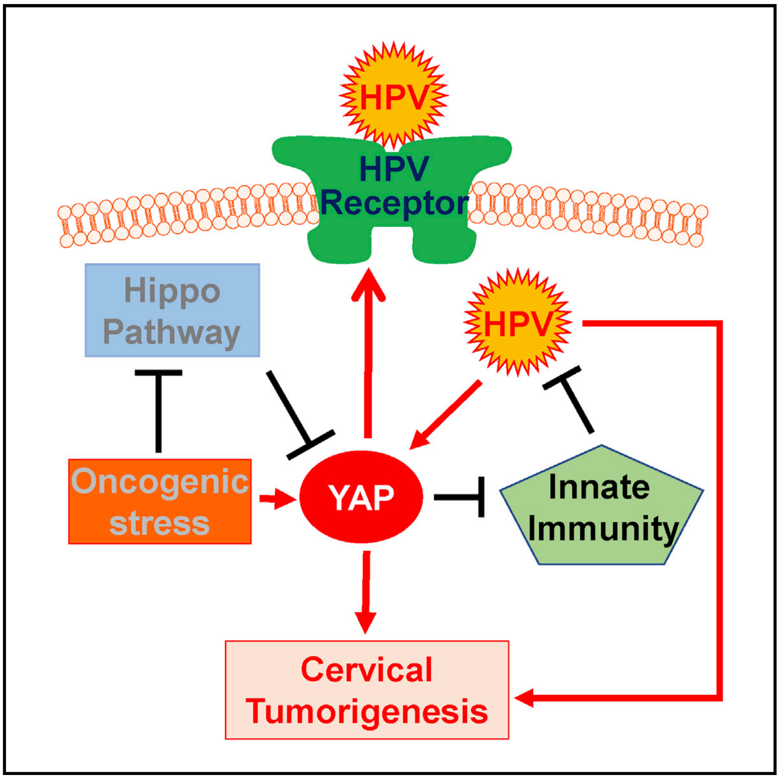

Highlights

- Human papillomavirus is not necessary for the development of cervical cancer
- Hyperactivated YAP1 in cervical epithelia is sufficient to drive cervical carcinogenesis
- Synergism between HPV and YAP1 accelerates cervical cancer initiation and progression
- Combined targeting of YAP1 and HPV may improve cervical cancer prevention and treatment

Authors

Chunbo He, Xiangmin Lv, Cong Huang, ..., Bo R. Rueda, John S. Davis, Cheng Wang

Correspondence

cwang34@mgh.harvard.edu

In Brief

HPV infections are common in healthy women and only rarely cause cervical cancer. He et al. provide evidence that hyperactivation of the YAP1 oncogene can drive cervical cancer initiation and progression. YAP1 hyperactivation in cervical epithelial cells increases the HPV receptors and disrupts host cell innate immunity, facilitating HPV infection.

He et al., 2019, Cell Reports *26*, 2636–2650
March 5, 2019 © 2019 The Author(s).
https://doi.org/10.1016/j.celrep.2019.02.004

CellPress

OPEN ACCESS
CellPress

Cell Reports
Article

A Human Papillomavirus-Independent Cervical Cancer Animal Model Reveals Unconventional Mechanisms of Cervical Carcinogenesis

Chunbo He,[1,2,3,10] Xiangmin Lv,[1,2,10] Cong Huang,[1] Peter C. Angeletti,[4] Guohua Hua,[2,3] Jixin Dong,[5] Jin Zhou,[2,6] Zhengfeng Wang,[2,7] Bowen Ma,[2] Xingcheng Chen,[5] Paul F. Lambert,[8] Bo R. Rueda,[1] John S. Davis,[2,9] and Cheng Wang[1,2,11,*]

[1]Vincent Center for Reproductive Biology, Vincent Department of Obstetrics and Gynecology, Massachusetts General Hospital, Boston, MA 02114, USA
[2]Olson Center for Women's Health, Department of Obstetrics & Gynecology, University of Nebraska Medical Center, Omaha, NE 68198, USA
[3]College of Animal Science and Technology, Huazhong Agricultural University, Wuhan, 430070, China
[4]Nebraska Center for Virology, School of Biological Sciences, University of Nebraska-Lincoln, Lincoln, NE 68583, USA
[5]Fred & Pamela Cancer Center, University of Nebraska Medical Center, Omaha NE 68198, USA
[6]Department of Obstetrics and Gynecology, The Eighth Affiliated Hospital of Sun Yat-Sen University, Shenzhen, 518033, China
[7]Department of Hepatobiliary Surgery, The First Affiliated Hospital of Zhengzhou University, Zhengzhou, 45001 China
[8]McArdle Laboratory for Cancer Research, Department of Oncology, University of Wisconsin-Madison, Madison, WI 53705, USA
[9]Omaha Veterans Affairs Medical Center, Omaha, NE 68105, USA
[10]These authors contributed equally
[11]Lead Contact
*Correspondence: cwang34@mgh.harvard.edu
https://doi.org/10.1016/j.celrep.2019.02.004

SUMMARY

HPV infections are common in healthy women and only rarely cause cervical cancer, suggesting that individual genetic susceptibility may play a critical role in the establishment of persistent HPV infection and the development of cervical cancer. Here, we provide convincing *in vitro* and *in vivo* evidence showing that differential expression and activation of *YAP1* oncogene determine individual susceptibility to HPV infection and cervical carcinogenesis. We found that hyperactivation of YAP1 in mouse cervical epithelium was sufficient to induce invasive cervical cancer. Cervical epithelial cell-specific HPV16 E6/E7 and *YAP1* double-knockin mouse model demonstrated that high-risk HPV synergized with hyperactivated YAP1 to promote the initiation and progression of cervical cancer. Our mechanistic studies indicated that hyperactivation of YAP1 in cervical epithelial cells facilitated HPV infection by increasing the putative HPV receptor molecules and disrupting host cell innate immunity. Our finding reveals an unconventional mechanism for cervical carcinogenesis.

INTRODUCTION

Cervical cancer is the most common gynecologic cancer and the fourth leading cause of cancer-related death in women worldwide. According to the latest statistics of the International Agency for Research on Cancer (IARC), approximately 527,000 women are diagnosed with cervical cancer, and an estimated 265,000 women die of this disease each year, equal to almost one-tenth of global cancer deaths in women (Torre et al., 2015). The increased use of the Pap test and intensive implementation of health education programs have reduced cervical cancer death by more than 50% in the United States over the past 40 years. Nevertheless, the National Cancer Institute estimates that ~250,000 women are living with cervical cancer in the United States (Howlader et al., 2018). The treatment for cervical cancer includes surgery, radiation, or concurrent chemoradiation. However, the survival rates of advanced-stage and recurrent cervical cancer patients are still very low. Cervical cancers of advanced stages and distant recurrence are currently considered to be incurable. Effective new therapeutic options are currently not available, because the detailed mechanism or mechanisms underlying cervical cancer development and progression are unclear at present.

Because the majority of cervical cancer patients are positive for human papillomavirus (HPV) DNA, it is widely accepted that all cases of cervical cancer are caused by high-risk HPV (Walboomers et al., 1999). However, although the lifetime risk for HPV infection is more than 75% (Koutsky, 1997), only a very small portion of women infected with HPV develop cancers (Kulasingam et al., 2002), suggesting that HPV alone is not sufficient for the malignant transformation of cervical epithelial cells. The molecular mechanism or mechanisms underlying the tumorigenesis of cervical epithelium are unclear.

The Hippo pathway is an evolutionarily conserved pathway that controls organ development from the fruit fly to mammals (Dong et al., 2007; Mo et al., 2014). Overall, the upstream core kinases of the Hippo pathway, including FAT1/2/3/4, Nf2, Mst1/2, Sav1, LATS1/2, and MOB1a/b, have been identified as tumor suppressors, while the downstream effectors, mainly YAP1 and TAZ (WWTR1) transcriptional co-activators, have been described as oncoproteins (Pan, 2010; Zanconato et al., 2016). Our previous studies showed that the Hippo/YAP

 © 2019 The Author(s).
This is an open access article under the CC BY license (http://creativecommons.org/licenses/by/4.0/).

OPEN
ACCESS
CellPress

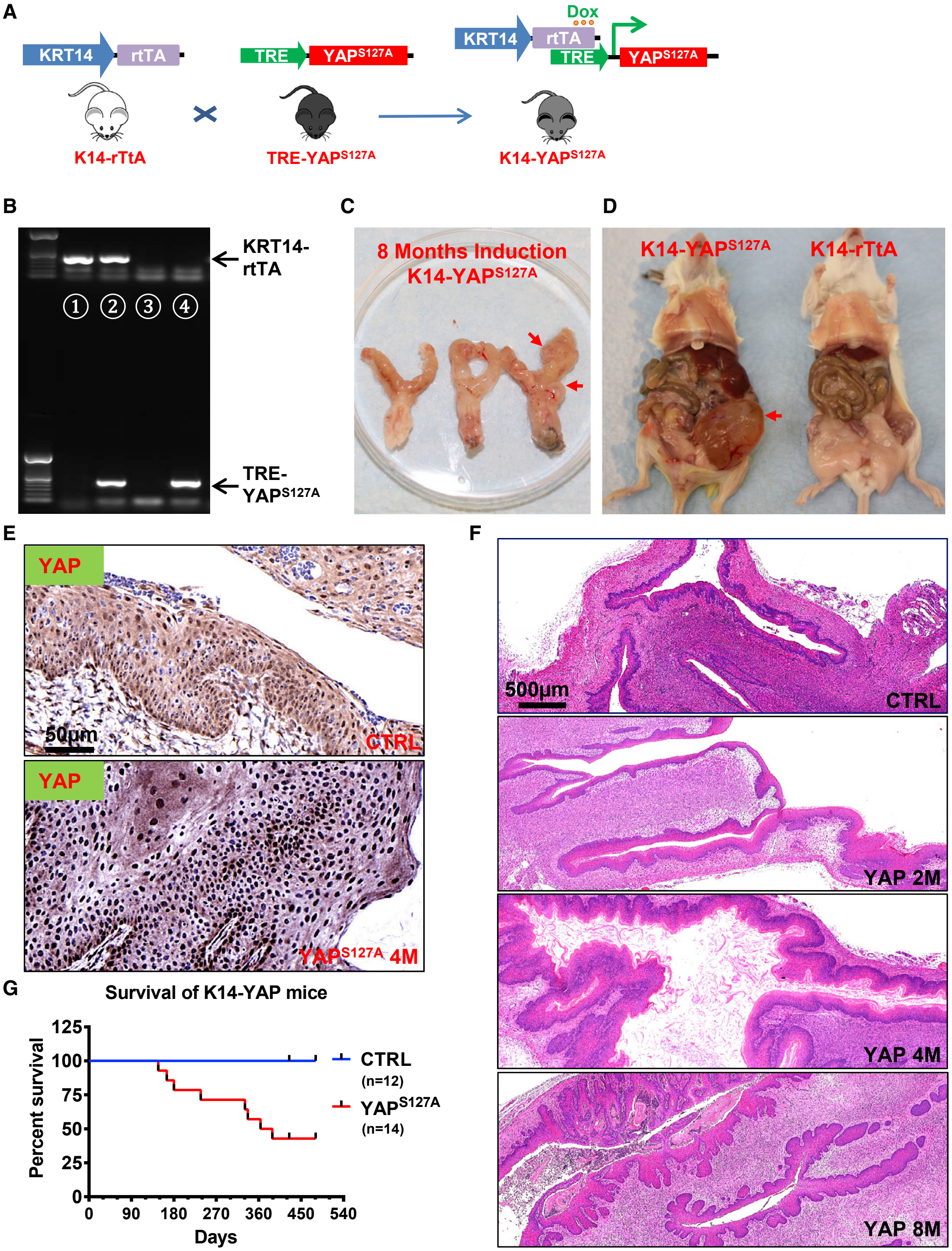

Figure 1. Hyperactivation of YAP1 Induces Invasive Cervical Cancer in Mice

(A) A schematic diagram showing the transgenic mouse strains and breeding procedures for generating KRT14-YAP^{S127A} mice (mice with keratin 14 [KRT14]-driven and tetracycline-induced expression of YAP^{S127A} gene in cervical epithelium).

(B) Representative gel image showing PCR products (genotyping results) of wild-type (lane 3), KRT14-rtTA alone (lane 1), TRE-YAP alone (lane 4), and KRT14-rtTA and TRE- YAP^{S127A} co-expressed (lane 2) mice.

(C) Representative image showing the reproductive tract from KRT14-YAP^{S127A} transgenic mice after induction of gene expression with doxycycline (0.05 mg/mL) for 8 months. Note the invasion of cancer in the uterine and vaginal area (red arrow).

(legend continued on next page)

signaling pathway may play a role in cervical carcinogenesis. We found that YAP1, the major effector of the Hippo signaling pathway, interacted with HPV16 E6 oncoprotein to drive the initiation and progression of cervical cancer (He et al., 2015b). Very recently, The Cancer Genome Atlas (TCGA) Research Network published the most comprehensive cervical cancer genome, analyzing results after sequencing tissues derived from 228 primary cervical cancers (Cancer Genome Atlas Research Network et al., 2017). The extended TCGA dataset indicated that a gene cluster with high copy number alterations mostly contained squamous tumors with amplification events involving 11q22 (*YAP1*, *BIRC2*, and *BIRC3*) and 7p11.2 (*EGFR*). These data strongly support a role of the Hippo/YAP1 pathway in the development of cervical cancer. In the present study, we successfully developed several transgenic mouse models indicating that the Hippo/YAP1 pathway is at the center of cervical carcinogenesis. We found that hyperactivation of YAP1 in the cervical epithelial cells is sufficient to induce cervical squamous cell carcinoma (CVSCC). We also found that hyperactivated YAP1, by upregulating expression of the putative HPV receptors and suppressing innate immunity, facilitates HPV infection of cervical epithelial cells. HPV in turn synergizes with YAP1 to promote the initiation and progression of cervical cancer.

RESULTS

The Hippo/YAP Pathway Is Frequently Dysregulated in Cervical Cancer Patients

To examine the role of the Hippo/YAP pathway in the development of cervical cancer, we first analyzed the genomic data of 191 cervical cancer patients, which were deposited in TCGA (Cancer Genome Atlas Research Network et al., 2017; Cerami et al., 2012). The results showed that YAP1, the oncogenic effector of the Hippo pathway, was frequently (11%) amplified, while LATS1/2, MST1, and FATs, which are upstream tumor suppressors of the Hippo pathway, were frequently deleted or mutated in cervical cancer patients (Figure S1A). Importantly, we found that the rates of overall survival and disease-free survival of 74 patients with amplification of YAP1 or deletion and/or mutation of tumor suppressors (FAT1/2/3/4, MST1/2, LATS1/2) were significantly poorer than in 96 patients without genomic alterations (Figures S1B and S1C), suggesting that dysregulation of the Hippo/YAP pathway in patients is associated with poor outcomes. Consistent with these results, immunohistochemistry (IHC) studies showed that both the immunosignal intensity and positivity of YAP1 protein significantly increased with grades of cervical intraepithelial neoplasia (CIN) (n = 7 for normal control, n = 28 for CIN1, n = 23 for CIN2, n = 29 for CIN3; Figures S2A–S2D). Cervical cancer tissues from advanced-stage patients had higher expression levels and more nuclear accumulation of YAP1 protein compared with those from early-stage patients (n = 68; Figures S2A–2D), indicating that the activity of YAP1 was elevated with cancer progression.

Hyperactivation of YAP1 Is Sufficient to Induce Cervical Cancer *In Vivo*

To examine whether hyperactivation of YAP1 plays a role in cervical carcinogenesis, we generated a mouse model with cervical epithelial cell-specific expression of YAP^{S127A} (Figure 1A). In these transgenic mice (hereafter referred to as KRT14-YAP^{S127A}), the expression of YAP^{S127A} (constitutively active YAP1) was under the control of the keratin 14 (KRT14) promoter and doxycycline (Dox) availability, allowing inducible expression of YAP^{S127A} in mouse cervical epithelial cells (Figure 1A). After PCR-based genotyping (Figure 1B), Dox-containing water was used to replace the normal drinking water of 3-month-old control (KRT14-rtTA or TRE-YAP^{S127A} alone) and KRT14-YAP^{S127A} mice. We found that high concentration of DOX (2.0 mg/mL) induced hyperplasia of squamous epithelium in multiple organs within 2 weeks, leading to rapid death of these animals (Figure S3). However, when the concentration of Dox in drinking water was reduced to 0.05 mg/mL, multi-organ hyperplasia was significantly alleviated, but ectopic YAP^{S127A} was still expressed in CVSCCs (Figure 1E). As shown in Figure 1F, low concentration of Dox-induced expression of YAP^{S127A} resulted in drastic hyperplasia of cervical epithelial cells within 2 months in KRT14-YAP^{S127A} mice but not in control mice. Most of KRT14-YAP^{S127A} mice developed invasive cervical tumors after induction with 0.05 mg/ML Dox for 6–8 months (Figures 1C, 1D, and 1F). With the progression of cervical cancer, ureteral obstruction, which is frequently present in advanced human cervical cancer patients, was observed in some KRT14-YAP^{S127A} mice (Figure 1D). About 50% of KRT14-YAP^{S127A} mice died after 13 months of induction of YAP^{S127A} expression with Dox (Figure 1G). IHC analysis indicated that known protein biomarkers currently available for cervical cancer subtype screening (Ki67, $P16^{INK4A}$, deltaNp63, EGFR, and Pan KRT) were highly expressed in cervical cancer tissues from the KRT14-YAP^{S127A} mice (Figure S4). No obvious difference was observed in the TP53 protein levels between the control and cancerous cervical tissues (Figure S4). These biochemical features support the pathological conclusion that cancers in KRT14-YAP^{S127A} mice are CVSCC.

YAP1 Facilitates HPV Infection of Cervical Epithelial Cells

Although our animal model showed that hyperactivation of YAP1 is sufficient to induce the development of CVSCC, the strong epidemiological association between HPV infection and cervical

(D) A representative image showing the general anatomy of KRT14-rtTA control mice and KRT14-YAP^{S127A} mice. Note the obstruction of the urination system (the red arrow points to an enlarged bladder) caused by ureter blockage due to cervical tumor in KRT14-YAP^{S127A} transgenic mice.
(E) Representative images showing expression of YAP1 protein in cervical tissues of control and KRT14-YAP^{S127A} transgenic mice.
(F) Representative images showing H&E staining of cervical tissues form control and KRT14-YAP^{S127A} transgenic mice. Note the cancer progression in the cervical epithelium after doxycycline induction for 8 months. Scale bar: 500 μm.
(G) Kaplan-Meier curve showing survival rates of control mice (blue line, n = 12) and KRT14-YAP^{S127A} mice (red line, n = 14) after doxycycline induction of YAP1 expression in cervical epithelial cells.

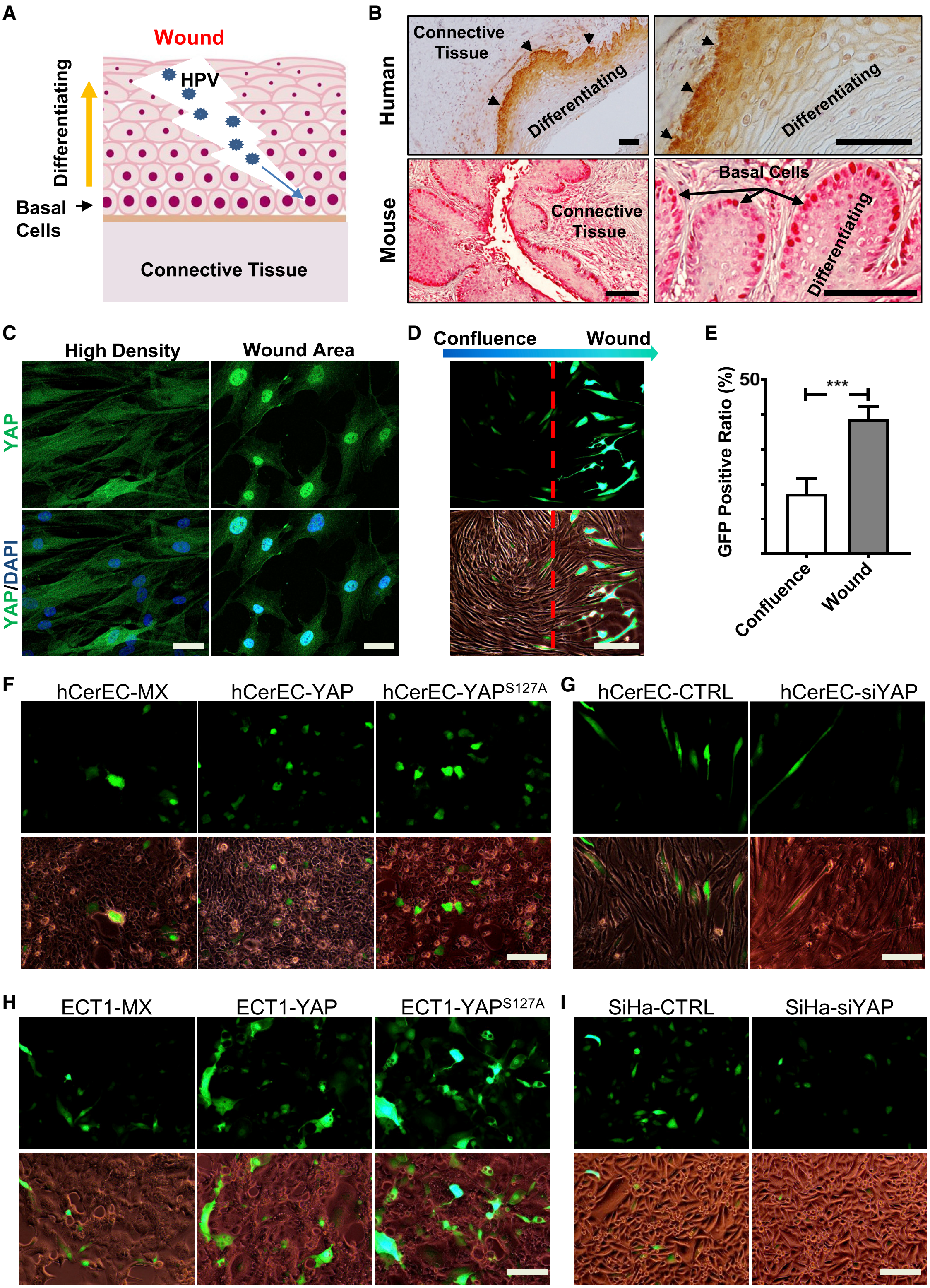

Figure 2. Hyperactivation of YAP1 Increases Susceptibility of Human Cervical Epithelial Cells to HPV Infection

(A) A schematic cartoon showing the natural process of HPV infection of human cervix. HPV infects basal epithelial cells when a wound is created in cervical epithelium.

(legend continued on next page)

cancer implies that HPV oncoproteins are involved in cervical tumorigenesis. HPV primarily infects basal epithelial cells when a wound is created in cervical tissue (Figure 2A). IHC showed that YAP1 protein was highly expressed in cervical basal cells in human and mouse cervix (Figure 2B). This coincidence suggests that high YAP1 in the basal cells may facilitate HPV infection during wound healing process. Indeed, a wound-healing assay using primary cultures of human cervical epithelial cells (hCerECs) showed that in the "wound" area, YAP1 was predominately localized to nuclei (active form of YAP1), while in the non-wound area, YAP1 protein was almost evenly distributed in cytoplasm and nucleus of hCerECs (Figure 2C). We then used a HPV16 pseudovirion (HPV16 PsV) (Roberts et al., 2007) to examine if YAP1 expression affected HPV infectivity. After incubating hCerECs with HPV16 PsVs (MOI = 2.0) for 72 h, GFP-positive cells were detected in the wound area (Figure 2D), but few GFP-positive cells were found in the non-wound area (Figures 2D and 2E). The signal intensity of GFP was also much stronger in hCerECs in the wound area (Figure 2D). Incubating hCerECs with HPV16 PsVs (MOI = 1.0) for 72 h in the regular two-dimensional (2D) culture results in more GFP-positive cells in hCerEC-YAP and hCerEC-YAP^{S127A} cells compared with hCerEC-MX control cells (Figures 2F and S5A). In contrast, knockdown of YAP1 significantly reduced the infectivity of HPV16 PsV in hCerECs after incubating with HPV16 PsV (MOI = 2.0) for 72 h (Figures 2G and S5B). Similar results were observed in YAP1 differentially expressed ECT1 cells and SiHa cells (Figures 2H, 2I, S5C, and S5D). These results provide direct evidence that hyperactivation of YAP1 increases the susceptibility of cervical epithelial cells to HPV infection.

YAP1 Facilitates HPV Infection of hCerEC Cells by Upregulating the Putative HPV Receptors

The specificity and susceptibility of a host cell to viral infection are controlled by the interaction between virus and the putative viral receptor molecules on the host cell membrane (Grove and Marsh, 2011). Heparan sulfate proteoglycans (HSPGs; such as Syndecan-1), integrin α6 (ITGA6), and epidermal growth factor receptor (EGFR) have been identified as putative HPV receptor molecules (Raff et al., 2013; Surviladze et al., 2012; Yoon et al., 2001). Interestingly, we found that all examined putative HPV receptor molecules were significantly upregulated in YAP1 hyperactivated hCerECs but downregulated in YAP1-knockdown hCerECs (Figures 3A and 3B). Knockdown of ITGA6 in hCerEC-YAP and hCerEC-YAP^{S127A} cells blocked YAP- and YAP^{S127A}-induced increase of HPV16 PsV intake (indicated by GFP signal) in these cells after incubation with HPV16 PsV (MOI = 1.0) for 72 h (Figures 3C and S5E). Similarly, knockdown of *ITGA6* in SiHa cervical cancer cells also inhibited the efficiency of HPV16 PsV infection (MOI = 1.0) (Figure S5F). Importantly, we found that hyperactivation of YAP1 also upregulated expression of ITGA6, SDC1, and EGFR *in vivo* (Figures 3D, S4, and S6). IHC analyses showed that expression of ITGA6 and SDC1 in the epithelium of the KRT14-YAP^{S127A} mice is significantly increased compared with that of the KRT14-rtTA control mice (Figure S6). These results demonstrate that hyperactivation of YAP1 upregulates the expression of the putative HPV receptors on the membrane of cervical epithelial cells to facilitates HPV infection.

Hyperactivation of YAP1 Interrupts Type I Interferon Production in Cervical Epithelial Cells

Under normal conditions, HPV infection is thought to be cleared up by the immune system. However, under certain pathological conditions, host cell immunity may be compromised, and HPV escapes immune surveillance, leading to establishment of persistent infection and increased risk for cervical cancer in these patients. The innate immune system constitutes the first line of defense against HPV infection (Amador-Molina et al., 2013). Fluorescent immunocytochemistry in the wound healing model showed that IRF3, one of the major transcription factors controlling production of type I interferons (Figure S7A), is negatively associated with YAP1 activity (Figure 4A). In non-wound areas of the wound-healing assay, inactive YAP1 was mainly localized to cytoplasm of hCerECs, while IRF3 was localized mainly to nuclei (Figure 4A). However, in the "wound" area, YAP1 was predominantly translocated to nuclei (activated), while IRF3 was localized predominantly to cytoplasm (inactive form; Figure 4A). These results suggest that YAP1 may manipulate IRF3 activity to modulate innate immunity in cervical epithelial cells.

(B) Representative images showing the expression of YAP1 (red) in mouse and human normal cervical tissues. Note that YAP1 is expressed mainly in basal cells (arrow and arrowhead) and localized predominantly to the nuclei of these cells in cervical epithelium. Scale bar: 100 μm.

(C) Representative images showing cellular localization of YAP1 protein (green) in hCerECs during wound healing. *In vitro* wound-healing assay showed that in confluent area, YAP1 was localized predominantly in the cytoplasm of hCerECs, while in the "wound" area, YAP1 translocated into the nuclei of these cells. Scale bar: 20 μm.

(D) Representative images showing that HPV preferentially infected hCerECs in "wound" area. hCerECs were incubated in growth media with HPV16 pseudovirions (PsVs; MOI = 2) for 72 h. The infection efficiency of HPV16 PsV in hCerECs is represented by the ratio of GFP-positive cells. Note that the positivity and intensity of GFP signal were localized predominantly to cells in "wound healing" area. Scale bar: 100 μm.

(E) Quantification results of (D). Each bar represents mean ± SEM (n = 5). ***$p < 0.001$.

(F) Representative images showing HPV16 PsV-derived GFP signal in hCerEC-MX cells (hCerECs transfected with an empty vector as control), hCerEC-YAP cells (hCerECs transfected with a vector expressing wild-type YAP1), and hCerEC-YAP^{S127A} cells (hCerECs transfected with a vector expressing YAP^{S127A}, a constitutively active form of YAP1). Cells were incubated in the growth media with or without HPV16 PsV (MOI = 1) for 72 h. Scale bar: 100 μm.

(G) Representative images showing HPV16 PsV-derived GFP signal in hCerEC-CTRL cells (hCerECs transfected with non-targeting scrambled short interfering RNA [siRNA] as control) and hCerEC-siYAP cells (hCerECs transfected with YAP1-specific siRNA). Cells were incubated in growth media with HPV16 PsV (MOI = 1) for 72 h. Scale bar: 100 μm.

(H) Representative images showing HPV16 PsV-derived GFP signal in Ect1-MX, Ect1-YAP, and Ect1-YAP^{S127A} cells. Ect1 cells is an immortalized cervical epithelial cell line. Scale bar: 100 μm.

(I) Representative images showing HPV16 PsV-derived GFP signal in SiHa-CTRL and SiHa-siYAP cells. SiHa is a cervical cancer cell line. Scale bar: 100 μm. Quantitative data are presented in Figure S5.

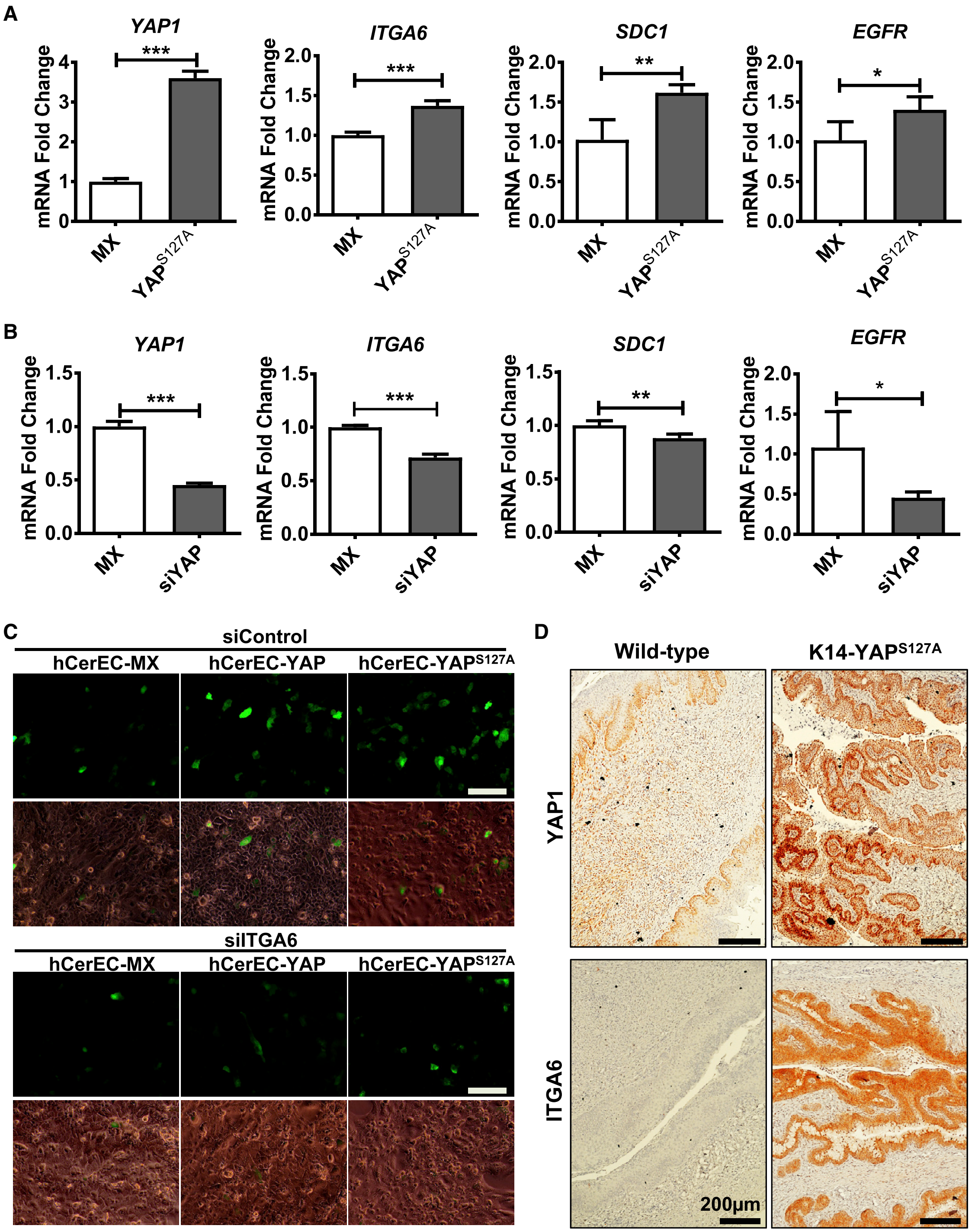

Figure 3. Hyperactivation of YAP1 Upregulates Expression of the Putative HPV Receptor Molecules *In Vitro* and *In Vivo*

(A) RT-PCR analyses showing relative expression levels of the putative HPV receptors, including *ITGA6*, *SDC1*, and *EGFR*, in hCerEC-MX and hCerEC-YAP^{S127A} cells. Each bar represents mean ± SEM (n = 4). *p < 0.05, **p < 0.01, and ***p < 0.001.

(B) RT-PCR analyses showing expression of *ITGA6*, *SDC1*, and *EGFR* in SiHa-CTRL and SiHa-siYAP cells. Each bar represents mean ± SEM (n = 4). *p < 0.05, **p < 0.01, and ***p < 0.001.

(C) Representative images showing HPV16 PsV-derived GFP signal in hCerEC-MX, hCerEC-YAP, and hCerEC-YAP^{S127A} cells with (siITGA6) or without (siControl) knockdown of ITGA6 protein. Scale bar: 100 μm.

(D) Representative images showing expressions of YAP1 and the well-studied putative HPV receptor molecule ITGA6, in cervical tissues derived from 10-month-old control (KRT-rtTA mice) and KRT14-YAP^{S127A} transgenic mice. Protein expression was determined using peroxidase-based immunohistochemistry. The nuclei were counterstained with hematoxylin. Note upregulation of ITGA6 protein in the epithelium of KRT14-YAP^{S127A} transgenic mice. Scale bar: 200 μm.

OPEN
ACCESS
CellPress

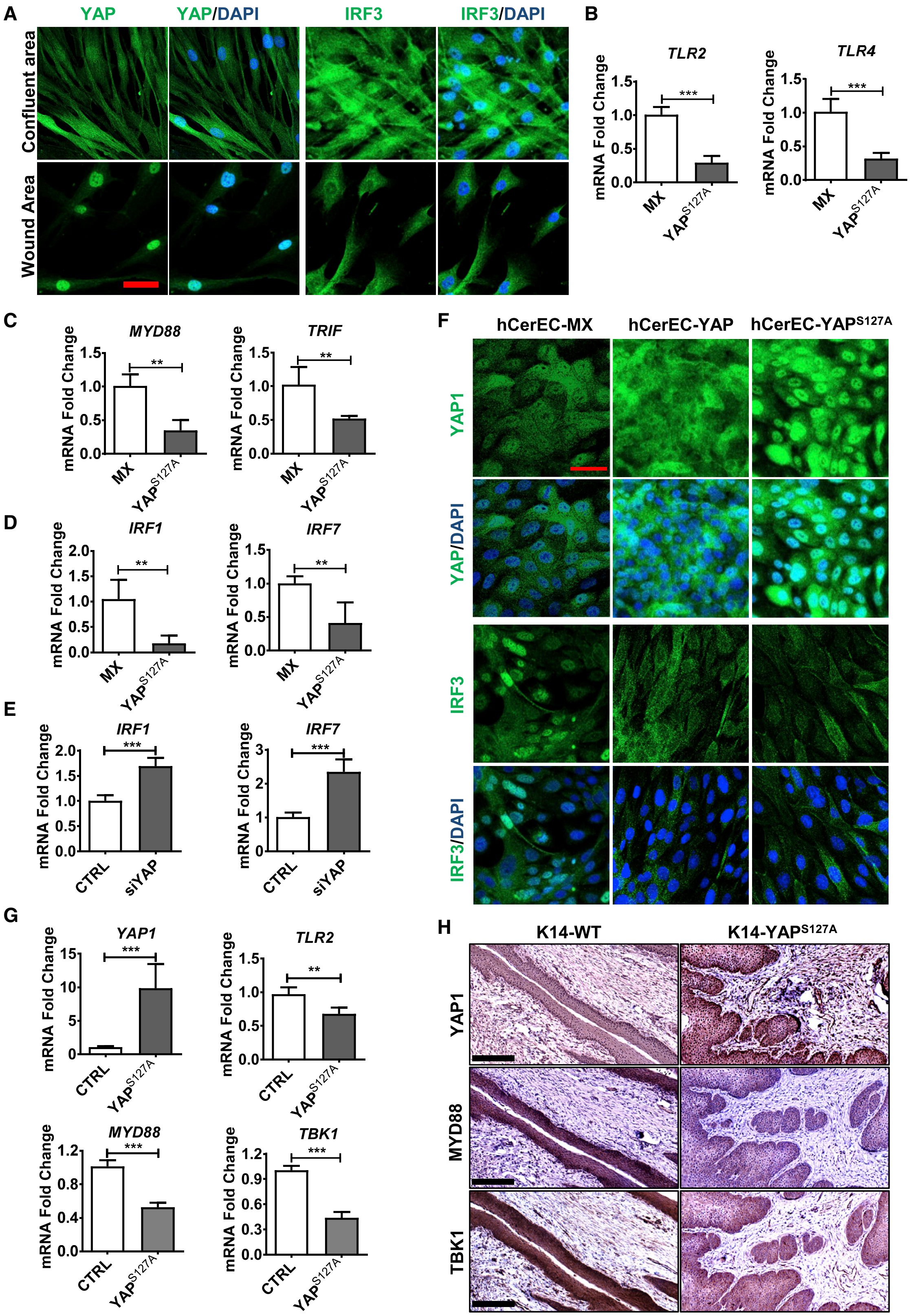

(legend on next page)

Following viral entry, the first step in activating an innate immune response against viral infection is the detection of viral pathogens, which is mediated by the interaction between pattern recognition receptors (PPRs) of host cells and the pathogen-associated molecular patterns (PAMPs) of HPV virions (Stanley, 2012). Our RT-PCR results suggested that mRNA expression of the well-studied PRRs for HPV, Toll-like receptors (TLRs) *TLR2* (Alphs et al., 2008) and *TLR4* (Pannone et al., 2016), was significantly downregulated in YAP^{S127A}-expressing cells (Figure 4B). The signal transduction from TLRs to downstream kinase cascades requires the mediation of several adaptor proteins, including myeloid differentiation primary response gene 88 (MYD88) and TIR-domain-containing adaptor-inducing interferon-β (TRIF) (Tartey and Takeuchi, 2017). The expression of *MYD88* and *TRIF* mRNA was also inhibited by YAP^{S127A} in hCerECs (Figure 4C). Western blot analysis further confirmed the downregulation of TLR2 and TRIF in hCerECs overexpressing YAP1 (Figure S8A). These results indicated that hyperactivation of YAP1 can negatively regulate the viral recognition pathway.

The recognition of HPV PAMPs by PRRs may trigger a series of kinase cascades that subsequently leads to activation and nuclear translocation of IRF1, IRF3, IRF7, and NFκB, which are transcription factors regulating expression of type I interferons (IFNα and IFNβ) and cytokines, the key molecules for antiviral immune response (McNab et al., 2015; Takeuchi and Akira, 2009). Similar to TLRs in hCerEC-YAP^{S127A} cells, expression of *IRF1* and *IRF7* genes was also significantly decreased in YAP1- hyperactivated cells (Figure 4D). Consistently, knockdown of YAP1 in these cells significantly increased expression of IRF1 and IRF7 mRNA levels (Figure 4E). Although the level of IRF3 protein was not significantly affected by YAP1 expression, the level of phospho-IRF3 (active form) was decreased in hCerECs expressing YAP^{S127A} (Figure S8A). Immunofluorescent analysis also showed that ectopic expression of YAP1 or YAP^{S127A} resulted in translocation of IRF3 and NFkB1 from nucleus to cytoplasm in hCerECs, suggesting the transcriptional activity of IRF3 and NFkB1 was inhibited in YAP- and YAP^{S127A}-overexpressed cells (Figures 4F, S8A, and S8B).

Consistent with *in vitro* results, critical genes involved in viral recognition signaling, such as *TLR2*, *MYD88*, and *TBK1*, were significantly downregulated in KRT14-YAP^{S127A} mice (Figure 4G). IHC staining results further demonstrated that in comparison with the control cervical tissues, the cervical epithelial layer of KRT14-YAP^{S127A} mice expressed significantly lower levels of MYD88 and TBK1 (Figures 4H and S8C), suggesting that hyperactivation of YAP1 attenuates the innate immune system *in vivo*.

Hyperactivation of YAP1 Interrupts the IFNRs-JAKs-STATs Signaling Pathway to Inhibit Production of Antiviral ISGs in Cervical Epithelial Cells

Normally, the secreted IFNs bind to IFN receptors on adjacent cells, resulting in the activation of the JAK-STAT pathway in these cells (Figure S7B). Phosphorylated STAT1 and STAT2 then bind with IRF9 to form a complex called IFN-stimulated gene factor 3 (ISGF3) to initiate the transcription of hundreds of genes that act to block viral infection (Schneider et al., 2014). The RT-PCR results showed that mRNA expressions of IFNα receptor 2 (*IFNAR2*), *JAK1*, JAK2, *STAT1*, and *IRF9* were downregulated in hCerEC-YAP^{S127A} cells (Figures 5A and Figure S9A), while mRNA expression of *IFNAR1* and *IRF9* was upregulated in YAP1-knockdown hCerECs (Figure 5B). Immunoblotting analysis also detected reduced levels of JAK1, STAT1, phospho-STAT1, and IRF9 proteins in YAP-overexpressed cells (Figure 5C). These results indicated that hyper-active YAP1 could inhibit the signal transduction from type I IFNs to ISGF3. Consistent with this, immunofluorescence confocal microscopy showed that compared with control (MX) cells, ectopic expression of YAP1 or YAP^{S127A} not only reduced the signal intensity of total STAT1 and IRF9 but also decreased the immunosignal of nuclear STAT1 and IRF9. Treatment of hCerECs with recombinant IFNα2β (50 IU, 45 min) rapidly induced nuclear accumulation of STAT1 and IRF9 in hCerEC-MX cells, but it failed to do so in hCerEC-YAP and hCerEC-YAP^{S127A} cells (Figure S9B). We also noticed that in control cells, IRF9 and STAT1 formed multiple foci in the nucleus. However, the focal pattern of IRF9 and STAT1 was diminished in YAP^{S127A}-expressed cells (Figure S9B), indicating that the transcriptional activity of ISGF3 complex was inhibited by YAP1 hyperactivation. Similar results were observed in wound-healing assay (Figure S10).

Type I interferons (mainly IFNα and IFNβ) can induce expressions of a large spectrum of interferon-stimulated genes (ISGs) in virus-infected cells as well as neighbor cells. IFN-induced ISGs are involved in almost all key steps of antiviral effects in host cells, including inhibition of virus entry, blockade of virus replication, and obstruction of viral egress (Schneider et al.,

Figure 4. Hyperactivation of YAP1 in Cervical Epithelial Cells Inactivates Signaling Pathways Involved in Type I Interferon Production *In Vitro* and *In Vivo*

(A) Representative images from *in vitro* experiments showing expression and location of YAP1 and IRF3 in normal and "wound" area of cultured hCerECs. YAP1 and IRF3 proteins were visualized using an Alexa 488 (green)-conjugated secondary antibody. Nuclei were stained with DAPI (blue). Scale bar, 20 μm.

(B and C) Quantitative data showing mRNA levels of *TLR2* and *TLR4* (B), and their adapter proteins MYD88 and TRIF (C) in hCerEC-MX (control) and hCerEC-YAP^{S127A} cells. Each bar represents mean ± SEM (n = 4). **p < 0.01 and ***p < 0.001.

(D and E) Quantitative data showing mRNA levels of *IRF1* and *IRF7* in hCerEC-MX (control) and hCerEC-YAP^{S127A} cells (D) and in control (CTRL) and YAP1-knockdown hCerECs (siYAP) (E). Each bar represents the mean ± SEM (n = 4). **p < 0.01 and ***p < 0.001.

(F) Representative images showing the expression and location of YAP1 and IRF3 in hCerEC-MX (control), hCerEC-YAP, and hCerEC-YAP^{S127A} cells. YAP1 and IRF3 proteins were visualized using an Alexa 488 (green)-conjugated secondary antibody. Nuclei were stained with DAPI (blue). Scale bar, 20 μm.

(G) RT-PCR analyses showing expressions of *YAP1*, *TLR2*, *MYD88*, and *TBK1* mRNA in cervical tissues from KRT14-rtTA control (CTRL) and KRT14-YAP^{S127A} mice. Each bar represents the mean ± SEM (n = 4). **p < 0.01 and ***p < 0.001.

(H) Representative images showing expression of YAP1, MYD88, and TBK1 proteins in cervical tissues from KRT14-rtTA control (CTRL) and KRT14-YAP^{S127A} mice. Protein expression was determined using peroxidase-based immunohistochemistry. The nuclei were counterstained with hematoxylin. Note upregulation of YAP1 and downregulation of MYD88 and TBK1 proteins in the epithelium of KRT14-YAP^{S127A} transgenic mice. Scale bar: 250 μm.

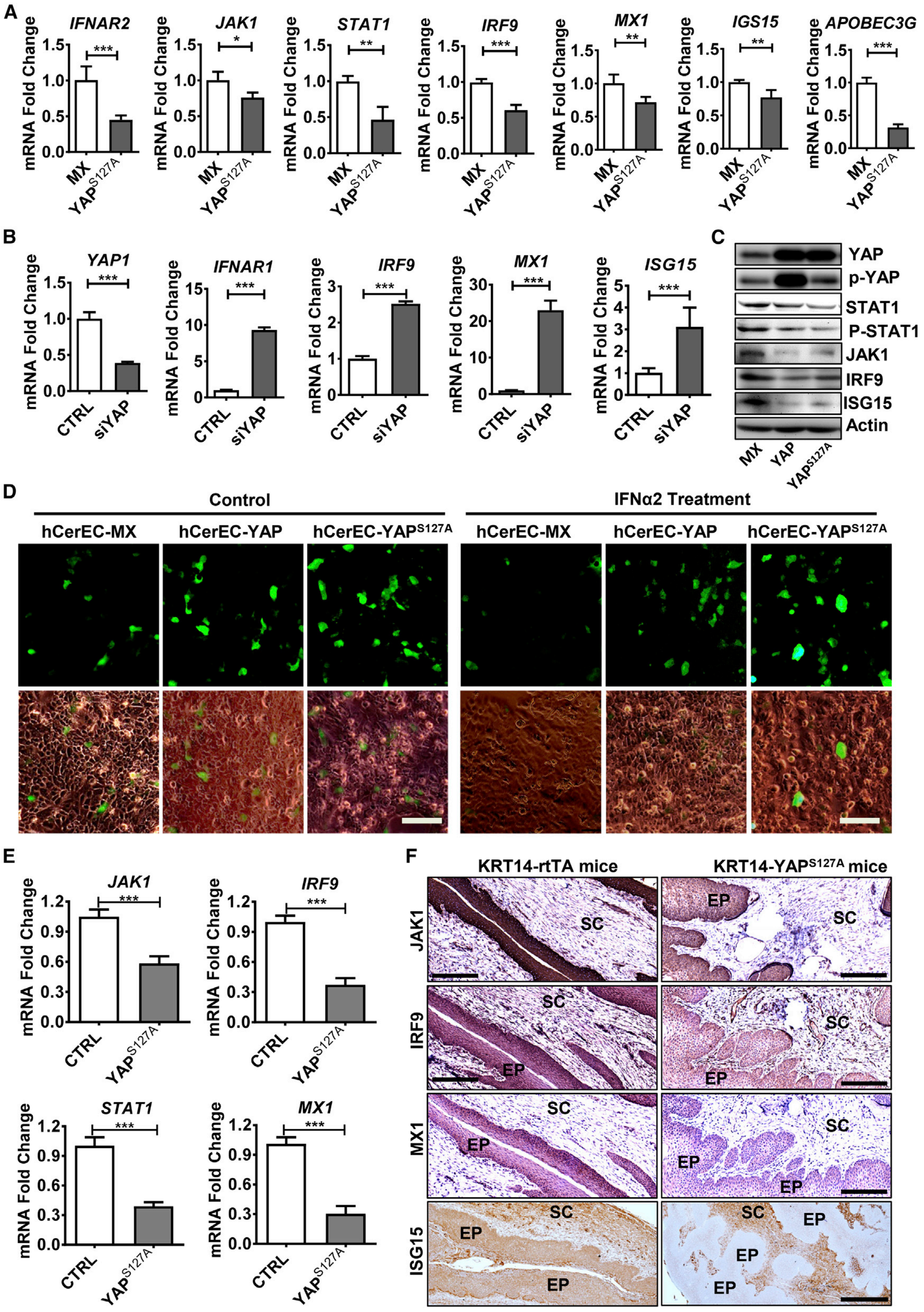

Figure 5. Hyperactivation of YAP1 Suppresses the IFNs/JAKs/STATs Pathway in Cervical Epithelial Cells

(A) Quantitative experiments showing mRNA levels of major components of the IFNRs/JAK/STAT/IRF9 pathway and downstream target genes in control (hCerEC-Mx) and hCerEC-YAP^{S127A} cells. Each bar represents the mean ± SEM (n = 4). *p < 0.05, **p < 0.01, and ***p < 0.001.

(legend continued on next page)

2014). As expected, expression of genes encoding ISGs that directly inhibit virus infection, such as *MX1* (myxovirus resistance 1), *ISG15*, *APOBEC3G* (apolipoprotein B mRNA editing enzyme, catalytic polypeptide-like 3G), *OAS1* (2′-5′-oligoadenylate synthetase 1), *TRIM5* (tripartite motif family protein 5), and *IFI44L*, was significantly downregulated in YAP^{S127A}-expressed hCerECs (Figures 5A and S9A). Consistently, knockdown of YAP1 in hCerECs upregulated many antiviral ISGs, such as *MX1*, *CH25H*, *IFITM*s (inhibitors of virus entry), and *APOBEC3G*, *OAS1/2*, *ISG15*, and *IFI44L* (suppressors of virus translation and replication) (Figures 5B and S11A). Taken together, these results provide strong evidence that constitutive activation of YAP1 can suppress the IFNαRs-JAKs-STATs pathway and reduce the production of antiviral ISGs.

To further examine the role of hyperactivated YAP1 on the IFN-induced antiviral effect, we pretreated control and YAP-overexpressing hCerECs with recombinant IFNα2b (50 IU) for 24 h and then incubated these cells with HPV16 PsVs (MOI = 1.0) for 72 h. IFNα2β treatment resulted in a significant decrease in the number of GFP-positive cells in control hCerECs (Figures 5D and S11B). However, IFNα2β had no effect on the positivity and intensity of GFP signal in hCerEC-YAP and hCerEC-YAP^{S127A} cells (Figures 5D and S11B). These results, combined with the above data, suggested that hyperactivation of YAP1 in cervical epithelial cells not only suppresses the production of type I IFNs but may also diminish the antiviral effects of IFNs.

Consistent with *in vitro* results, expression of genes encoding the core components of the JAK-STAT/IRF9 pathway, such as *JAK1*, *STAT1*, and *IRF9*, were also significantly reduced in KRT14-YAP^{S127A} mice (Figure 5E). IHC staining results further demonstrated that in comparison with the control cervical tissues, cervical epithelial layer of KRT14-YAP^{S127A} mice expressed significantly lower levels of JAK1 and IRF9 proteins (Figures 5F and S11C). MX1 and ISG15, the two best characterized antiviral ISGs, were diminished at both transcriptional (mRNA) and protein levels in KRT14-YAP^{S127A} mice (Figures 5E, 5F, and S11C). These results provide evidence that hyperactivation of YAP1 attenuates the innate immune system *in vivo*.

HPV Synergizes with YAP1 to Promote Carcinogenesis of Cervical Epithelium *In Vivo*

Although hyperactivation of YAP1 is sufficient to induce development of cervical cancer, it generally took 6–8 months to observe invasive cancer in the cervical epithelium under constitutive induction of YAP1 expression with low concentration of Dox. The observation that hyperactivation of YAP1 induced expression of the putative HPV receptors and suppression of innate immune system in cervical epithelial cells indicates that HPV and YAP1 might function in a synergic manner to drive the development of cervical cancer. To examine the potential synergism of hyperactivated YAP1 and HPV oncoproteins, we used KRT14-E6 (HPV16) mice, KRT14-E7 (HPV16) mice (Riley et al., 2003), and KRT14-YAP^{S127A} mice to create two new transgene mouse models, KRT14-E6-YAP^{S127A} and KRT14-E7-YAP^{S127A} mice (Figures 6A and 6B). These mice express both HPV oncoprotein E6 (or E7) (under control of KRT14 promoter) and YAP^{S127A} (under control of KRT14 and Dox induction) in cervical epithelial cells. Consistent with previous reports (Riley et al., 2003), KRT14-E6 and KRT-E7 mice rarely developed cervical cancer without exposure to a relatively high concentration of estrogen, even after treatment with a low concentration of Dox for 1.5 years. Most KRT14-YAP^{S127A} mice, as shown before, developed cervical cancer after 6–8 months of Dox induction. However, we found that most of KRT14-E6-YAP^{S127A} and KRT14-E7-YAP^{S127A} mice developed invasive cervical cancer within 4 months of induction (Figure 6C). Compared with KRT14-YAP^{S127A} mice, cancer progression in KRT14-E6-YAP^{S127A} and KRT14-E7-YAP^{S127A} mice was much faster than that in KRT14-YAP^{S127A} mice. After induction with a low concentration of Dox for 9 months, about 30% of KRT14-YAP^{S127A} mice were dead (or euthanized because of urethral obstruction). However, the death rate in KRT14-E6-YAP^{S127A} and KRT14-E7-YAP^{S127A} mice reached 60% and 75%, respectively (Figures 6D and 6E). Cancer cells in the cervical tissue of KRT14-E6/E7-YAP^{S127A} mice were more invasive and aggressive than in KRT14-YAP^{S127A} mice (Figure 6C). These *in vivo* data strongly support our hypothesis that HPV synergizes with hyperactivated YAP1 to drive initiation and progression of cervical cancer.

DISCUSSION

Novel Mouse Models Suggest that HPV Is Not a Necessary Cause of Cervical Cancer

The recognition of HPV infection as a major risk factor for cervical cancer has been considered a breakthrough in cancer research in the past three decades. During the 1990s, epidemiological studies, supported by molecular technologies, concluded that persistent high-risk HPV (hrHPV) infection was a necessary cause of cervical cancer, implying that cervical cancer does not and will not form in the absence of persistent HPV infection

(B) Quantitative data showing mRNA levels of *YAP1* and genes of the core components of the IFNR/JAK/STAT antiviral pathway in hCerEC-CTRL cells (cells transfected with scrambled non-target siRNA) and hCerEC-siYAP cells (cells transfected with YAP1-specific siRNA). Each bar represents the mean ± SEM (n = 4). *p < 0.05, **p < 0.01, and ***p < 0.001.

(C) Representative blots from three independent experiments showing expression and activation of key proteins and kinases in the JAK/STAT/IRF9 pathway in hCerEC-MX (control), hCerEC-YAP, and hCerEC-YAP^{S127A} cells.

(D) Representative images showing HPV16 PsV-derived GFP signal in hCerEC-MX, hCerEC-YAP, and hCerEC-YAP^{S127A} cells in the presence or absence of recombined human interferon alpha 2b (IFNα2b). GFP signal indicates the infection efficiency of HPV16 PsV in these cells. Scale bar: 100 μm. Quantitative results of GFP signal is presented in Figure S11B.

(E) RT-PCR analyses showing expression of *JAK1*, *IRF9*, *STAT1*, and *MX1* mRNA in cervical tissues from control KRT14-rtTA (CTRL) and KRT14-YAP^{S127A} mice. Each bar represents the mean + SEM (n = 4). ***p < 0.001.

(F) Representative images showing expression of JAK1, IRF9, MX1, and ISG15 proteins in cervical tissues from KRT14-rtTA control (CTRL) and KRT14-YAP^{S127A} mice. Protein expression was determined using the peroxidase-based immunohistochemistry. The nuclei were counterstained with hematoxylin. Scale bar: 250 μm.

OPEN
ACCESS
CellPress

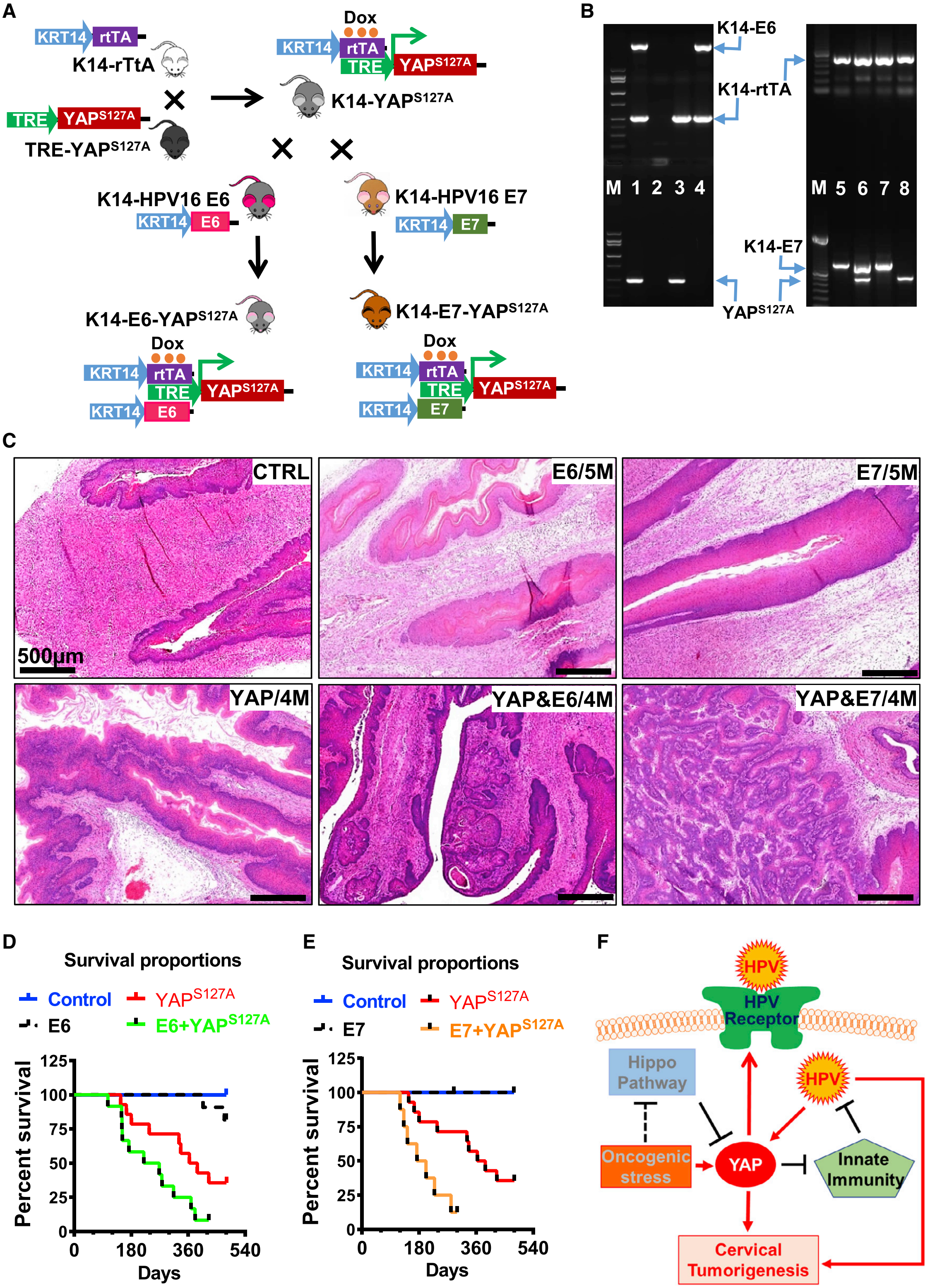

Figure 6. HPV Oncoproteins Accelerate Cervical Cancer Initiation and Progression in KRT14-YAP^{S127A} Transgenic Mice

(A) A schematic diagram showing the mating strategy for generating of KRT14-E6-YAP^{S127A} and KRT14-E7-YAP^{S127A} transgenic mice.

(B) Representative images showing PCR products of transgenes (genotyping results) in tissues extracted from wild-type (lane 2), KRT14-YAPS127A (lanes 3 and 8), KRT14-E6 (lane 4), KRT14-E6-YAP^{S127A} (lane 1), KRT14-E7 (lanes 5 and 7), and KRT14-E7-YAP^{S127A} (lane 6) transgenic mice.

(legend continued on next page)

(Schiffman and Castle, 2003; Walboomers et al., 1999). However, although the estimated lifetime risk for HPV infection can be 75% (Koutsky, 1997), the lifetime risk for developing cervical cancer is only ∼0.68% (Howlader et al., 2012). Moreover, cervical cancer occurs many years after HPV infection (Howlader et al., 2012; Weaver, 2006). In addition, cervical cancer shows very high intratumor heterogeneity (Cancer Genome Atlas Research Network et al., 2017; Lyng et al., 2004). These observations indicate that exposure to HPV alone is insufficient for cervical cancer development (Perez-Plasencia et al., 2008; Uren et al., 2005). Animal studies showed that cervical epithelial specific expression of HPV16 E6, E7, or their combination did not induce cervical cancer without support of exogenous estrogen (Brake and Lambert, 2005; Riley et al., 2003). Accumulating evidence suggests that the presence of a persistent hrHPV infection risk is not sufficient to immortalize and transform the epithelial cells of the host (Gatza et al., 2005; Roden and Wu, 2006). Currently, it is widely accepted that that persistent HPV infection is a necessary but insufficient cause for the carcinogenesis of cervical epithelium (National Cancer Institute, 2018). Existing evidence indicates that the presence of genetic and epigenetic alterations is necessary for the carcinogenesis of cervix, although the specific alteration or alterations are still not clear. In the present study, we provide *in vitro* and *in vivo* evidence to show that pathological alterations of the Hippo/YAP signaling pathway are sufficient to induce cervical cancer, regardless HPV infection. However, the synergism between HPV oncoprotein and YAP1 oncogene significantly accelerates tumorigenesis of the cervix.

Central Role and Functional Mechanism of YAP1 in Cervical Cancer Development

The role of the Hippo/YAP1 signaling pathway in cancer development has been intensively studied over the past 10 years (Mo et al., 2014; Pan, 2010; Yu et al., 2015; Zanconato et al., 2016). Both *in vitro* and *in vivo* evidence indicates that dysregulation of the Hippo signaling cascade and activation of proto-oncogene *YAP1* are linked to many human cancers, including cancers of the skin, lung, colon, breast, kidney, liver, and ovary (Gomez et al., 2014; He et al., 2015a; Lee et al., 2010; Steinhardt et al., 2008). Cross-cancer genomic analyses based on TCGA datasets showed that the most frequent genomic alteration of the Hippo/YAP1 pathway occurs in the cervical carcinoma. This observation is supported by recent genome-wide screening by TCGA, which also showed that the copy number of genes in chromosome 11q22 was frequently amplified in CVSCC. Interestingly, YAP1 and its downstream target genes BACR2 and BACR3 are located in this area (Cancer Genome Atlas Research Network et al., 2017). On the basis of DNA methylation status, the authors identified a sub-group of cervical cancer that had epithelial–mesenchymal transition (EMT) features and was associated with the worst survival outcomes. Importantly, they found that *YAP1* gene was amplified in cancer cells derived from the EMT cluster, and YAP1 protein was the most significantly differentially expressed protein that distinguished the EMT cluster from other sub-groups (Cancer Genome Atlas Research Network et al., 2017). We mined TCGA cervical cancer datasets and found that besides *YAP1* amplification, the upstream genes of the Hippo pathway, including *MST1*, *LATS1/2*, and *FAT1/2/3/4*, which negatively regulate YAP1 activity, were frequently deleted and/or mutated in cervical cancer patient samples. Our previous data also showed that hyperactivation of YAP1 in hCerECs induced malignant transformation of these cells (He et al., 2015b). The development of CVSCC in KRT14-YAP^{S127A} mice, which are HPV negative, strongly supports the notion that the disrupted Hippo pathway and the subsequent hyperactivation of YAP1 represent a novel mechanism of cervical carcinogenesis.

The precise mechanism for the Hippo/YAP pathway regulating cervical cancer cell growth remains largely undefined. Our recent study demonstrated that overexpression of YAP1 in cervical cancer cells can overcome the contact inhibition-induced cell growth inhibition, promote cell cycle progression, and significantly stimulate cervical cancer cell growth *in vitro* and *in vivo* (He et al., 2015b). Knockdown of YAP1-negative regulators LATS1/2 resulted in a significant increase in the cervical epithelial cell proliferation. Moreover, we found that the EGFR pathway was involved in YAP1-induced cervical cancer cell proliferation and migration. Activation of YAP1 in cervical cancer cells significantly stimulated the expression of EGFR and its ligands TGFα and AREG. In turn, TGFα and AREG, via EGFR signaling, suppressed the Hippo pathway and activated YAP protein (dephosphorylated LATS1, MOB1, and YAP1) to promote cervical cancer cell growth (He et al., 2015b). The existence of this Hippo/YAP and EGFR feedback loop in human cervical cancer suggested that the combined targeting of the Hippo/YAP and EGFR pathways may be an efficient way to treat cervical cancer.

Previous studies showed that HPV infection is a necessary cause of cervical cancer. In reality, following infection, the majority of HPV becomes undetectable within 1–2 years, and the virus is cleared spontaneously by the immune system. Nevertheless, persistent HPV infection happens in some high-risk individuals and increases the risk for cervical cancer in these women. The mechanism by which HPV evades immune surveillance in these patient is not clear. Some reports suggest that HPV modifies the innate immune system in the host cells, leading to the failure of virus clearance and persistent HPV infection (Scott et al., 2001; Westrich et al., 2017). However, this modification requires HPV

(C) Representative images showing histology of cervical tissues (H&E staining) collected from control, KRT14-E6/E7, KRT14-YAP^{S127A}, KRT14-E6-YAP^{S127A}, and KRT14-E7-YAP^{S127A} mice after induction of YAP^{S127A} gene expression with doxycycline in drinking water for 4–5 months.

(D) Kaplan-Meier survival curves showing the overall survival rates of control mice (control, n = 12), KRT-14-YAP^{S127A} mice (YAP^{S127A}, n = 14), KRT14-E6 mice (E6, n = 13), and KRT14-E6-YAP^{S127A} mice (E6+YAP^{S127A}, n = 12) after inducing expression of transgenes with 0.05 mg/mL doxycycline (in drinking water).

(E) Kaplan–Meier survival curves showing the overall survival rates of control mice (control, n = 9), KRT-14-YAP^{S127A} mice (YAP^{S127A}, n = 14), KRT14-E7 mice (E7, n = 7), and KRT14-E7-YAP^{S127A} mice (E7+ YAP^{S127A}, n = 8) after inducing expression of transgenes with 0.05 mg/mL doxycycline (in drinking water).

(F) A schematic diagram showing the proposed mechanism by which hyperactivated YAP1 and high-risk HPVs synergize to drive the initiation and progression of cervical cancer.

E6 and E7 oncogene expression, which happens after HPV initial infection. Therefore, unknown alterations in the defense system of the vulnerable individuals may contribute to the establishment of the persistent HPV infection. In the present study, we found that hyperactivation of YAP1 may be a major contributor to the HPV persistent infection. First of all, results in the present study showed that hyperactivated YAP1 upregulated putative HPV receptor molecules such as ITGRA6, SDC1, and EGFR, which may facilitate the HPV entry process during early stage of HPV infection. Second, we found that hyperactivation of YAP1 in cervical epithelial cells downregulated key components of viral recognition by the innate immune system. We observed that TLR2 and TLR4, well-studied PRRs, as well as their adaptor proteins MYD88 and TRIF, were inhibited, which could exacerbate HPV infection. Third, we found that ectopic expression of YAP^{S127A} inhibited the expression and activation of transcriptional factors that are critical for the production of type I IFNs, including IRF1, IRF3, and IRF7, resulting in decreases of IFNA1, IFNB1, and IFNE in hCerECs. These IFNs can activate intracellular antiviral programs and play a key role in the development of innate and adaptive immune responses. Finally, we observed that the expression and activation of key components of the type I IFN pathway, including IFNAR2, JAK1, STAT1, and IRF9, were broadly inhibited by expression of YAP^{S127A} in cervical epithelial cells. Inhibition the IFNAR2/JAK1/STAT1 signaling pathway resulted in massive downregulation of antiviral ISGs, including *MX1*, *CH25H*, and *IFITMs* (inhibitors of virus entry) and *APOBEC3G*, *OAS1*, *ISG15*, and *IFI44L* (suppressors of virus translation and replication). More important, the inhibition of innate antiviral pathways by YAP was confirmed in studies with the K14-YAP^{S127A} transgenic mouse. Therefore, the present studies indicate that disruption of the Hippo pathway and subsequent activation of YAP1 in the cervical epithelial may result in defective innate antiviral immunity, which may allow HPV to escape immune surveillance, leading to persistent HPV infection. Consistent with this observation, two research groups in China recently reported that YAP1 negatively regulated the production of type I interferon by suppressing TBK1 activity (Zhang et al., 2017; Wang et al., 2017). In the *Drosophila* model, hyperactivation of Yorkie (YAP in mammals) also led to the decrease of antimicrobial peptides in adipose tissues, suggesting that YAP1 regulation of innate immunity is a conserved mechanism of pathological immune suppression (Liu et al., 2016). These data suggest that enhancing HPV infection may represent a new mechanism for YAP1 to control cervical cancer development.

Identification of the Hippo/YAP Pathway as a Major Player in Cervical Cancer Development Does Not Reduce the Importance of hrHPV in Cervical Tumorigenesis

Although our transgenic mouse model demonstrates that HPV infection is not a necessary event for the development of CVSCC, our present data, plus previous numerous reports, still support the notion that hrHPV infection, especially in vulnerable populations, greatly increases the risk for cervical cancer. Therefore, identification of the Hippo/YAP pathway as a major player in cervical carcinogenesis does not reduce the importance of hrHPV in the development of cervical cancer. Our data clearly indicate that hyperactivation of YAP1 increased viral infectivity and suppressed innate immunity, two events that greatly increase the susceptibility of cervical epithelial cells to HPV infection and facilitate the establishment of persistent HPV infection in these cells. Although HPV alone is not sufficient to induce malignant transformation of cervical epithelial cells, expression of HPV oncoproteins E6 and E7 leads to inactivation of critical tumor suppressors such as TP53 and RB1 in many types of cells (Dyson et al., 1989; Scheffner et al., 1990; White et al., 2016). Inactivation of TP53 and RB1 can lead to extension of cell life (via suppression of senescence) and even induce immortal phenotype in cervical epithelial cells (Liu et al., 2018), paving the way for malignant transformation of HPV-infected cells by an oncogenic signal (such as hyperactivation of YAP1). Moreover, we have shown that HPV can prevent proteinase-dependent degradation of YAP1 protein, which maintains high levels of YAP1 protein in HPV-infected cervical epithelial cells. Most important, we found that HPV E6 protein suppressed Hippo pathway in the cervical epithelial cells (He et al., 2015b). Clearly, HPV synergizes with hyperactivated YAP1 to drive carcinogenesis of cervix. Introduction of HPV16 E6 and E7 oncoproteins significantly promoted cervical carcinogenesis of KRT14-YAP^{S127A} cells, indicating that synergetic function between YAP1 and HPV does exist during cervical tumorigenesis. Therefore, the current HPV vaccination program, which has been shown to be very effective in preventing selected types of HPV (Baldur-Felskov et al., 2014; Sankaranarayanan, 2015), may greatly reduce infection by HPV and carcinogenesis of the cervix in women in the near future.

In conclusion, the *in vitro* and *in vivo* results from the present study show that YAP1, the major effector of the Hippo pathway, plays a central role in the development of cervical cancer. Hyperactivation of YAP1 is sufficient to induce malignant transformation of cervical epithelial cells and development of invasive cervical cancer in mouse models. Disruption of the Hippo pathway and subsequent activation of YAP1 in cervical epithelial cells may facilitate the establishment of persistent HPV infection by upregulating putative HPV receptor molecules and suppressing host cell innate immunity. Together with our previous observations that HPV16 E6 protein is able to suppress the Hippo pathway (thereby activating YAP1) and prevent YAP1 from proteasome-mediated degradation, we conclude that the interaction between the Hippo/YAP pathway hrHPV is a key player in cervical carcinogenesis. The present findings are expected to influence current cervical cancer preventive screening, early detection, and treatment. The current HPV vaccination program may greatly reduce infection by HPV and carcinogenesis of the cervix in women in the near future. The novel mouse models developed in this study provide new tools for further investigation of cervical cancer initiation and progression.

STAR★METHODS

Detailed methods are provided in the online version of this paper and include the following:

- KEY RESOURCES TABLE
- CONTACT FOR REAGENT AND RESOURCE SHARING

- EXPERIMENTAL MODEL AND SUBJECT DETAILS
  - Mouse Model Studies
  - Cell Culture
  - Human Cervical Tissue Microarray
- METHOD DETAILS
  - Reagents and Materials
  - IHC
  - Fluorescent Immunocytochemistry
  - Western Blot Analysis
  - Quantitative Real Time-PCR
  - YAP1 Overexpression
  - Gene Silencing by siRNA
  - Preparation of HPV PsVs
  - HPV16 PsV Infection of Cells
- QUANTIFICATION AND STATISTICAL ANALYSIS

SUPPLEMENTAL INFORMATION

Supplemental Information can be found with this article online at https://doi.org/10.1016/j.celrep.2019.02.004.

ACKNOWLEDGMENTS

This work was supported by the National Cancer Institute (1R01CA197976 and 1R01CA201500); the Colleen's Dream Foundation; the Olson Center for Women's Health; the Fred & Pamela Buffett Cancer Center (LB595); the Marsha Rivkin Center for Cancer Research (the Barbara Learned Bridge Funding Award); the Vincent Memorial Hospital Foundation; and the Vincent Center for Reproductive Biology.

AUTHOR CONTRIBUTIONS

C. He contributed to experimental design, performance, data analysis, and manuscript preparation. X.L. contributed to studies related to animal model establishment, data analysis, and manuscript preparation. C. Huang conducted IHC and immunofluorescence (IF) analyses and manuscript preparation. G.H. packaged the retrovirus and performed some gene knockdown experiments. J.D. constructed YAP retroviral vectors. P.C.A. contributed to the construction of HPV16 E6/E7 expression vectors and establishment of the HPV16 PsVs. J.Z. contributed to histopathological analysis. B.M. and X.C. performed real-time PCR analysis. Z.W. contributed to western blotting analysis. P.F.L. contributed to the establishment of HPV-associated mouse models. B.R.R. contributed to manuscript preparation. J.S.D. contributed to data analysis and manuscript preparation. C.W. supervised these studies and contributed to experimental design, data analysis, and manuscript preparation.

DECLARATION OF INTERESTS

The authors declare no competing interests.

Received: September 5, 2018
Revised: December 30, 2018
Accepted: February 1, 2019
Published: March 5, 2019

REFERENCES

Alphs, H.H., Gambhira, R., Karanam, B., Roberts, J.N., Jagu, S., Schiller, J.T., Zeng, W., Jackson, D.C., and Roden, R.B. (2008). Protection against heterologous human papillomavirus challenge by a synthetic lipopeptide vaccine containing a broadly cross-neutralizing epitope of L2. Proc. Natl. Acad. Sci. U S A *105*, 5850–5855.

Amador-Molina, A., Hernández-Valencia, J.F., Lamoyi, E., Contreras-Paredes, A., and Lizano, M. (2013). Role of innate immunity against human papillomavirus (HPV) infections and effect of adjuvants in promoting specific immune response. Viruses *5*, 2624–2642.

Baldur-Felskov, B., Dehlendorff, C., Munk, C., and Kjaer, S.K. (2014). Early impact of human papillomavirus vaccination on cervical neoplasia–nationwide follow-up of young Danish women. J. Natl. Cancer Inst. *106*, djt460.

Brake, T., and Lambert, P.F. (2005). Estrogen contributes to the onset, persistence, and malignant progression of cervical cancer in a human papillomavirus-transgenic mouse model. Proc. Natl. Acad. Sci. U S A *102*, 2490–2495.

Camargo, F.D., Gokhale, S., Johnnidis, J.B., Fu, D., Bell, G.W., Jaenisch, R., and Brummelkamp, T.R. (2007). YAP1 increases organ size and expands undifferentiated progenitor cells. Curr. Biol. *17*, 2054–2060.

Cancer Genome Atlas Research Network; Albert Einstein College of Medicine; Analytical Biological Services; Barretos Cancer Hospital; Baylor College of Medicine; Beckman Research Institute of City of Hope; Buck Institute for Research on Aging; Canada's Michael Smith Genome Sciences Centre; Harvard Medical School; and Helen F. Graham Cancer Center &Research Institute at Christiana Care Health Services, et al. (2017). Integrated genomic and molecular characterization of cervical cancer. Nature *543*, 378–384.

Cerami, E., Gao, J., Dogrusoz, U., Gross, B.E., Sumer, S.O., Aksoy, B.A., Jacobsen, A., Byrne, C.J., Heuer, M.L., Larsson, E., et al. (2012). The cBio cancer genomics portal: an open platform for exploring multidimensional cancer genomics data. Cancer Discov. *2*, 401–404.

Conway, M.J., and Meyers, C. (2009). Replication and assembly of human papillomaviruses. J. Dent. Res. *88*, 307–317.

Dong, J., Feldmann, G., Huang, J., Wu, S., Zhang, N., Comerford, S.A., Gayyed, M.F., Anders, R.A., Maitra, A., and Pan, D. (2007). Elucidation of a universal size-control mechanism in Drosophila and mammals. Cell *130*, 1120–1133.

Dyson, N., Howley, P.M., Münger, K., and Harlow, E. (1989). The human papilloma virus-16 E7 oncoprotein is able to bind to the retinoblastoma gene product. Science *243*, 934–937.

Gatza, M.L., Chandhasin, C., Ducu, R.I., and Marriott, S.J. (2005). Impact of transforming viruses on cellular mutagenesis, genome stability, and cellular transformation. Environ. Mol. Mutagen. *45*, 304–325.

Gomez, M., Gomez, V., and Hergovich, A. (2014). The Hippo pathway in disease and therapy: cancer and beyond. Clin. Transl. Med. *3*, 22.

Grove, J., and Marsh, M. (2011). The cell biology of receptor-mediated virus entry. J. Cell Biol. *195*, 1071–1082.

He, C., Lv, X., Hua, G., Lele, S.M., Remmenga, S., Dong, J., Davis, J.S., and Wang, C. (2015a). YAP forms autocrine loops with the ERBB pathway to regulate ovarian cancer initiation and progression. Oncogene *34*, 6040–6054.

He, C., Mao, D., Hua, G., Lv, X., Chen, X., Angeletti, P.C., Dong, J., Remmenga, S.W., Rodabaugh, K.J., Zhou, J., et al. (2015b). The Hippo/YAP pathway interacts with EGFR signaling and HPV oncoproteins to regulate cervical cancer progression. EMBO Mol. Med. *7*, 1426–1449.

Herber, R., Liem, A., Pitot, H., and Lambert, P.F. (1996). Squamous epithelial hyperplasia and carcinoma in mice transgenic for the human papillomavirus type 16 E7 oncogene. J. Virol. *70*, 1873–1881.

Howlader, N., Noone, A.M., Krapcho, M., Neyman, N., Aminou, R., Waldron, W., Altekruse, S.F., Kosary, C.L., Ruhl, J., Tatalovich, Z., et al., eds. (2012). SEER cancer statistics review, 1975-2009 (vintage 2009 populations). National Cancer Institute, https://seer.cancer.gov/archive/csr/1975_2009_pops09/.

Howlader, N., Noone, A.M., Krapcho, M., Miller, D., Bishop, K., Kosary, C.L., Yu, M., Ruhl, J., Tatalovich, Z., Mariotto, A., et al., eds. (2018). SEER cancer statistics review, 1975-2014. National Cancer Institute, https://seer.cancer.gov/csr/1975_2014/.

Hua, G., He, C., Lv, X., Fan, L., Wang, C., Remmenga, S.W., Rodabaugh, K.J., Yang, L., Lele, S.M., Yang, P., et al. (2016). The four and a half LIM domains 2 (FHL2) regulates ovarian granulosa cell tumor progression via controlling AKT1 transcription. Cell Death Dis. *7*, e2297.

Koutsky, L. (1997). Epidemiology of genital human papillomavirus infection. Am. J. Med. *102* (*5A*), 3–8.

Kulasingam, S.L., Hughes, J.P., Kiviat, N.B., Mao, C., Weiss, N.S., Kuypers, J.M., and Koutsky, L.A. (2002). Evaluation of human papillomavirus testing in primary screening for cervical abnormalities: comparison of sensitivity, specificity, and frequency of referral. JAMA *288*, 1749–1757.

Lee, K.P., Lee, J.H., Kim, T.S., Kim, T.H., Park, H.D., Byun, J.S., Kim, M.C., Jeong, W.I., Calvisi, D.F., Kim, J.M., and Lim, D.S. (2010). The Hippo-Salvador pathway restrains hepatic oval cell proliferation, liver size, and liver tumorigenesis. Proc. Natl. Acad. Sci. U S A *107*, 8248–8253.

Liu, B., Zheng, Y., Yin, F., Yu, J., Silverman, N., and Pan, D. (2016). Toll receptor-mediated Hippo signaling controls innate immunity in Drosophila. Cell *164*, 406–419.

Liu, X.L., Ding, J., and Meng, L.H. (2018). Oncogene-induced senescence: a double edged sword in cancer. Acta Pharmacol. Sin. *39*, 1553–1558.

Lv, X., He, C., Huang, C., Hua, G., Wang, Z., Remmenga, S.W., Rodabough, K.J., Karpf, A.R., Dong, J., Davis, J.S., and Wang, C. (2017). G-1 inhibits breast cancer cell growth via targeting colchicine-binding site of tubulin to interfere with microtubule assembly. Mol. Cancer Ther. *16*, 1080–1091.

Lyng, H., Beigi, M., Svendsrud, D.H., Brustugun, O.T., Stokke, T., Kristensen, G.B., Sundfør, K., Skjønsberg, A., and De Angelis, P.M. (2004). Intratumor chromosomal heterogeneity in advanced carcinomas of the uterine cervix. Int. J. Cancer *111*, 358–366.

McNab, F., Mayer-Barber, K., Sher, A., Wack, A., and O'Garra, A. (2015). Type I interferons in infectious disease. Nat. Rev. Immunol. *15*, 87–103.

Mo, J.S., Park, H.W., and Guan, K.L. (2014). The Hippo signaling pathway in stem cell biology and cancer. EMBO Rep. *15*, 642–656.

National Cancer Institute (2018). HPV and cancer, https://www.cancer.gov/about-cancer/causes-prevention/risk/infectious-agents/hpv-fact-sheet.

Nguyen, H., Rendl, M., and Fuchs, E. (2006). Tcf3 governs stem cell features and represses cell fate determination in skin. Cell *127*, 171–183.

Pan, D. (2010). The Hippo signaling pathway in development and cancer. Dev. Cell *19*, 491–505.

Pannone, G., Bufo, P., Pace, M., Lepore, S., Russo, G.M., Rubini, C., Franco, R., Aquino, G., Santoro, A., Campisi, G., et al. (2016). TLR4 down-regulation identifies high risk HPV infection and integration in head and neck squamous cell carcinomas. Front. Biosci. (Elite Ed.) *8*, 15–28.

Perez-Plasencia, C., Duenas-Gonzalez, A., and Alatorre-Tavera, B. (2008). Second hit in cervical carcinogenesis process: involvement of wnt/beta catenin pathway. Int. Arch. Med. *1*, 10.

Raff, A.B., Woodham, A.W., Raff, L.M., Skeate, J.G., Yan, L., Da Silva, D.M., Schelhaas, M., and Kast, W.M. (2013). The evolving field of human papillomavirus receptor research: a review of binding and entry. J. Virol. *87*, 6062–6072.

Riley, R.R., Duensing, S., Brake, T., Münger, K., Lambert, P.F., and Arbeit, J.M. (2003). Dissection of human papillomavirus E6 and E7 function in transgenic mouse models of cervical carcinogenesis. Cancer Res. *63*, 4862–4871.

Roberts, J.N., Buck, C.B., Thompson, C.D., Kines, R., Bernardo, M., Choyke, P.L., Lowy, D.R., and Schiller, J.T. (2007). Genital transmission of HPV in a mouse model is potentiated by nonoxynol-9 and inhibited by carrageenan. Nat. Med. *13*, 857–861.

Roden, R., and Wu, T.C. (2006). How will HPV vaccines affect cervical cancer? Nat. Rev. Cancer *6*, 753–763.

Sankaranarayanan, R. (2015). HPV vaccination: the most pragmatic cervical cancer primary prevention strategy. Int. J. Gynaecol. Obstet. *131* (*Suppl 1*), S33–S35.

Scheffner, M., Werness, B.A., Huibregtse, J.M., Levine, A.J., and Howley, P.M. (1990). The E6 oncoprotein encoded by human papillomavirus types 16 and 18 promotes the degradation of p53. Cell *63*, 1129–1136.

Schiffman, M.H., and Castle, P. (2003). Epidemiologic studies of a necessary causal risk factor: human papillomavirus infection and cervical neoplasia. J. Natl. Cancer Inst. *95*, E2.

Schneider, W.M., Chevillotte, M.D., and Rice, C.M. (2014). Interferon-stimulated genes: a complex web of host defenses. Annu. Rev. Immunol. *32*, 513–545.

Scott, M., Nakagawa, M., and Moscicki, A.B. (2001). Cell-mediated immune response to human papillomavirus infection. Clin. Diagn. Lab. Immunol. *8*, 209–220.

Song, S., Pitot, H.C., and Lambert, P.F. (1999). The human papillomavirus type 16 E6 gene alone is sufficient to induce carcinomas in transgenic animals. J. Virol. *73*, 5887–5893.

Stanley, M.A. (2012). Epithelial cell responses to infection with human papillomavirus. Clin. Microbiol. Rev. *25*, 215–222.

Steinhardt, A.A., Gayyed, M.F., Klein, A.P., Dong, J., Maitra, A., Pan, D., Montgomery, E.A., and Anders, R.A. (2008). Expression of Yes-associated protein in common solid tumors. Hum. Pathol. *39*, 1582–1589.

Surviladze, Z., Dziduszko, A., and Ozbun, M.A. (2012). Essential roles for soluble virion-associated heparan sulfonated proteoglycans and growth factors in human papillomavirus infections. PLoS Pathog. *8*, e1002519.

Takeuchi, O., and Akira, S. (2009). Innate immunity to virus infection. Immunol. Rev. *227*, 75–86.

Tartey, S., and Takeuchi, O. (2017). Pathogen recognition and Toll-like receptor targeted therapeutics in innate immune cells. Int. Rev. Immunol. *36*, 57–73.

Torre, L.A., Bray, F., Siegel, R.L., Ferlay, J., Lortet-Tieulent, J., and Jemal, A. (2015). Global cancer statistics, 2012. CA Cancer J. Clin. *65*, 87–108.

Uren, A., Fallen, S., Yuan, H., Usubütün, A., Küçükali, T., Schlegel, R., and Toretsky, J.A. (2005). Activation of the canonical Wnt pathway during genital keratinocyte transformation: a model for cervical cancer progression. Cancer Res. *65*, 6199–6206.

Walboomers, J.M., Jacobs, M.V., Manos, M.M., Bosch, F.X., Kummer, J.A., Shah, K.V., Snijders, P.J., Peto, J., Meijer, C.J., and Muñoz, N. (1999). Human papillomavirus is a necessary cause of invasive cervical cancer worldwide. J. Pathol. *189*, 12–19.

Wang, S., Xie, F., Chu, F., Zhang, Z., Yang, B., Dai, T., Gao, L., Wang, L., Ling, L., Jia, J., et al. (2017). YAP antagonizes innate antiviral immunity and is targeted for lysosomal degradation through IKKε-mediated phosphorylation. Nat. Immunol. *18*, 733–743.

Weaver, B.A. (2006). Epidemiology and natural history of genital human papillomavirus infection. J. Am. Osteopath. Assoc. *106* (*3, Suppl 1*), S2–S8.

Westrich, J.A., Warren, C.J., and Pyeon, D. (2017). Evasion of host immune defenses by human papillomavirus. Virus Res. *231*, 21–33.

White, E.A., Münger, K., and Howley, P.M. (2016). High-risk human papillomavirus E7 proteins target PTPN14 for degradation. MBio *7*, e01530–16.

Yoon, C.S., Kim, K.D., Park, S.N., and Cheong, S.W. (2001). Alpha(6) integrin is the main receptor of human papillomavirus type 16 VLP. Biochem. Biophys. Res. Commun. *283*, 668–673.

Yu, F.X., Zhao, B., and Guan, K.L. (2015). Hippo pathway in organ size control, tissue homeostasis, and cancer. Cell *163*, 811–828.

Zanconato, F., Cordenonsi, M., and Piccolo, S. (2016). YAP/TAZ at the roots of cancer. Cancer Cell *29*, 783–803.

Zhang, Q., Meng, F., Chen, S., Plouffe, S.W., Wu, S., Liu, S., Li, X., Zhou, R., Wang, J., Zhao, B., et al. (2017). Hippo signalling governs cytosolic nucleic acid sensing through YAP/TAZ-mediated TBK1 blockade. Nat. Cell Biol. *19*, 362–374.

STAR★METHODS

KEY RESOURCES TABLE

REAGENT or RESOURCE	SOURCE	IDENTIFIER
Antibodies		
Ki67	Abcam (Cambridge, MA)	ab15580
SDC1	Abcam (Cambridge, MA)	ab188861
p40(deltaNp63)	Ventana Medical Systems, Inc. (Tucson, AZ)	790-4950
P53	Santa Cruz Biotechnology Inc (Dallas, TX)	sc-126
ISG15	Santa Cruz Biotechnology Inc (Dallas, TX)	sc-166755
YAP	Cell Signaling Technology Inc. (Danvers, MA)	#4912
phospho-YAP (Ser127)	Cell Signaling Technology Inc. (Danvers, MA)	#4911
Pan KRT	Cell Signaling Technology Inc. (Danvers, MA)	#4545
EGFR	Cell Signaling Technology Inc. (Danvers, MA)	# 4267
ITGA6	Cell Signaling Technology Inc. (Danvers, MA)	#3750
TLR1	Cell Signaling Technology Inc. (Danvers, MA)	# 2209
TLR2	Cell Signaling Technology Inc. (Danvers, MA)	#1227
MYD88	Cell Signaling Technology Inc. (Danvers, MA)	#4283
TRIF	Cell Signaling Technology Inc. (Danvers, MA)	#4596
TBK1	Cell Signaling Technology Inc. (Danvers, MA)	#3504
NF-κB1 p105/p50	Cell Signaling Technology Inc. (Danvers, MA)	#12540
NF-κB2 p100/p52	Cell Signaling Technology Inc. (Danvers, MA)	#3017
JAK1	Cell Signaling Technology Inc. (Danvers, MA)	#3344
JAK2	Cell Signaling Technology Inc. (Danvers, MA)	#3230
STAT1	Cell Signaling Technology Inc. (Danvers, MA)	#9172
STAT2	Cell Signaling Technology Inc. (Danvers, MA)	#72604
IRF3	Cell Signaling Technology Inc. (Danvers, MA)	#11904
IRF9	Cell Signaling Technology Inc. (Danvers, MA)	#76684
phospho-STAT1	Cell Signaling Technology Inc. (Danvers, MA)	#7649
phospho-STAT2	Cell Signaling Technology Inc. (Danvers, MA)	#88410
β-actin	Sigma-Aldrich (St. Louis, MO)	#A5441
Bacterial and Virus Strains		
p16L1L2	Addgene	#45291
pCIneoEGFP	Addgene	#46949
MXIV-neo	Jixing Dong Lab	N/A
YAP-neo	Jixing Dong Lab	N/A
YAP^{S127A}-neo	Jixing Dong Lab	N/A
Biological Samples		
The human cervical tissues microarray	US Biomax, Inc	BB10011
	Panomics, Inc.	CIN481, 482, & 483
Chemicals, Peptides, and Recombinant Proteins		
Interferon alpha 2b	R&D systems Inc.	#11105-1
Critical Commercial Assays		
RNAeasy micro kit	QIAGEN	#74004
iScript Reverse Transcription Supermix for RT-qPCR	BIO-RAD	#1708841
iTaq Universal SYBR® Green Supermix	BIO-RAD	#1725121
VECTASTAIN® Elite® ABC HRP Kit (Peroxidase, Rabbit IgG)	VECTOR LABORATORIES	PK-6103
Antigen Unmasking Solution, Citric Acid Based	VECTOR LABORATORIES	H-3300
ImmPACT® DAB Peroxidase (HRP) Substrate	VECTOR LABORATORIES	SK-4105

(*Continued on next page*)

Continued

REAGENT or RESOURCE	SOURCE	IDENTIFIER
ImmPACT® NovaRED Peroxidase (HRP) Substrate	VECTOR LABORATORIES	SK-4805
VECTASTAIN® ABC-AP Staining KIT (Alkaline Phosphatase, Rabbit IgG)	VECTOR LABORATORIES	AK-5001
BLOXALL® Endogenous Peroxidase and Alkaline Phosphatase Blocking Solution	VECTOR LABORATORIES	SP-6000
VECTOR® Red Alkaline Phosphatase (Red AP) Substrate Kit	VECTOR LABORATORIES	SK-5100
Experimental Models: Cell Lines		
Primary hCerECs	ScienCell Research Laboratories	#7060
Ect1/E6E7 cells	ATCC	CRL-2614
SiHa	ATCC	HTB-35
Experimental Models: Organisms/Strains		
KRT14-rtTA Mice (FVB-Tg(KRT14-rtTA) F42Efu/J)	The Jackson Laboratory	#008099
TRE-YAP^{S127A} mice	Dr. Fernando Camargo's lab	N/A
KRT14-E6 mice	Dr. Lambert's laboratory (Herber et al., 1996; Song et al., 1999)	N/A
KRT14-E7 mice	Dr. Lambert's laboratory (Herber et al., 1996; Song et al., 1999)	N/A
Oligonucleotides		
Primers for qRT-PCR, see Table S1	This Paper	N/A
Primers for genotyping, see Table S1	This Paper	N/A
siRNA (smart pool) targeting YAP1	Dharmacon	M-012200-00-0005
siRNA (#1) targeting YAP1	Dharmacon	D-012200-01-0002
siRNA (#2) targeting YAP1	Dharmacon	D-012200-02-0002
siRNA (smart pool) targeting ITGA6	Dharmacon	D-007214-01-0005
siRNA (#1) targeting ITGA6	Dharmacon	D-007214-01-0002
siRNA (#2) targeting ITGA6	Dharmacon	D-007214-02-0002

CONTACT FOR REAGENT AND RESOURCE SHARING

Further information and requests for reagents may be directed to, and will be fulfilled by the Lead Contact, Cheng Wang (cwang34@mgh.harvard.edu).

EXPERIMENTAL MODEL AND SUBJECT DETAILS

Mouse Model Studies

Mouse model handling and animal experimental procedures were approved by the Institutional Animal Care and Use Committee (IACUC) of the University at Nebraska Medical Center (UNMC) and Massachusetts General Hospital (MGH). KRT14-rtTA (FVB-Tg(KRT14-rtTA) F42Efu/J, #008099) mice were from The Jackson Laboratory. These mice express the reverse tetracycline-controlled transactivator (rtTA) protein in basal cells of stratified epithelium under control of the human keratin 14 (KRT14) gene promoter (Nguyen et al., 2006). The TRE-YAP^{S127A} mice were from Dr. Fernando Camargo's lab (Boston Children's hospital). These mice express a constitutively active form of YAP1 protein (YAP^{S127A}) under the control of a tetracycline regulatory element (TRE) (Camargo et al., 2007). The KRT14-E6 and KRT14-E7 mice, which express HPV16 E6 and HPV16 E7 protein under control of KRT14 promoter, respectively, were from Dr. Lambert's laboratory (Herber et al., 1996; Song et al., 1999). Mouse breeding schemes were shown in Figures 1A and 6A. Genotyping was performed using RT-PCR technique and tail tissue. The genomic DNA in tails was extracted using DirectPCR Lysis Reagent (#102-T) from Viagen Biotech Inc. (Los Angeles, CA). Briefly, tail tissue was lysed in a single-tube with 250μl DirectPCR Lysis and 5μl 20mg/ml Proteinase K (Sigma, #p6556) at 55°C for 24 hours followed by incubating at 85°C for 1 hour. After centrifuging, clear lysate (0.75 μl) was directly used for 20 μl PCR reaction. Primers for genotyping and expected size of each gene are listed in Table S1. Female mice were used for cervical cancer modeling. All control, KRT-14-YAP^{S127A}, KRT14-E6/E7, and KRT14-E6/E7-YAP^{S127A} transgenic mice were treated with 0.05 mg/mL Dox in drinking water (long-term treatment) or 2 mg/mL Dox in drinking water (2-week short-term treatment) to induce the expression of transgenes when they were three months old. Mice were euthanized and recorded as dead if they showed serious illnesses (dysuria, wasting, or sluggishness)

and incurable. Euthanasia was recommended by an experienced veterinarian in the animal core facility if tumor-carrying mice were incurable and suffering more than moderate stress. Mouse tissues were collected and processed for preparation of protein, RNA, frozen and paraffin sections after euthanasia.

Cell Culture

Primary hCerECs (hCerEC, #7060) were purchased from ScienCell Research Laboratories, Inc. (Carlsbad, CA). The ectocervical Ect1/E6E7 cells (ECT, #CRL-2614) and SiHa cervical cancer cell line (SiHa, #HTB-35) were from ATCC (Manassas, VA). Cells were cultured according to instructions provided by ScienCell Research Laboratory and ATCC. All cell lines were validated by short tandem repeat (STR) polymorphism analysis performed by the Genetica DNA Laboratories (Burlington, NC, USA). All cells were maintained in a humidified atmosphere at 37°C and 5% CO2.

Human Cervical Tissue Microarray

The human cervical tissues microarray (with normal control, CINs and cancerous tissues with stage and grade information) was purchased from US Biomax (Rockville, MD) and Panomics Inc (Richmond, CA). The use of archived human tissue was approved by Institutional Review Board (IRB) of University of Nebraska Medical Center.

METHOD DETAILS

Reagents and Materials

Interferon alpha 2b (IFNα2b, #11105-1) was purchased from R&D systems Inc. (Minneapolis, MN). DMEM/F12 culture media (#SH-300-2301LR) were from Fisher Scientific. Ultroser Serum Substitute (#15950-017) was purchased from Pall Corporation (Port Washington, NY). iScript Reverse Transcription Supermix (#1708841) and iTaq Universal SYBR® Green Supermix (#1725121) were purchased from Bio-Rad Laboratories, Inc. (Hercules, CA); RNeasy Mini Kit (#74106) was purchased from QIAGEN Inc. (Valencia, CA). YAP siRNAs and ITGA6 siRNAs were purchased from Dhamarcon/Thermo Scientific (Pittsburgh, PA). The SuperSignal West Femto Chemiluminescent Substrate Kit (#34095) for western blotting was from Pierce/Thermo Scientific (Rockford, IL); PVDF transfer membrane (IPVH00010) was from Merck Millipore Ltd (Darmstadt, Germany). Other General Chemical Reagents were purchased from Sigma (St. Louis, MO), Fisher (Pittsburgh, PA), or United States Biochemical (Cleveland, OH). Information of antibodies (antibody name, catalog numbers, and sources) used in this study is listed in key resources table.

IHC

Expression of YAP1 (in Figures 1, 2, S2, and S4), Ki67, deltaNp63, p16INK4A, Pan KRT, EGFR, P53, YAP1, ITGA6, and SDC1 in human or/and mouse cervical tissues were detected using peroxidase-based IHC kits (VECTASTAIN ELITE ABC KITS, #PK-6101 for Ribbit IgG, #PK-6102 for Mouse IgG, Vector Laboratories, Burlingame, CA]. Mouse cervical tissues were deparaffinized with xylene and then rehydrated with gradient ethanol in ddH$_2$O. Antigens were retrieved at 115°C with a pressure cooker for 20 minutes using Citrate-based 1% unmasking solution (H-3300, Vector Laboratories, Burlingame, CA). Endogenous peroxidase activity was quenched with 1% hydrogen peroxide for 30 minutes. Slides were incubated with blocking buffer for 30 minutes before incubating with primary antibodies at 4°C for 16 hours. After washing with PBST for 3 × 5 minutes, slides were incubated with Biotinylated secondary antibody (provided in the Kit) at 22°C for 45 minutes. Antigens were visualized with VECTASTAIN ELITE ABC kits ((#PK-6101 for Ribbit IgG, #PK-6102 for Mouse IgG) and ImmPACT DAB kit (#SK4105, Vector Laboratories, Burlingame, CA) / ImmPACT NovaRED substrate kit (#AK-4800) (Vector Laboratories, Burlingame, CA) according to instructions provided in kits. The sections were also counterstained with hematoxylin (#H-3401, Vector Laboratories, Burlingame, CA) for 30 seconds to visualize nuclei.

Expression of YAP1 in mouse cervical tissues showed in Figure 2B was detected by alkaline phosphatase-based detection systems using VECTASTAIN® ABC-AP Staining Kit (Rabbit IgG, # AK-5001). The basic experimental steps of ABC-AP kit are similar with ELITE ABC system, except that the endogenous enzyme activity was blocked with BLOXALL Blocking Solution (#SP-6000, Vector Laboratories) and the signal was visualized using Vector® Red Substrate kit (#SK-5100) (Vector Laboratories, Burlingame, CA).

All stained sections were scanned with an iSCAN Coreo Slide Scanner (Ventana Medical Systems, Inc. Oro Valley, AZ). The signal intensity and positivity (the ratio of positive cell number relative to the total cell number) of immunosignal on each section were quantified using an Aperio ImageScope software (Leica Biosystems Imaging, Inc. Vista, CA).

Fluorescent Immunocytochemistry

The expression and cellular location of YAP1, IRF3, IRF9, NFKB1, and STAT1 were analyzed by fluorescent immunocytochemistry as described previously (Lv et al., 2017). Cells were grown on coverslips in a 24-well plate. After treatment, cells were fixed with 4% formaldehyde (in 1X PBS) at 4°C for 20 minutes, rinsed with PBST for 3 times X 5 minutes, blocked cells with normal donkey serum (10%) for 1 hour before incubating with primary antibody (~200x) at 4°C for 16 hours. Antigen was visualized with fluorochrome-conjugated secondary antibodies. Fluorescence-conjugated secondary antibodies for immunofluorescent analyses, including Alexa Fluor® 488 AffiniPure Donkey Anti-Rabbit IgG (#711-545-152) and Alexa Fluor® 488 AffiniPure Donkey Anti-Mouse IgG (#715-545-150), were from the Jackson Immunoresearch Laboratories Inc. (West Grove, PA); Rhodamine Phalloidin (#R415) for visualizing actin was from Thermo Fisher Scientific (Rockford, IL). Nuclei were stained with DAPI. Images were captured using a

ZEISS Xradia 810 Ultra Confocal Laser Scanning Microscope and analyzed with Zeiss Zen 2012 software (Carl Zeiss Microscopy, LLC, Thornwood, NY).

Western Blot Analysis

Western blot was used to determine the relative protein levels in cells and tissues with a protocol described previously with minor modification (Hua et al., 2016). Cells were harvested and lysed (150μl/10^6 cells) on ice. Protein concentration was measured using BCA assay kit. Thirty micrograms protein per sample was loaded onto the SDS-PAGE gel, fractioned using a Bio-Rad electrophoresis system, and then transferred from gel onto PVDF membranes. The protein containing membrane was blocked with 5% BSA for 60 minutes, incubated with primary antibodies at 4°C for 16 hours and then probed with a HRP-linked secondary antibody (#7074 for Rabbit IgG, #7076 for Mouse IgG, Cell Signaling Technology, Inc. Danvers, MA). The immunosignal was visualized with a SuperSignal West Femto Maximum Sensitivity Substrate Kit (#34096, Thermo Fisher Scientific, Waltham, MA). Images were captured and analyzed using a UVP gel documentation system (UVP, Upland, CA).

Quantitative Real Time-PCR

Total RNA from cultured human cells and mouse cervical tissues were extracted using TRIzol reagent (Invitrogen; Carlsbad, CA) and QIAGEN RNeasy mini kit (#74106QIAGEN, Carlsbad, CA) following instructions provided by the manufacture. cDNA was synthesize using an iScript Reverse Transcription Supermix for RT-qPCR Kit (Bio-Rad Laboratories, Inc.). qT-PCR was performed with the Bio-Rad CFX96 real-time PCR system using an iTaq Universal SYBR® Green Supermix Kit (Bio-Rad Laboratories, Inc.). *GAPDH* and 18*S* were used as internal references. All primer sequences are shown in Table S1.

YAP1 Overexpression

Primary hCerECs were cultured following a protocol provided by the vendor (Catalog #7060, ScienCell Research Laboratories, Inc.). SiHa (a human cervical cancer cell line contained HPV16) and ECT1 (a human endocervical cell line immortalized by HPV16 E6/E7) cells were cultured with 2% Ultroser G serum substitute (Pall Corporation) in DMEM/F12 medium. hCerEC-MX and ECT1-MX cells was generated by transfecting hCerEC and ECT1 cells with retrovirus-based empty MXIV vector (MX) as control; hCerEC-YAP and ECT1-YAP cells were generated by transfecting hCerEC and ECT1 cells with vectors expressing wild-type of YAP1 protein; hCerEC-YAP^{S127A} and ECT1-YAP^{S127A} cells were generated by transfecting hCerEC and ECT1 cells with vectors expressing constitutively active YAP1 (YAP^{S127A}, Serine at residue 127 is replaced by an Alanine resulting in the constitutive activation of YAP1). Since hCerEC are primary cells, all experiments using hCerEC-MX, hCerEC-YAP and hCerEC-YAP^{S127A} cells were performed within 4 passages after transfection. Expression of *YAP1* gene was confirmed by both RT-PCR and western blot assay.

Gene Silencing by siRNA

For gene silencing experiments, hCerEC, ECT1, or SiHa cells were transfected with scrambled control siRNA (siGENOME Non-Targeting siRNA #1, D-001210-01-20) or *YAP1* specific siRNAs (siYAP), or *ITGA6* specific siRNAs (siITGA6) for 6 hours using Lipofectamine 2000 protocol (Invitrogen, Carlsbad, CA) after cells reaching 60% confluent. siRNAs targeting *YAP1* and *ITGA6* genes were from Dharmacon (Lafayette, CO). At least two different siRNAs for each gene were used to validate the knockdown efficiency of *ITGA6* specific siRNAs. Gene knockdown was detected by RT-PCR and western blot. The results for validation of *YAP1* siRNAs were presented in supplemental Figures S12A–S12C. The results for validation of *ITGA6* siRNAs were presented in supplemental Figures S12D–S12E.

Preparation of HPV PsVs

HPV PsVs (HPV16- PsV) is a HPV16 virion-like particle in which HPV16 L1 and L2 proteins encapsulate a GFP plasmid (reporter gene). Infection efficiency of HPV16 is indicated by the signal intensity of GFP and the ratio of GFP-positive cells in HPV16-PsV treated cells. Previous studies have demonstrated that this PsV could well mimic HPV infection process (Conway and Meyers, 2009; Roberts et al., 2007). HPV16-PsV were generated as described previously[13,59]. Briefly, plasmids expressing HPV capsid proteins L1 and L2 (p16L1L2, #45291, Addgene. Donated by Dr. John T. Schiller's Lab) and GFP reporter (pCIneoEGFP, #46949, Addgene. Donated by Dr. John T. Schiller's Lab) were co-transfected into 293TT cells using a Lipofectamine 2000 (#11668027, Invitrogen) protocol. After 48 hours, the transfected cells were trypsinized, washed with DPBS-Mg solution for three times, and re-suspend with a cell concentration of 1×10^8 cells/ml in DPBS-Mg solution (with 0.5% Brij58, 0.2% Benzonase, and 0.2% Plasmid Safe). Cell lysate was incubated at 37°C for 24 hours to ensure capsid maturation and then chilled on ice for 10 minutes. After chilling on ice, the salt concentration of the cell lysate was adjusted to 850 mM NaCl and incubated on ice for 10 more minutes. The lysate was then clarified by centrifugation, and the supernatant was layered onto an Optiprep gradient. The gradient was spun for 4.5 hours at 16°C at 40,000 RPM in a SW40 rotor (Beckman Coulter, Inc., Brea, CA). The concentration of HPV PsVs was evaluated by analysis of GFP transduction efficiency in 293 cells.

HPV16 PsV Infection of Cells

hCerEC, ECT1 or SiHa Cells (2×10^4 cells / well) were seeded in a 12-well plate and cultured overnight. HPV16 PsVs (1.0 or 2.0 MOI) were added into culture medium and incubated for 72 hours. GFP signal was captured using a Nikon-ECLIPSE NI with X-CITE system. The images were analyzed using Zeiss Zen 2010 software (Carl Zeiss Microscopy, Thornwood, NY).

QUANTIFICATION AND STATISTICAL ANALYSIS

All experiments were repeated at least four times unless otherwise noted. Data are presented as mean ± SEM. Statistical analysis was conducted, and graphs were made with GraphPad Prism 7.04 (GraphPad Software, Inc. La Jolla, CA). Data were analyzed for significance using Student's t test (two groups) or one-way ANOVA with Tukey's post hoc tests (multiple groups). A value of $p < 0.05$ was considered statistically significant.

Cell Reports, Volume *26*

Supplemental Information

A Human Papillomavirus-Independent Cervical Cancer Animal Model Reveals Unconventional Mechanisms of Cervical Carcinogenesis

Chunbo He, Xiangmin Lv, Cong Huang, Peter C. Angeletti, Guohua Hua, Jixin Dong, Jin Zhou, Zhengfeng Wang, Bowen Ma, Xingcheng Chen, Paul F. Lambert, Bo R. Rueda, John S. Davis, and Cheng Wang

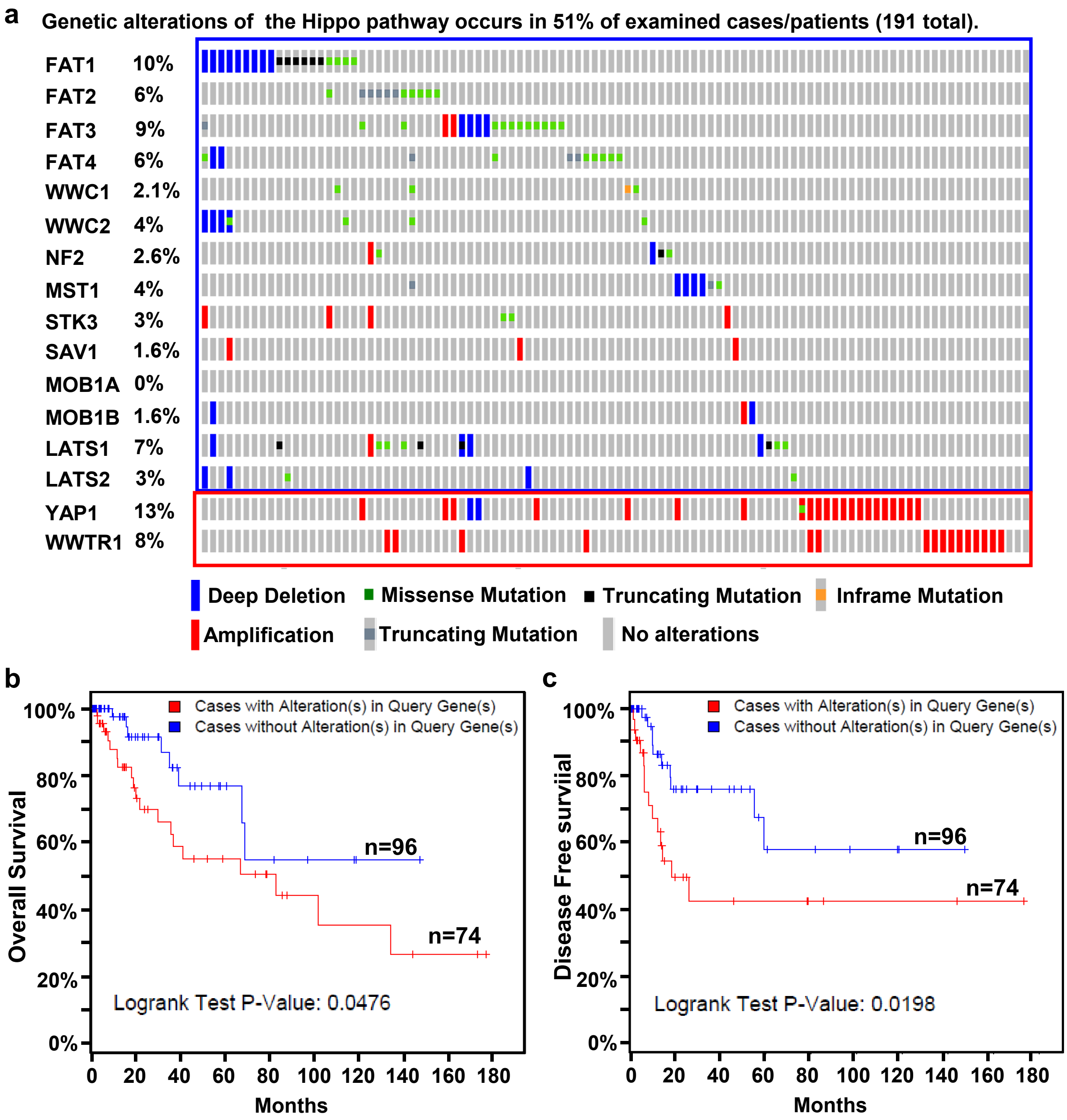

Figure S1. Alteration of genes encoding the key components of the Hippo pathway in cervical cancer patients. Related to Figure 1. a) Deletion and mutation of the upstream tumor suppressive genes (*MST1, LATS1/2*, and *FAT1/2/3/4*, *etc.*) and amplification of the downstream tumorigenic effectors (*YAP1* & *WWTR1*) of the Hippo pathway are frequently (51%) observed in 191 sequenced cervical cancer patient samples. Data were extracted from TCGA datasets with permission and analyzed using cBioPortal online tools (http://www.cbioportal.org/). **b & c)** Kaplan-Meier curves showing correlations between overall survival (**b**) / disease free survial (**C**) and genetic alteration of the Hippo/YAP pathway. The TCGA patient samples with survival information were stratified into two groups based on the genetic alteration of the Hippo signaling pathway: cases/patients with amplification of *YAP1* or deletion / mutation of tumor suppressors (*FAT1/2/3/4*, *MST1*, *LATS1/2*) in query genes (blue line, n=96) and cases/patients without genetic alterations in query genes (red line, n=74). Please note that patients with genetic alterations of the Hippo/ YAP pathway have poorer outcomes.

Supplementary Figure S1

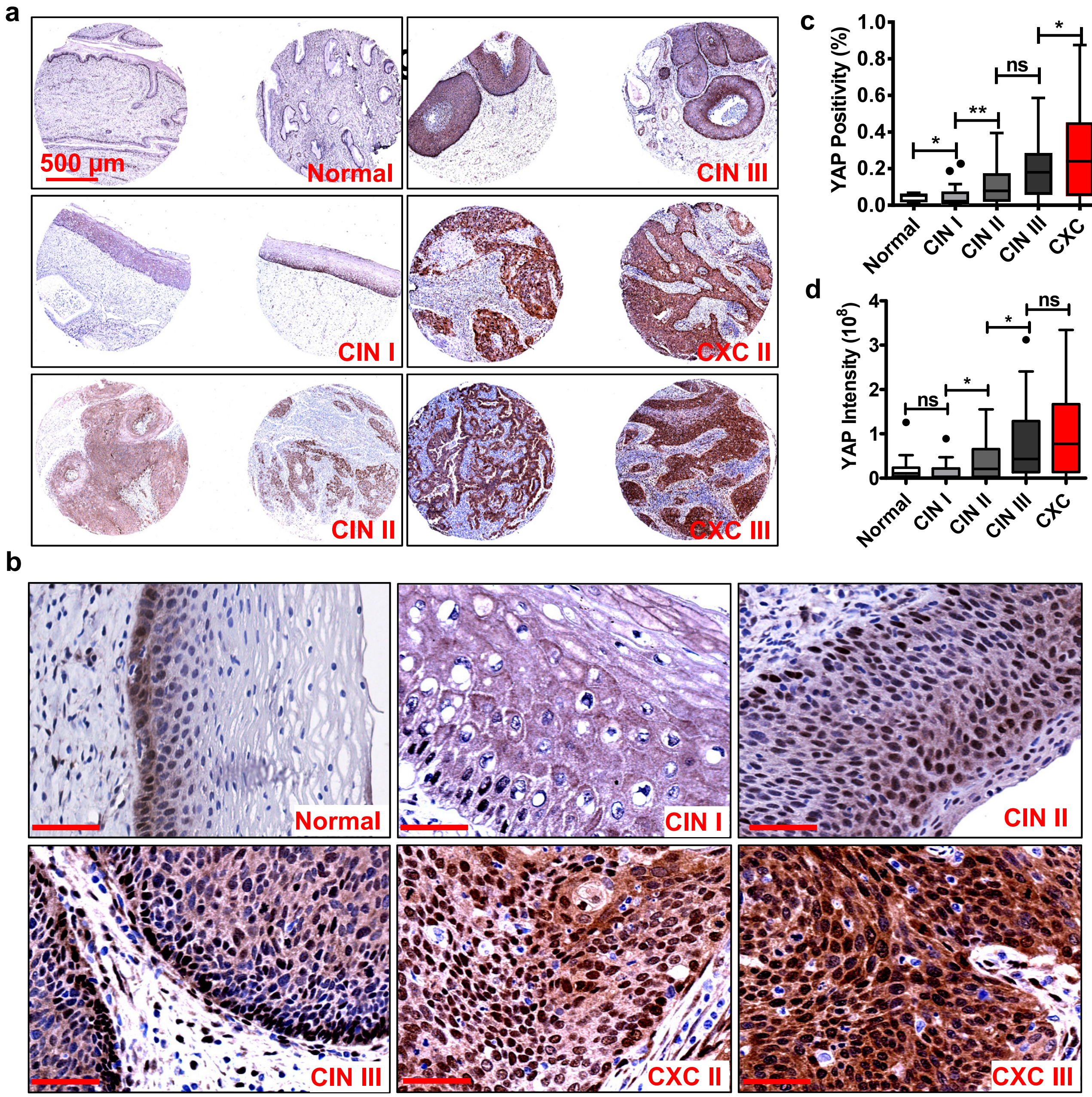

Figure S2. YAP1 expression is associated with cervical cancer initiation and progression. Related to Figure 1. **a)** Representative images showing the expression of YAP1 in normal cervical tissues, low and high grade cervical intraepithelial neoplasia (CIN), early-stage cervical cancer (II), and advanced-stage cervical cancer (stage III) tissues. Note the significant increase of YAP1 immunosignals (brown staining) with cervical cancer progression. Scale bar: 0.5 mm. **b**) High resolution images showing the relative level and cellular location of YAP1 protein in tissues derived from age-matched normal control, different stages of CIN and invasive cancer patients. Scale bar: 50 µm. **c & d)** The positivity (**C,** percentage of positively stained cells relative to the total number of cells in the tissue section) and intensity (**d**) of YAP1 immunosignals in the normal, CIN, and cancerous tissues. Data were analyzed using One-way ANOVA with Tukey's multiple comparisons post-test. Bars represent means ± SEM (n = 7 for normal tissues; n=28 for CIN I; n=23 for CIN II; n=29 for CIN III; n=68 for cervical cancer; *: $p < 0.05$; **: $p < 0.01$; ns: no significant difference).

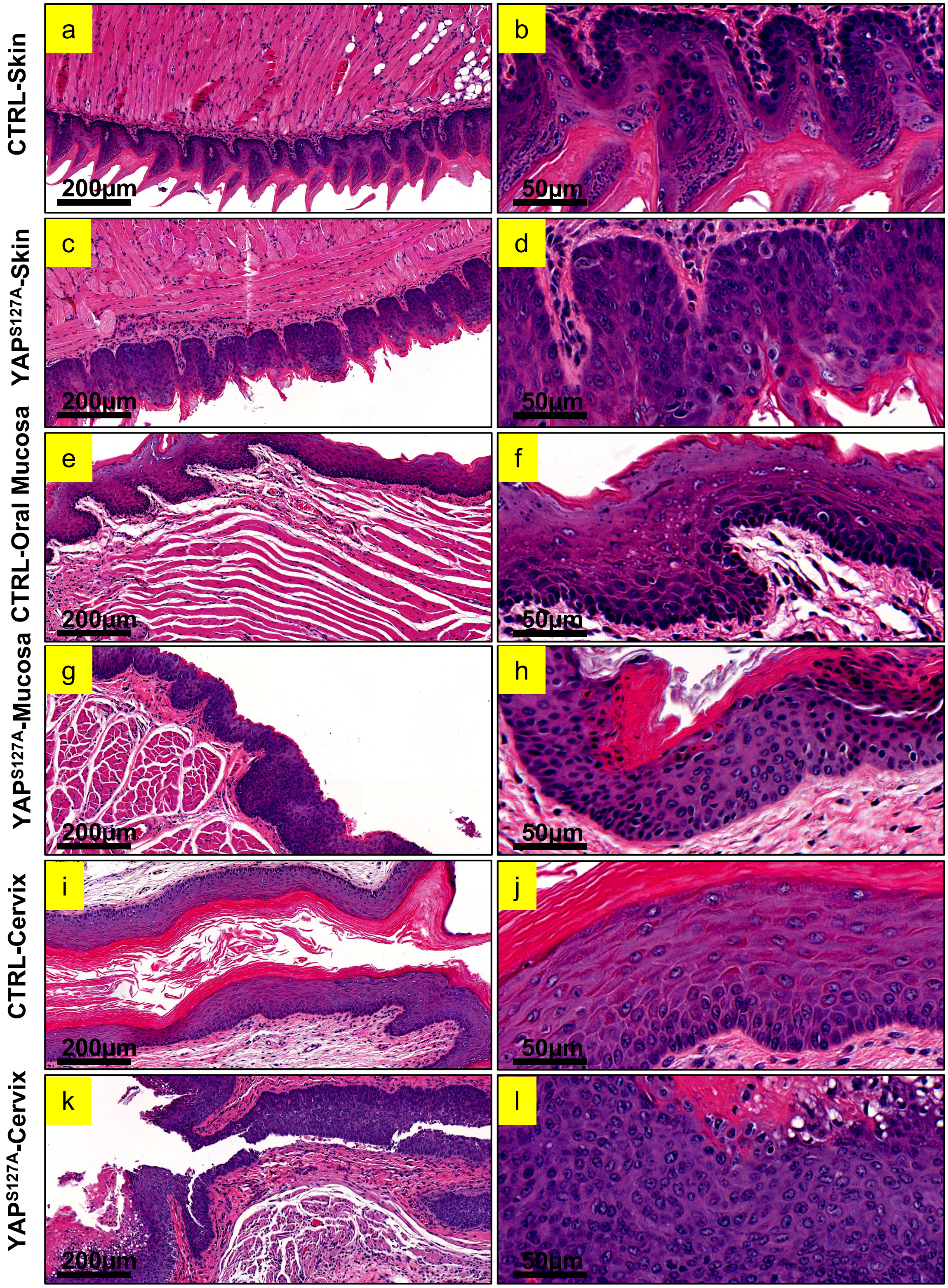

Figure S3. High concentration of Doxycycline induced epithelial neoplasia in KRT14-rtTA-TRE-YAP^{S127A} transgenic mice. Related to Figure 1. Representative images showing histology of epithelium in control and KRT14^{-rtTA}-$^{TRE-}$YAP^{S127A} transgenic mice treated with high concentration of doxycycline (2mg/ml in drink water). H&E staining showed that high concentration of Dox induced neoplasia not only in cervical epithelium, but also in epithelium of skin and oral mucosa of the KRT14-YAP^{S127A} transgenic mice.

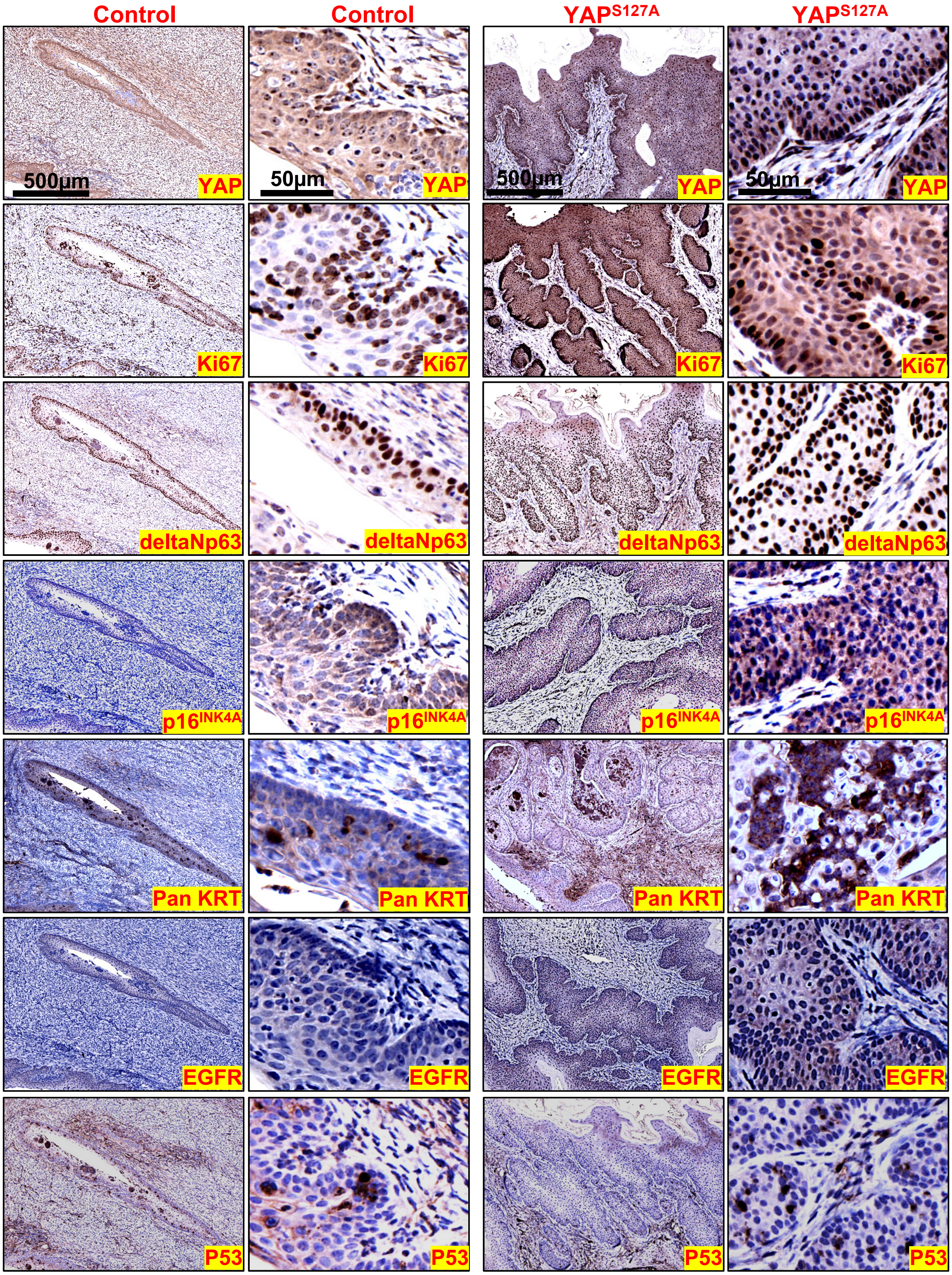

Figure S4. Molecular signature of Cervical Cancer derived from KRT14-YAP^{S127A} transgenic mice. Related to Figure 1. Representative images showing expressions of YAP1 and several known biomarkers (Ki67, deltaNp63, p16^{INK4A}, EGFR, and Pan KRT) for cervical squamous cell carcinoma (CVSCC) in tumor tissues derived from KRT14-YAP^{S127A} transgenic mice. Please note that all known markers are significantly accumulated in cervical tumor tissues derived from the KRT14-YAP^{S127A} transgenic mice. Please also note that p53 expression have no significant change. Scale bar: 500 µm in low magnification images; 50 µm in high resolution images.

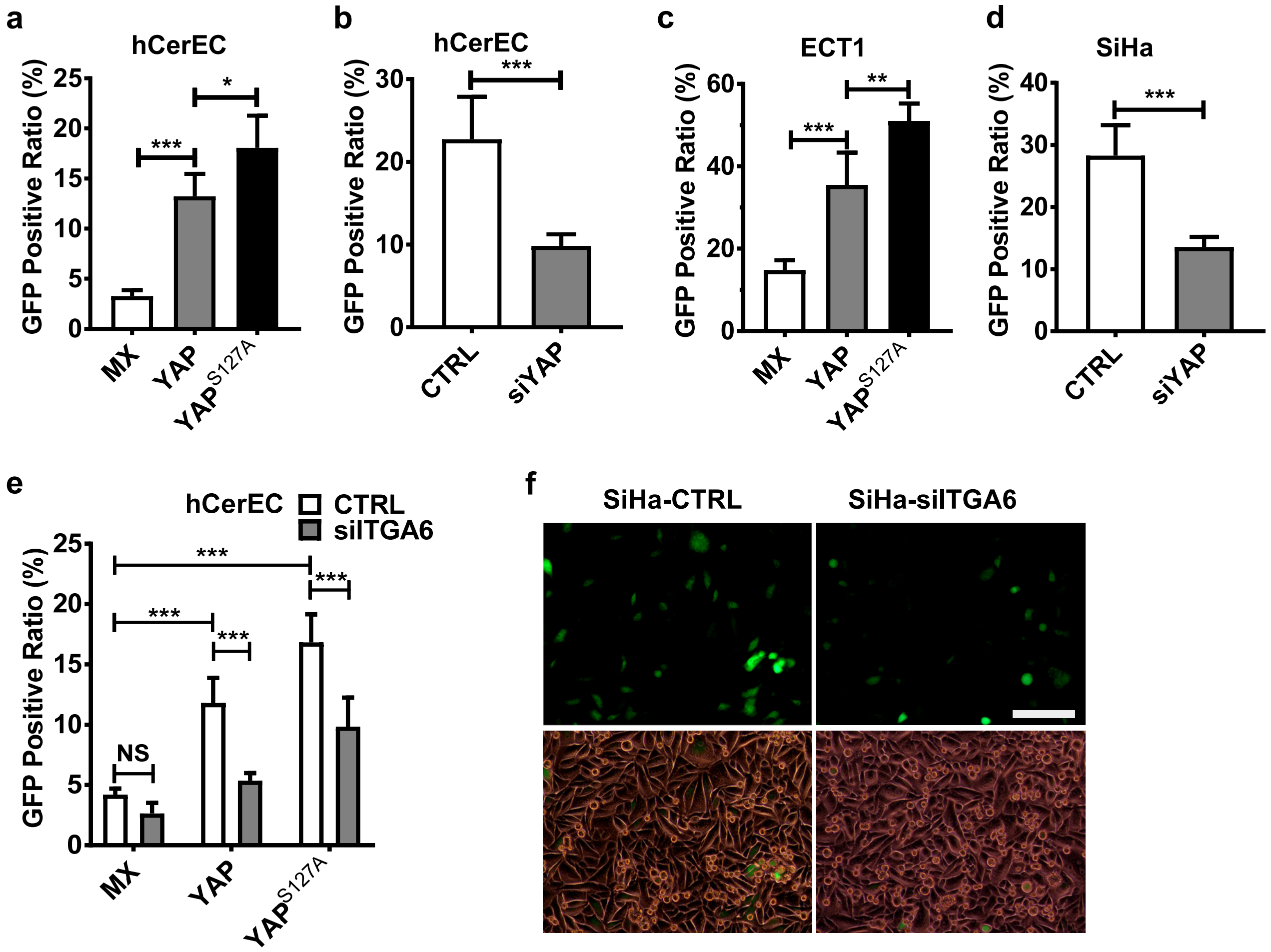

Figure S5. YAP1 regulates HPV infectivity via controlling the expression of the putative HPV receptors. Related to Figures 2 & 3. a) Quantitative data of Figure 2F showing the percentage of the GFP positive cells in hCerEc-MX (control), hCerEC-YAP, and hCerEC-YAP^{S127A} cells. **b)** Quantitative data of Figure 2G showing the percentage of the GFP positive cells in *YAP1*-knockdown hCerEC cells (siRNA, cells transfected with *YAP1* specific siRNA) and control hCerEC cells (CTRL, cells transfected with scramble siRNA). **c)** Quantitative data of Figure 2H showing the percentage of the GFP positive cells in ECT1-MX (control), ECT1-YAP, and ECT1-YAP^{S127A} cells. **d)** Quantitative data of Figure 2I showing the percentage of the GFP positive cells in YAP1-knockdown SiHa cells (siRNA, cells transfected with *YAP1* specific siRNA) and control SiHa cells (CTRL, cells transfected with scramble siRNA). Each bar represents the mean ± SEM (n = 5). *: $p < 0.05$; **: $p < 0.01$; ***: $p < 0.001$. Please note that HPV preferentially infects YAP1 highly-expressed cells. **e**) Quantitative results of figure 3C showing the ratio of GFP positive cells in hCerEC-MX, hCerEC-YAP, and hCerEC-YAP^{S127A} cells with (siITGA6) or without (CTRL) knockdown of ITGA6. Each bar represents mean ± SEM (n=5). NS, no significance; ***, $p < 0.001$. **f**) Representative images showing HPV16 PsV-derived GFP signal in SiHa-CTRL and SiHa-siITGA6 cells. Note that Knockdown of ITGA6 reduced HPV16 PsV-derived GFP signal in SiHa cells. Scale bar: 100 µm.

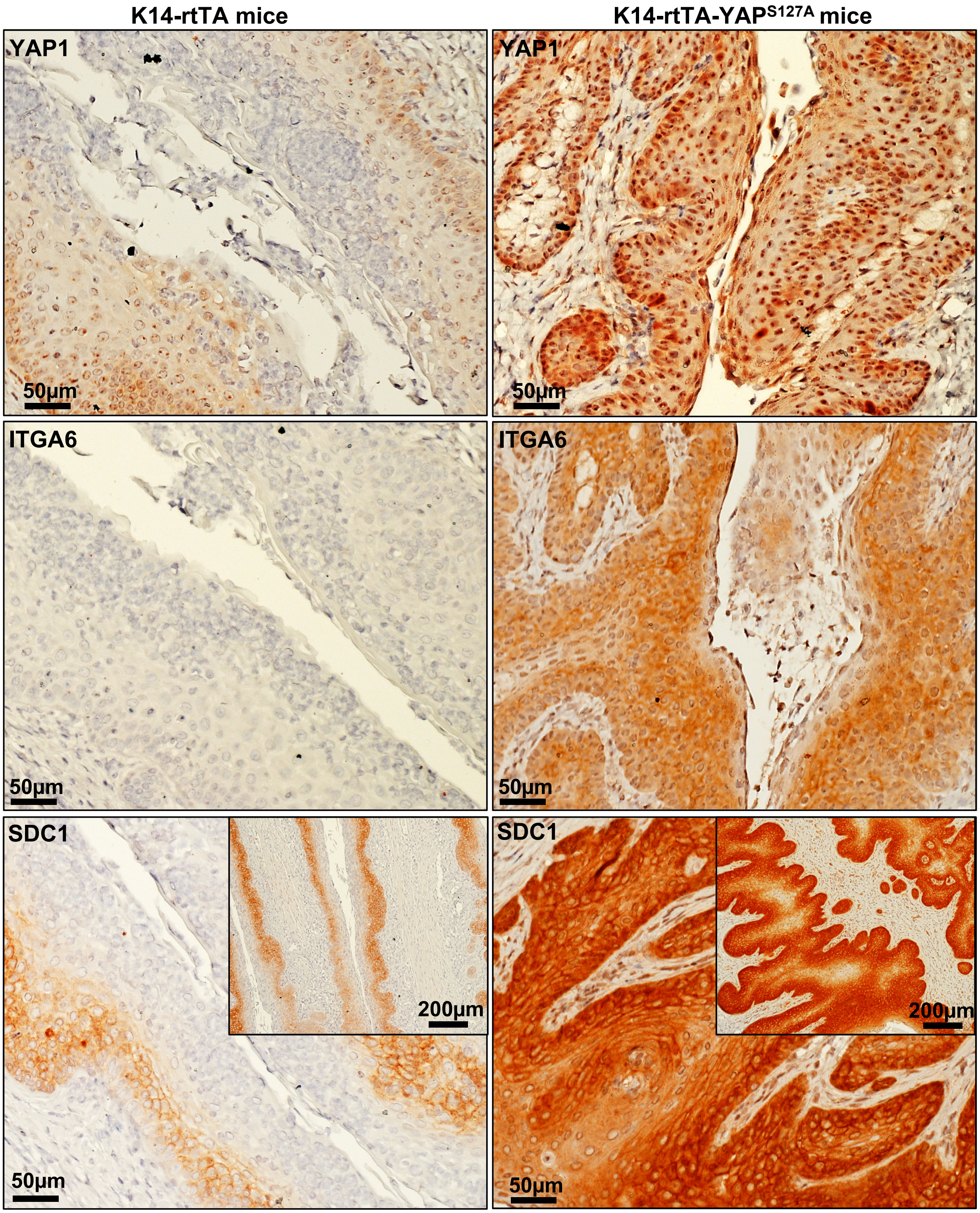

Figure S6. Upregulation of the putative HPV receptor molecules in cervical tissues of KRT14-YAP^{S127A} transgenic mice. Related to Figure 3. Representative images showing expressions of YAP1 and two well-studied putative HPV receptor molecules, SDC1 and ITGA6, in cervical tissues derived from 10-month old control (KRT-rtTA mice) and KRT14-YAP^{S127A} transgenic mice. Please note upregulation of SDC1 and ITGA6 proteins in the epithelium KRT14-YAP^{S127A} transgenic mice. Scale bar: 50µm.

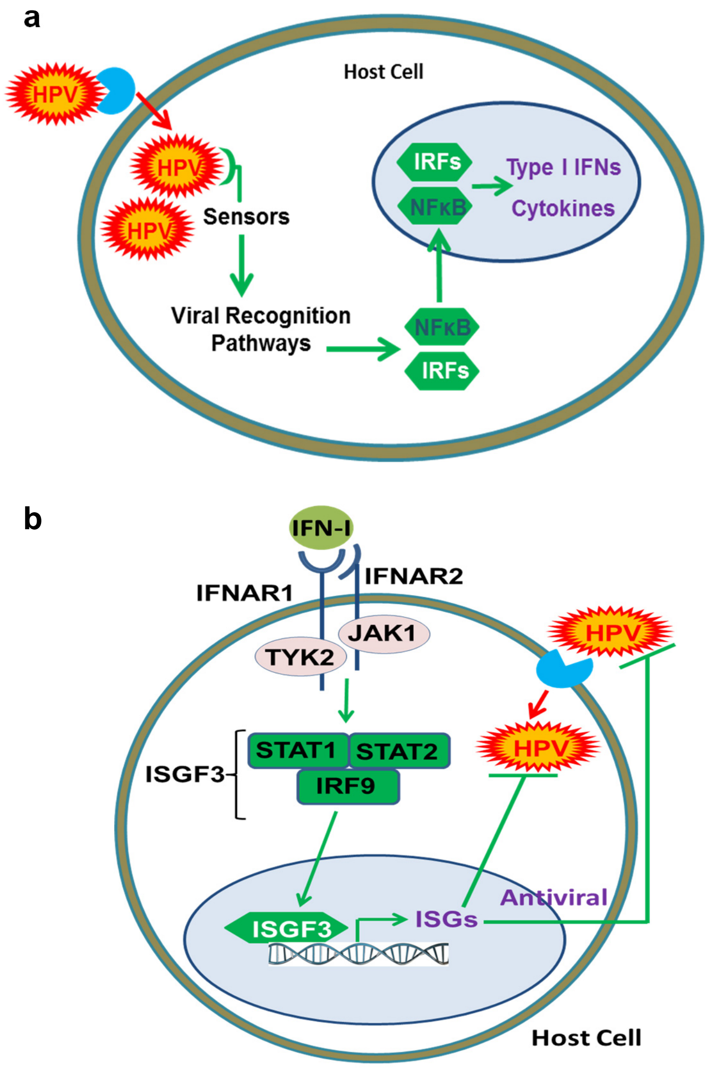

Figure 7. Schematic illustrations showing type I IFN-mediated antiviral signaling pathway. Related to Figures 4 and 5. a) Pathogen associated molecular patterns (PAMPs) of HPV are recognized by the pattern recognition receptors (PRRs) of HPV infected cells. The recognition of PAMPs initiates a series of signaling cascades that leads to the activation of interferon regulatory factor 3 (IRF3), which triggers expression of antiviral type I interferons (such as IFN-α and IFN-β). **b)** upon binding to their receptors, type I IFNs Activates JAK kinases and phosphorylate STATs. Phosphorylated STAT1 and STAT2 can bind to IRF9 to form a complex named 'IFN-Stimulated Gene Factor 3' (ISGF3), which translocates into the nucleus and initiates the transcription of a large spectrum of interferon-stimulated genes (ISGs) in virus infected cells as well as neighbor health cells. ISGs are involved in almost all key steps of antiviral effects in host cells, including inhibition of virus entry, blockade of virus replication, and obstruction of viral egress.

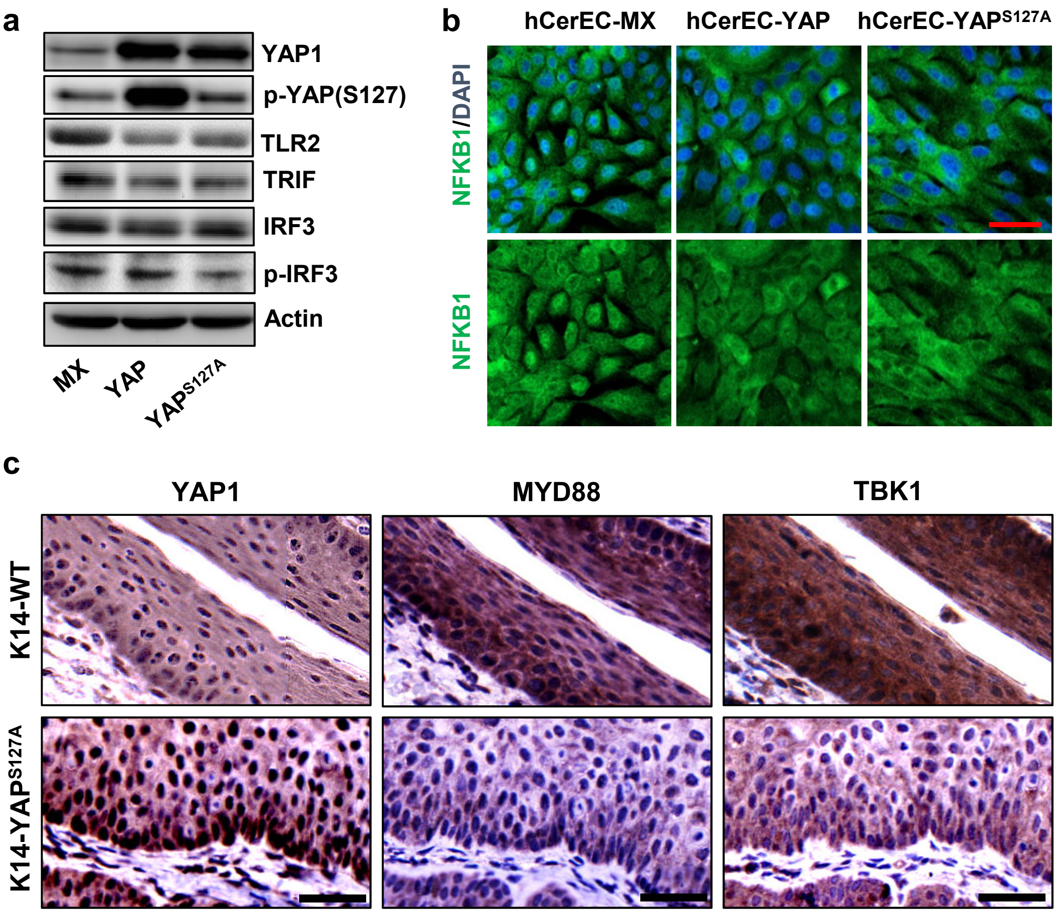

Figure S8. Hyperactivation of YAP1 in cervical epithelial cells inactivates signaling pathways involved in type I interferon production *in vitro* and *in vivo*. Related to Figures 4. a) Representative western blots from three independent experiments showing protein levels of YAP, TLR2, TRIF, and IRF3 in hCerEC-MX (control), hCerEC-YAP and hCerEC-YAP^{S127A} cells. **b**) Representative images showing the expressions and locations of NFκB1 in hCerEC-MX (control), hCerEC-YAP and hCerEC-YAP^{S127A} cells. NFκB1 proteins were visualized using an Alexa-488 (green) conjugated secondary antibody. Nuclei were stained with DAPI (blue). Scale bar: 50 µm. **c**) Representative high-resolution images showing expressions of MYD88 and TBK1, which are key components of the type I interferon production pathway, in cervical tissues of control and KRT14-YAP^{S127A} transgenic mice. Expression of proteins were detected by alkaline phosphatase-based detection systems using a VECTASTAIN® ABC-AP Staining Kit and antigens were visualized with a VECTASTAIN ELITE ABC kit (see details in Materials and Methods). The nuclei were counterstained with hematoxylin. Scale bar: **30** µm.

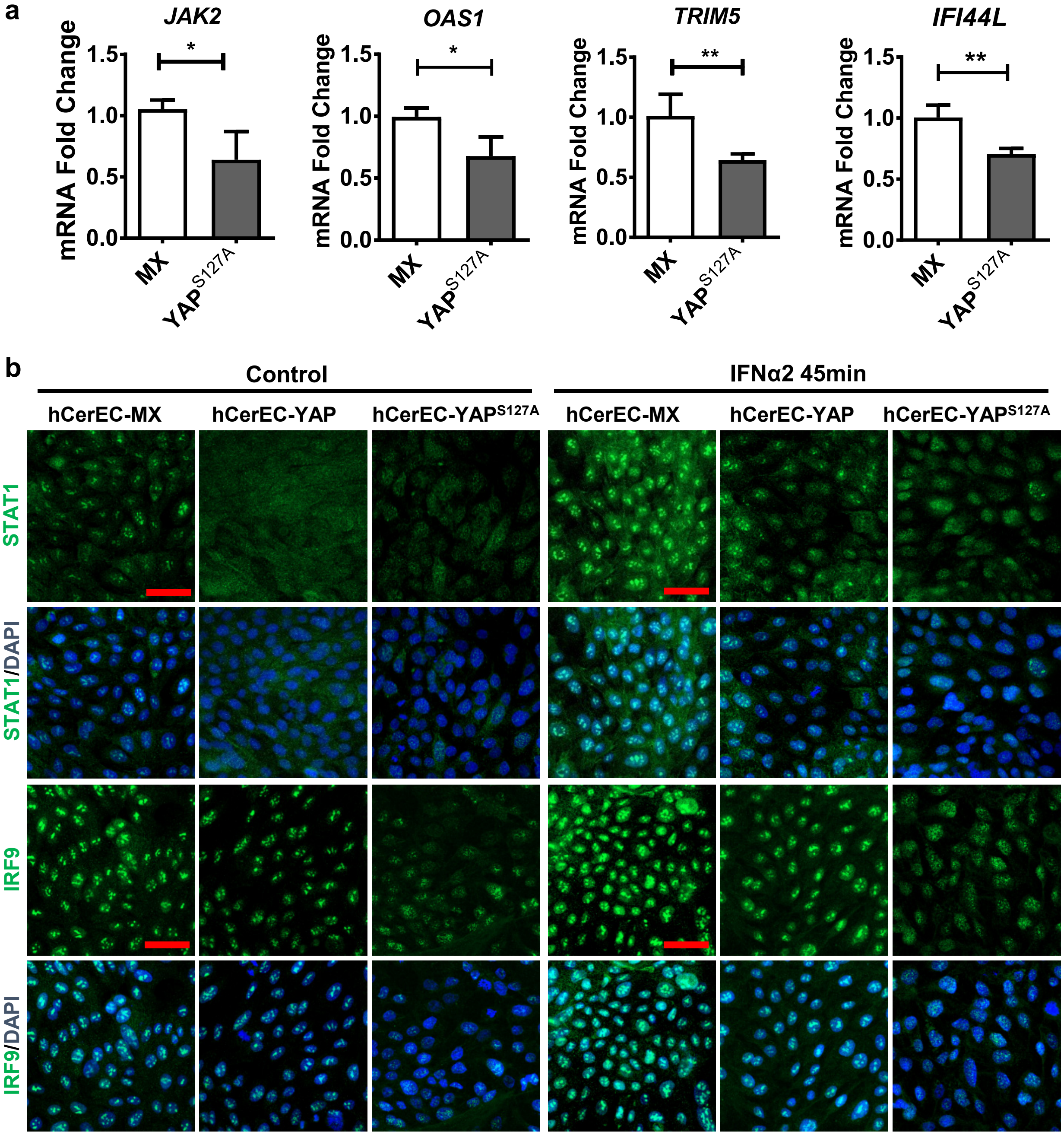

Figure S9. Hyper-activation of YAP1 suppresses the IFNs/JAKs/STATs signaling pathway in cervical epithelial cells. Related to Figure 5. a) Quantitative data showing mRNA levels of JAK2 and several interferon-stimulated genes (ISGs) in hCerEC-MX (control) and hCerEC-YAP^{S127A} cells. Each bar represents the mean ± SEM (n = 4). *: $p < 0.05$; **: $p < 0.01$; ***: $p < 0.001$. **b**) Representative images showing the expressions and locations of STAT1 and IRF9 in hCerEC-MX (control), hCerEC-YAP and hCerEC-YAP^{S127A} cells in the presence or absence of IFNα2b. STAT1 and IRF9 proteins were visualized using an Alexa-488 (green) conjugated secondary antibody. Nuclei were stained with DAPI. Scale bar: 20 µm.

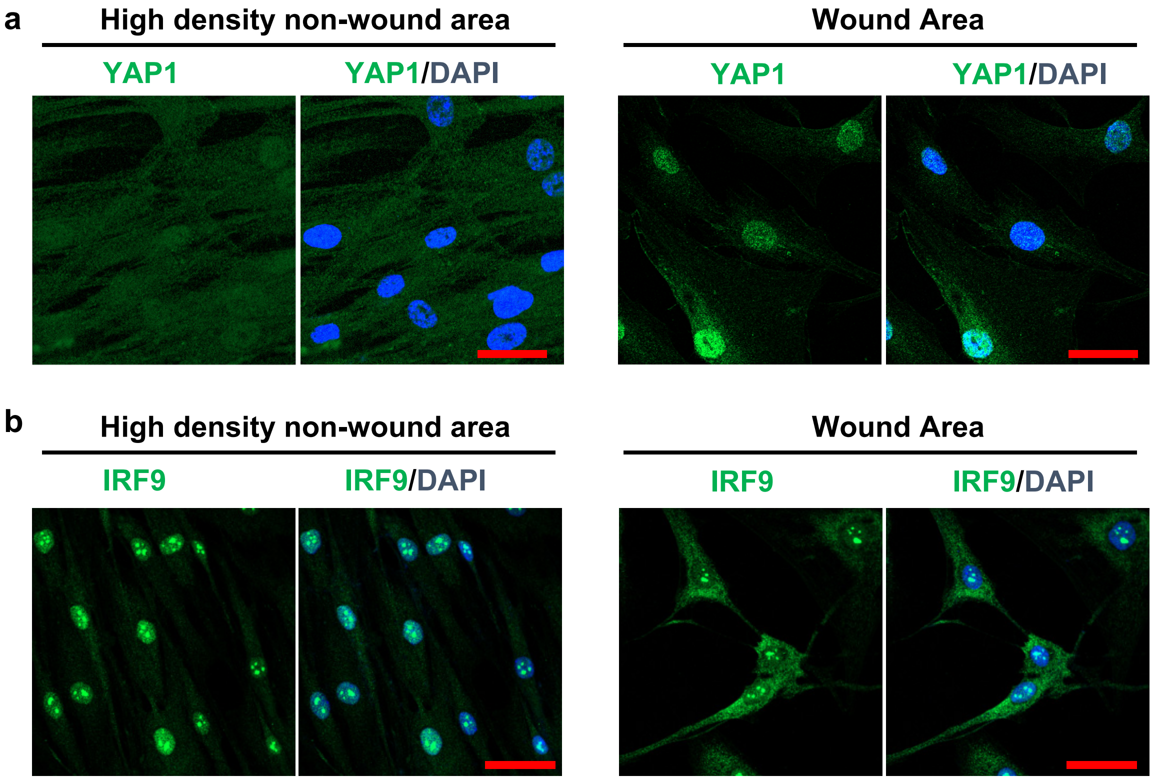

Figure S10. Hyperactivation of YAP1 in cervical epithelial cells inactivated signaling pathways involved in type I interferon production. Related to Figure 5. a) Representative images from three independent *in vitro* experiments showing expressions and locations of YAP1 in normal and "wound" area in cultured hCerEC cells. YAP1 protein were visualized using an Alexa-488 (green) conjugated secondary antibody. Nuclei were stained with DAPI (blue). Scale bar: 20µm. **b**) Representative images from three independent *in vitro* experiments showing expressions and locations of IRF9 in normal and "wound" area in cultured hCerEC cells. IRF9 protein were visualized using an Alexa-488 (green) conjugated secondary antibody. Nuclei were stained with DAPI (blue). Scale bar: 30µm.

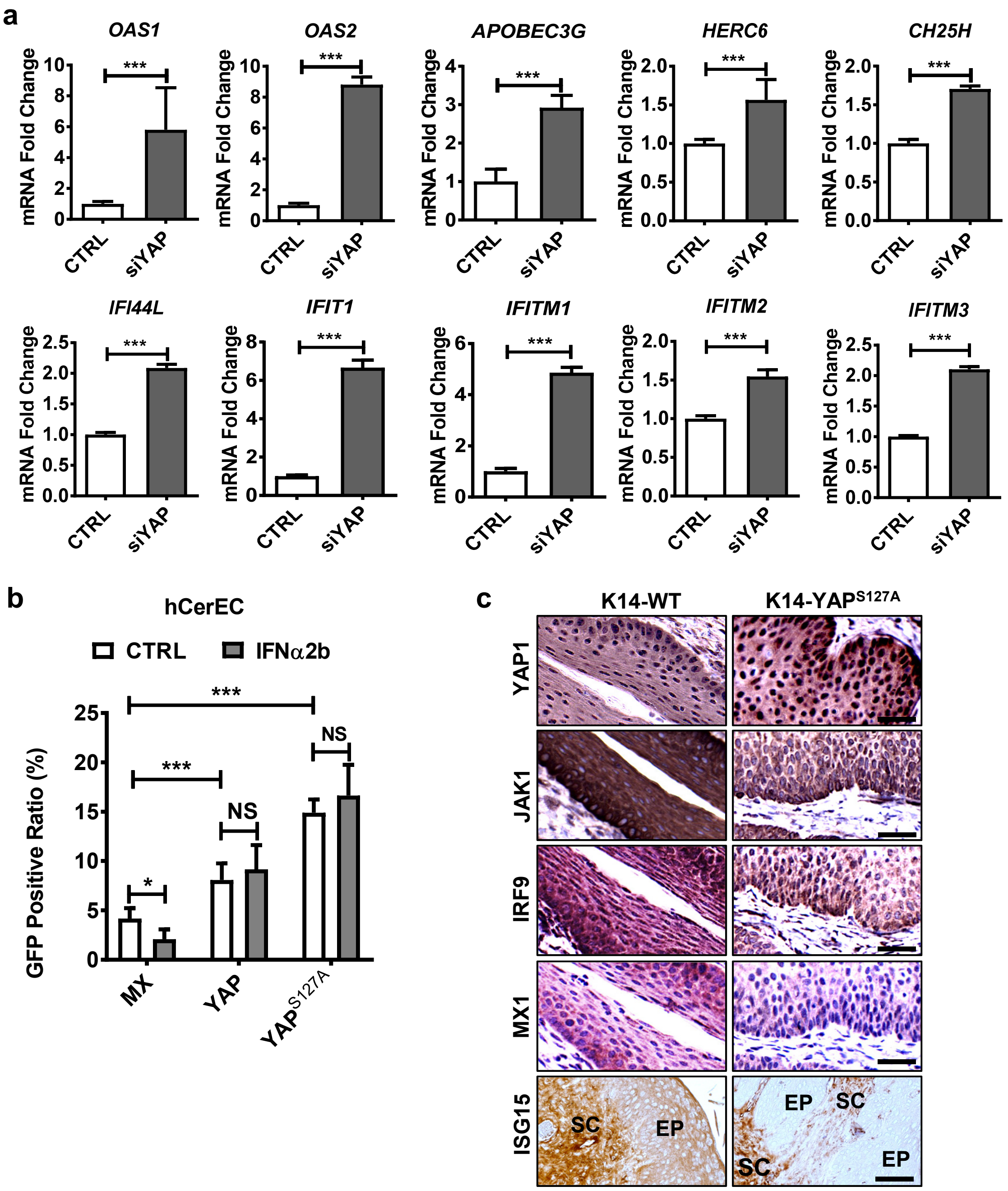

Figure S11. YAP1 suppresses the expression of key components of the JAKs-STATs pathway and antiviral ISGs in cervical epithelial cells *in vitro* and *in vivo*. Related to Figure 5. a) Quantitative data showing mRNA levels of several well-known antiviral ISGs. Each bar represents the mean ± SEM (n = 4). ***: $p < 0.001$. **b)** Quantitative data of figure **5D** showing the percentage of the GFP positive cells (HPV PsV infected cells) in hCerEC-MX (control), hCerEC-YAP, and hCerEC-YAP^{S127A} cells with or without pretreatment with IFNα2b. Each bar represents the mean ± SEM (n = 5). NS: no significant difference; *: $p < 0.05$; ***: $p < 0.001$. **c**) Representative high-resolution images showing YAP1-suppressed expressions JAK1 and IRF9, which are key components of the JAK1 - STATs signaling pathway, and MX1 and ISG15, which are well-studied downstream antiviral proteins of the JAK1-STATs signaling pathway, in cervical tissues of control and KRT14-YAP^{S127A} transgenic mice. Expression of proteins were detected by alkaline phosphatase-based detection systems using VECTASTAIN® ABC-AP Staining Kit and antigens were visualized with VECTASTAIN ELITE ABC kit (see details in Materials and Methods). The nuclei were counterstained with hematoxylin. SC: stroma cells; EP: Epithelial cells. Scale bar: 30 µm.

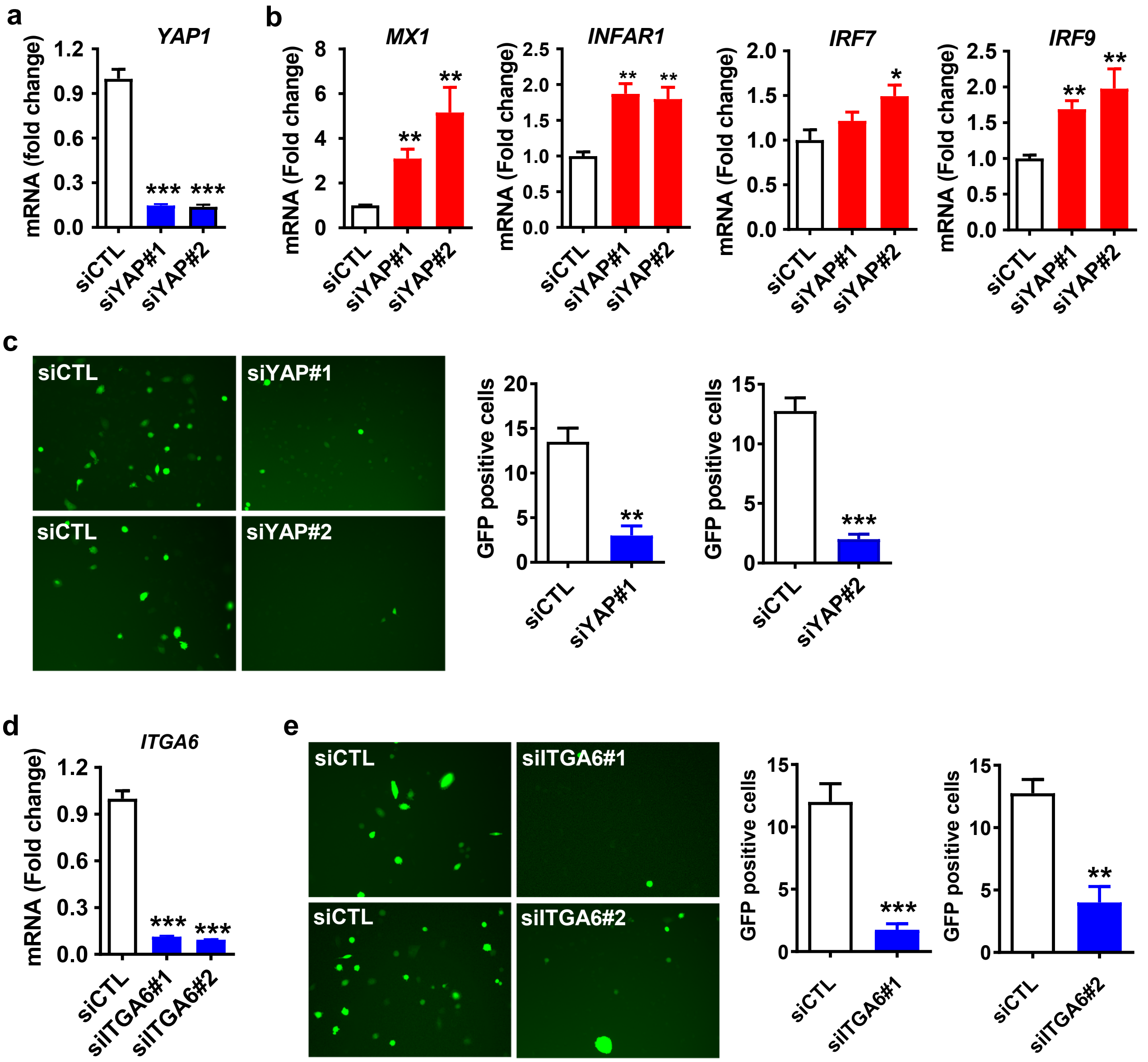

Figure S12. Validation of *YAP1* and *ITGA6* siRNAs. Related to STAR methods. a) Quantitative PCR results showing *YAP1* mRNA levels in SiHa cells transfected with control siRNA (siCTL) or *YAP1*-specific siRNAs (siYAP#1 and siYAP#2). Noting *YAP1* expression was significantly reduced by both siYAP#1 and siYAP#2. **b)** Quantitative PCR results showing relative mRNA levels of *MX1, INFAR1, IRF7,* and *IRF9* in SiHa cells transfected with control siRNA (siCTL) or *YAP1* targeting siRNAs (siYAP#1 and siYAP#2). *: $P < 0.05$; **: $P < 0.01$; ***: $P < 0.001$. **c**) Representative images showing HPV16 Pseudovirion (PsV)-derived GFP signal in SiHa cells transfected with siCTL or siYAP#1/siYAP#2. Bar graphs on the left showing GFP-positive cells (n ≥ 7). Noting knockdown of *YAP1* significantly decreased HPV PsV infectivity. **: $P < 0.01$; ***: $P < 0.001$. **d**) Quantitative PCR results showing relative levels of *ITGA6* mRNA in SiHa cells transfected with control siRNA (siCTL) or *ITGA6* targeting siRNAs (siITGA6#1 and siITGA6#2). *ITGA6* mRNA level was significantly reduced by both siITGA6#1 and siITGA6#2. ***: $P < 0.001$. **e**) Representative images showing HPV16 PsV-derived GFP signal in SiHa cells transfected with siCTL or siITGA6#1/siITGA6#2. Bar graphs on the left showing quantitative results. Knockdown of *ITGA6* significantly decreased HPV PsV infection. **: $P < 0.01$; ***: $P < 0.001$.

Table S1. Primers used in this study*, Related to STAR methods (Mouse model studies & Quantitative Real Time-PCR*)***.

Genotyping primers for KRT14-E6 / KRT14-E7 Mice. To differentiate the E6 genotype from E7 genotype, the PCR products are digested with *HpaI*. Mice with E6 genotype will result in two PCR products that are of similar sizes, while mice with E7 genotype will result in a PCR product that is 50 nucleotides shorter.		
Primers	Sequences (5' to 3')	Product size
Forward	GGCGGATCCTTTTATGCACCAAAAGAGAACTG	800 bps
Reverse	CCCGGATCCTACCTGCAGGATCAGCCATG	
Internal control A	TATACTCAGAGCCGGCCT	250bps
Internal control B	ACAGCGTGGTGGTACCTTAT	
Internal control C	TCC TCG TGC TTT ACG GTA TC	
**Genotyping primers for KRT14-rtTA mice**		
Primers	Sequences (5' to 3')	Product size
Forward	CACGATACACCTGACTAGCTGGGTG	400 bps
Reverse	CATCACCCACAGGCTAGCGCCAACT	
Internal control A	CCATGTCTAGACTGGACAAGA	150bps
Internal control B	GTCAGTCGAGTGCACAGTTT	
**Genotyping primers for Tet-YAP^{S127A} mice**		
Primers	Sequences (5' to 3')	Product size
Forward Primer:	GACTCGGAGACCGACCTGGA	550 bps
Reverse Primer:	CATCATATTCTGCTGCACTGGTGGAC	
Internal control A	CCATGTCTAGACTGGACAAGA	150bps
Internal control B	GTCAGTCGAGTGCACAGTTT	
**Primers for qRT-PCR**		
Genes	Forward primer sequences	Reverse primer sequences
TLR2	GGCCAGCAAATTACCTGTGTG	CTGAGCCTCGTCCATGGGCCACTCC
TLR4	TGCAATGGATCAAGGACCAGAGGC	GTGCTGGGACACCACAACAATCACC
MyD88	CGGCAACTGGAGACACAAG	TCTGGAAGTCACATTCCTTGC
TRIF	GCAGCCCCGGATCCCT	TGTCCCCTACCCATTCACTGTT
IRF1	GCCAGTCGACGAGGATGAGGAAGGGAA	CCAGCGGCCGCCTGCTACGGTGCACAGG
IRF2	CCAGTCGACTACCTCAGCAACATGGGG	CCAGCGGCCGCGGCTTAACAGCTTGAC
IRF3	CGGAAGCTTCTGAAGCGGCTGTTGGTG	GTGCTCGAGACCATGAGGAGCGAGGGC
IRF4	CCAGTCGACGCAAGCTCTTTGACACAC	CCAGCGGCCGCCTTTTCATTCTTGAATAG
IRF5	GCCTTGTTATTGCATGCCAGC	AGACCAAGCTTTTCAGCCTGG
IRF7	TGCAAGGTGTACTGGGAG	TCAAGCTTCTGCTCCAGCTCCATAAG
IRF9	TTCTGTCCCTGGTGTAGAGCCT	TTTCAGGACACGATTATCACGG
NFkB1	CACTGCTCAGGTCCACTGTC	CTGTCACTATCCCGGAGTTCA
NFKB2	GGGGCATCAAACCTGAAGATTTCT	TCCGGAACACAATGGCATACTGT
IFNAR1	CAGCACCTGATGGCCTATCAC	TGGAGCATGAAGAACTGGATG
IFNAR2	CATCCACCTGAATGCCTACTTCT	TCTGCAAGGAGTCACCATTCTCT
STAT1	GGCACCAGAACGAATGAGGG	CCATCGTGCACATGGTGGAG
STAT2	GCAGCACAATTTGGGAA	ACAGGTGTTTCGAGAACTGGC
CH25H	ATCACCACATACGTGGGCTTT	GTCAGGGTGGATCTTGTAGCG
TRIM5	AGGAGTTAAATGTAGTGCT	ACCATGGATTTCTCATCTAT

IFI44L	GCTGCGGGCTGCAGAT	CTCTCTCAATTGCACCAGTTTCC
HERC6	CACTACCACTCCCTGGCATT	TGTTACTTCCCCAGCCAAAV
MX1	GTGCATTGCAGAAGGTCAGA	TCAGGAGCCAGCTTAGGTGT
ISG15	TCCTGCTGGTGGTGGACAA	TTGTTATTCCTCACCAGGATGCT
ISG20	ATCTCTGAGGGTCCCCAAGGA	TTCAGTCTGACACAGCCAGGCG
OAS1	TGATGCCCTGGGTCAGTTG	TCGGTGCACTCCTCGATGA
IFIT1	TCTCAGAGGAGCCTGGCTAA	CCAGACTATCCTTGACCTGATGA
IFITM1	GGATTTCGGCTTGTCCCGAG	CCATGTGGAAGGGAGGGCTC
IFITM2	ATTGTGCAAACCTTCTCTCCTG	ACCCCCAGCATAGCCACTTCCT
IFITM3	ACTGTCCAAACCTTCTTCTCTC	AGCACAGCCACCTCGTGCTC
IFITM5	TTGATCTGGTCGGTGTTCAG	GTCAGTCATAGTCCGCGTCA
IFNA	GTACTGCAGAATCTCTCCTTTCTCCTG	GTGTCTAGATCTGACAACCTCCCAGGCACA
IFNB	TTGTGCTTCTCCACTACAGC	CTGTAAGTCTGTTAATGAAG
YAP1	GCAACTCCAACCAGCAGCAACA	CGCAGCCTCTCCTTCTCCATCTG
ITGA6	ATTCAGGAGTAGCTTGGTGGAT	TTCTCTTGAAGAAGCCACACTTC
SDC1	GCTGACCTTCACACTCCCCA	CAAAGGTGAAGTCCTGCTCCC
EGFR	GAGGGTGCTCTTAGCCACAG	CATGGTGAGGGCTGAGGTGA
TBK1	CAACCTGGAAGCGGCAGAGTTA	ACCTGGAGATAATCTGCTGTCGA
JAK1	CTGTACGATGAGAGCACCAAGC	CGTGCCAGTTGGTAAAGTAGAACC
JAK2	TAAAGCACACAGAAACTATTCAGAGTC	AGAATATTCTCGTCTCCACAAAC
APOBEC3G	TCAGAGGACGGCATGAGACTTAC	AGCAGGACCCAGGTGTCATTG
RNA18S	CGCCGCTAGAGGTGAATTC	TTGGCAAATGCTTTCGCTC
GAPDH	GAGTCAACGGATTTGGTCGT	GACAAGCTTCCCGTTCTCAG

* Human JAK/STAT Signaling QT-PCR Primer Library (Item# HJAK-I) and Human Toll-like Receptor Signaling QT-PCR Primer Library (Item# HTLR-I) were purchased from Real Time Primers, LLC (Elkins Park, PA). Gene sets of these libraries could be obtained at: http://realtimeprimers.com/hujasiprli.html, and http://www.realtimeprimers.com/hutoresiprli.html.
